# Proteomic Analyses of *Agkistrodon contortrix contortrix* Venom Using 2D Electrophoresis and MS Techniques

**DOI:** 10.3390/toxins8120372

**Published:** 2016-12-13

**Authors:** Aleksandra Bocian, Małgorzata Urbanik, Konrad Hus, Andrzej Łyskowski, Vladimír Petrilla, Zuzana Andrejčáková, Monika Petrillová, Jaroslav Legáth

**Affiliations:** 1 Faculty of Chemistry, Rzeszow University of Technology, Powstańców Warszawy 6, 35-959 Rzeszów, Poland; murbanik92@wp.pl (M.U.); knr.hus@gmail.com (K.H.); andrzej.lyskowski@prz.edu.pl (A.L.); jlegath@prz.edu.pl (J.L.); 2 Department of Physiology, University of Veterinary Medicine and Pharmacy, Komenského 73, 041 81 Košice, Slovak Republic; petrillav@gmail.com (V.P.); zuzka.kravcova@gmail.com (Z.A.); 3 Zoological Department, Zoological Garden Košice, Široká 31, 040 06 Košice-Kavečany, Slovac Republic; 4 Department of General Education Subjects, University of Veterinary Medicine and Pharmacy, Komenského 73, 041 81 Košice, Slovak Republic; monika.petrillova@uvlf.sk; 5 Department of Pharmacology and Toxicology, University of Veterinary Medicine and Pharmacy, Komenského 73, 041 81 Košice, Slovak Republic

**Keywords:** *Agkistrodon contortrix contortrix*, southern copperhead, venom, proteomics

## Abstract

Snake venom is a complex mixture of proteins and peptides which in the Viperidae is mainly hemotoxic. The diversity of these components causes the venom to be an extremely interesting object of study. Discovered components can be used in search for new pharmaceuticals used primarily in the treatment of diseases of the cardiovascular system. In order to determine the protein composition of the southern copperhead venom, we have used high resolution two dimensional electrophoresis and MALDI ToF/ToF MS-based identification. We have identified 10 groups of proteins present in the venom, of which phospholipase A_2_ and metalloprotease and serine proteases constitute the largest groups. For the first time presence of 5′-nucleotidase in venom was found in this group of snakes. Three peptides present in the venom were also identified. Two of them as bradykinin-potentiating agents and one as an inhibitor.

## 1. Introduction

Southern copperhead (*Agkistrodon contortrix contortrix*) found in the forests of southeastern part of the USA is one of the most common venomous snakes of this country. Although this species is venomous, its venom is quite gentle as compared to the other snakes in the same areas and usually is not considered a threat to an adult person. The bite causes local pain, swelling, erythema, nausea, vomiting, thrombocytopenia, hypotension, and sometimes anaphylactic shock [1,2,3]. Southern copperhead venom toxins cause: the deterioration of homeostasis and cell adhesion, activation of a coagulation cascade or blockade of some of its factors, and miotoxicity that causes necrosis of the muscles. The spread of the venom components allow for the dissolution of fibrin clots by the fibrinolytic toxin. Tissue damage occurs by damage to endothelial cells, mostly caused by metalloproteinases and phospholipases A_2_.

The main components of the *A. c. contortrix* venom are metalloproteinases (SVMPs), phospholipases A_2_ (PLA_2_), and serine proteases [4]. Metalloproteinases are responsible for the formation of edema, hemorrhage, inflammatory changes, and necrosis of cells. Their function is to interfere with homeostasis on different levels. They cause degradation of extracellular matrix, leading to poor adhesion of endothelial cells, as well as cleavage of large proteins such as fibrinogen [5,6]. Phospholipases A_2_ exhibit mio-, neuro-, and hemotoxic properties. They cause local and systemic degeneration of the skeletal muscles by interfering with the integrity of the cell membrane. Their neurotoxic activity is based on blocking acetylcholine receptors and consequently inhibition of neuromuscular transmission. Hemotoxic activity of phospholipases consist of inhibition of blood coagulation factor cascade [7]. Serine proteases also affect the coagulation system of the victim by acting on components of blood coagulation, fibrinolysis, and platelets, causing an imbalance of homeostasis [8].

In the venom of the southern copperhead, l-amino acid oxidases (LAAO), cysteine-rich proteins (CRISPs), and C-type lectins also occur in small amounts [4]. The former are responsible for platelet aggregation, edema, and hemorrhage. They cause platelet aggregation but can also inhibit this process. LAAOs lead to apoptosis of vascular endothelial cells, also in some tumor cell lines. All effects are related to the ability of l-amino acid oxidases to produce hydrogen peroxide during catalyzed reaction of amino acid oxidation [7]. Cysteine-rich proteins in turn, cause a lock of the calcium and potassium channels and, consequently, inhibition of smooth and skeletal muscles contraction and blockage of blood vessels. [9,10]. C-type lectins are responsible for the agglutination of red blood cells because they have the ability to bind carbohydrate moieties located on their surface [11].

Venoms of all *Agkistrodon* species have similar proteolytic and phospholipolytic potential, while the miotoxicity of *Agkistrodon contortrix contortrix* venom is the weakest of all pit vipers of the New World [4].

Many venom proteins are highly toxic but weakly immunogenic. A low content of antibodies directed against such proteins in antivenoms result in the necessity of using very high doses of antitoxin, which may be dangerous to the patient. Therefore, the knowledge of precise venom composition and of ontogenetic, individual, and geographic venom variability may have a positive effect on the treatment of bite victims and in the selection of specimens for the generation of improved antidotes. Moreover, complex knowledge about venom composition, common and unique antigenic determinants, and their reactivity with antibodies may in the future lead to defining the minimal set of venoms containing all epitopes necessary to generate therapeutic broad-range polyvalent antiserum [12].

Two-dimensional electrophoresis has been repeatedly used for the analysis of venom proteins. This technique can be used to compare proteomes of different species [13], investigate the influence of factors like sex [14], the area of occurrence [15], or to determine whether post-translational modifications occurred in proteins [16]. The combination of 2DE and Western blot helps in clinical pathology of snake bites and antitoxins mechanisms studies [17]. Moreover, the 2D technique is more suitable for high-molecular mass protein examination and protein post-translational modification discovery.

The presented work includes a complete proteomic analysis of the southern copperhead venom. For the first time, the proteins were separated using high resolution two-dimensional electrophoresis and identified, together with low weight peptides, on a MALDI ToF/ToF mass spectrometer.

## 2. Results

### 2.1. Proteome Analysis

**Figure 1:**
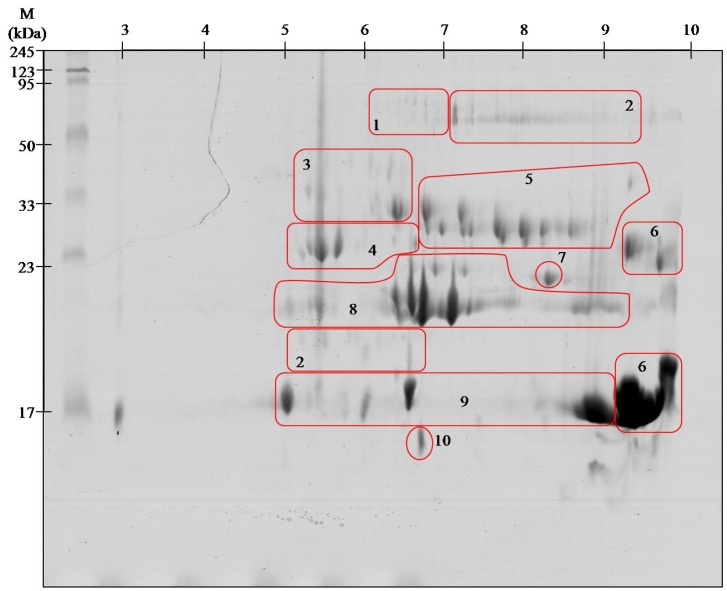
Representative 2-D protein maps obtained from southern copperhead venom with identified protein groups shown. 1. Snake venom 5′-nucleotidase; 2. l-amino-acid oxidase; 3. Beta-fibrinogenase; 4. Thrombin-like proteins; 5. Protein C activator; 6. Basic phospholipase A2; 7. Cysteine-rich venom protein; 8. Snake venom metalloproteinase; 9. Acidic phospholipase A_2_; 10. C-type lectin. The proteins were separated by isoelectrofocusing at pH range 3–10, then distributed on polyacrylamide gels by SDS-PAGE and stained with colloidal Coomassie Brilliant Blue G-250. Molecular weight (MW) and pH 3–10 scale are shown.

**Figure 2:**
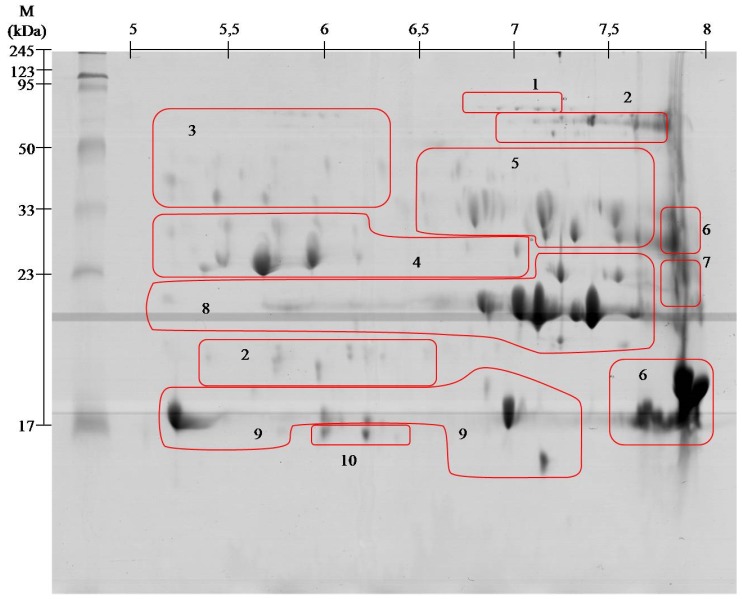
Representative 2-D protein maps obtained from southern copperhead venom with identified protein groups shown. 1. Snake venom 5′-nucleotidase; 2. l-amino-acid oxidase; 3. Beta-fibrinogenase; 4. Thrombin-like proteins; 5. Protein C activator; 6. Basic phospholipase A_2_; 7. Cysteine-rich venom protein; 8. Snake venom metalloproteinase; 9. Acidic phospholipase A_2_; 10. C-type lectin. The proteins were separated by isoelectrofocusing at pH range 3–10, then distributed on polyacrylamide gels by SDS-PAGE and stained with colloidal Coomassie Brilliant Blue G-250. Molecular weight (MW) and pH 3–10 scale are shown.

To obtain the highest resolution of gels, proteins were focused in two pH ranges: 3–10 and 5–8 in the first dimension. On the gel separated at pH range 3–10, 119 spots were found, whereas in the pH range 5–8, 116 spot were found. All spots were excised from the gel, digested with trypsin, and identified on the MALDI ToF/ToF mass spectrometer. Since the identical identification was achieved in several spots in a specific area on the gels, spots containing the same proteins were grouped (Figure 1 and Figure 2). The group included proteins found in spots of similar mass or mass and pI. All spots located on the gels were focused in the range from 5 to 10.

The results for protein identification are summarized in Table 1. The proteins are grouped into 10 groups containing from one to several spots.

**Figure 3:**
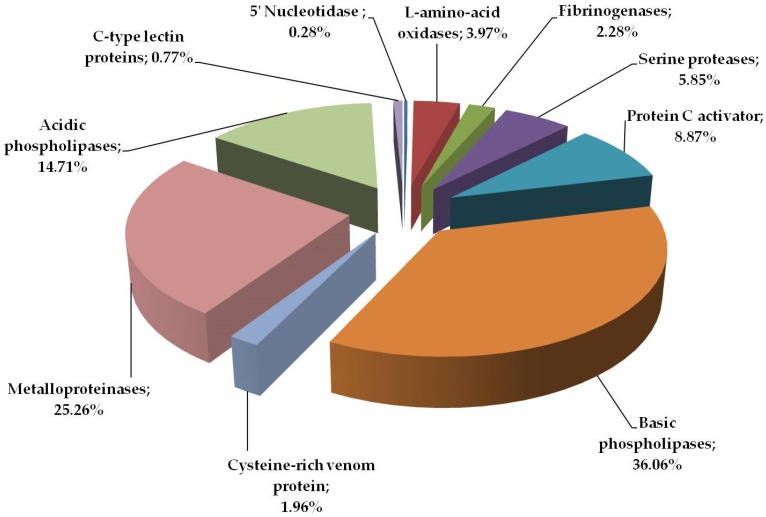
Percentage of protein amount in groups of *Agkistrodon contortrix contortrix* venom calculated on the basis of %Vol of particular spots on gels.

Percentage distribution of protein groups in southern copperhead venom is presented in Figure 3. According to this analysis, including spots area and intensity (%Vol), the most abundant proteins are phospholipases (almost 50%). Other groups containing a significant amount of protein are metalloproteinases and peptidase S1 family, including protein C activator, serine proteases (thrombin-like proteins) and fibrinogenases. The least abundant proteins in the analyzed venom are 5′ nucleotidase and C-type lectin proteins, both less than 1%.

### 2.2. Peptidome Analysis

**Figure 4:**
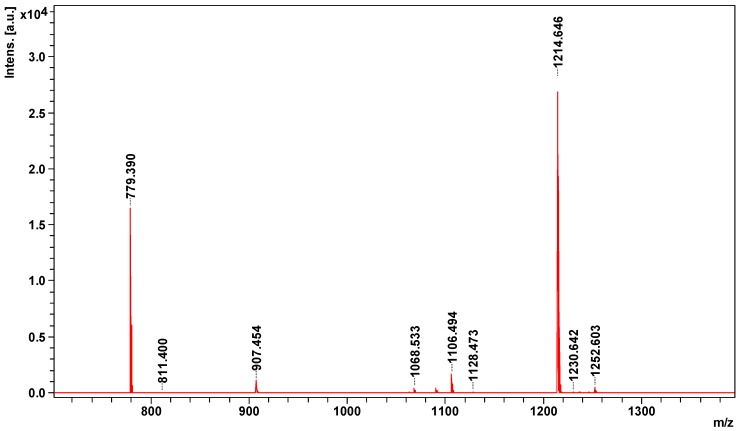
Mass spectrum of peptidome fraction of southern copperhead venom obtained on MALDI ToF/ToF mass spectrometer.

MS spectrum obtained on MALDI ToF/ToF mass spectrometer contains nine signals of potential peptides in the range of *m*/*z* 779–1253 (Figure 4).

All potential peptides were sequenced in LIFT mode. For parent ion 779.3898 *m*/*z* 42 signals were obtained in the fragmentation spectrum, for 907.4536 *m*/*z*—50, for 1063.5343 *m*/*z*—72, 1068.5329 *m*/*z*—76, for 1214.6465 *m*/*z*—69, 1230.6419 *m*/*z*—118, 1236.6326 *m*/*z*—200, for 1246.6355 *m*/*z*—72, and for 1252.6025 *m*/*z*—90 signals. Sequences of three peptides obtained from SwissProt and NCBInr data bases are summarized in Table 2.

## 3. Discussion

Viperid venoms may contain up to 100 proteins belonging to a small number of protein families [18]. Obtained 2DE gels of *A. contortrix contortrix* venom proteins contain even greater number of spots. However, the identification using MALDI ToF/ToF showed that they belong only to 10 major families. With high probability, it can be assumed that the proteins in this venom are highly post-translationally modified, as shown by clearly visible spots trains in gels (Figure 1 and Figure 2). This phenomenon is characteristic for Viperidae family and was described already several times [13,16].

Our study indicates that the composition of the analyzed venom differs from that described in previous reports. We have observed a significantly higher share of phospholipases A_2_ than metalloproteinases. Earlier the presence of these two groups in almost equal amounts was reported [4], whereas in our case there is twice as much phospholipases than metalloproteinases. These differences may be due to many factors: gender, age of snakes, geographical origin or type of food [19]. As shown in our previous work, the differences may also occur when other analytical techniques are used to analyze the venom composition (RP-HPLC + SDS-PAGE vs. 2DE) [20]. As described before, procedure combining HPLC and SDS-PAGE allows for recovery of all venom components in the broad molecular mass range, while it cannot be achieved by conventional 2D-SDS-PAGE. However, the advantage of this protocol is mostly visible in the class of small proteins and peptides [21]. Furthermore, 2D electrophoresis allows the observation of post-translational modifications, which can be crucial for the immunogenicity of proteins [13]. However, in our opinion the observed differences arise from analyzed venom composition and not the analytical techniques used. There is no published comparison of those two techniques on the same venom sample. Hence, it is difficult to clearly determine what factor most significantly affects the observed differences.

Our research clearly indicates intraspecific variation of the protein composition of *Agkistrodon contortrix contortrix* venom. This information is extremely valuable for creating reference samples of the venom used in the production of antisera. This information should be taken into account so that the antiserum has the widest possible spectrum of activity [22]. On the other hand, this knowledge can be useful for physicians to treat the symptoms of bitten patients. It is known that, in North American Viperidae snakes, there is an inverse relationship between toxicity of the venom and the content and activity of metalloproteinases [12]. This means that different people bitten by the same snake species may exhibit different levels of exacerbation of symptoms. Our results indicate that our specimens of *A. contortrix contortrix* may produce more toxic venom, because the content of the metalloproteinases is clearly lower [4]. Therefore, knowledge about the diversity of venom and various spectrum of the potential effect on the human body may help in treatment of individual patients.

The most interesting issue is a detection of 5′-nucleosidase in the southern copperhead venom, whose presence has never been observed before in American species of *Agkistrodon*.

5′-nucleotidases have been described in many species of snakes including Viperidae [23,24,25]. Although these proteins are present in the venom in small quantities they are observed in a number of isoforms arising inter alia from the tendency to form oligomers. This fact, as well as the mass and pI often close to the other components of the venom, cause the isolation of a homogeneous protein extremely difficult [24,26]. 5′-nucleotidases act as co-factors for hemorrhagic toxins and inhibit platelet aggregation by releasing the adenosine from the GMP and AMP, which binds to the receptors on the platelets [24]. Thus, these proteins act synergistically to phospholipases A_2_ and disintegrins enhancing anticoagulant effect of the venom [27,28,29]. 5′-nucleotidases are an unexplored group of proteins, both in terms of toxicological and pharmacological effects. However, they are a group of anti-coagulant factors that potentially could be used in medicine [26].

The largest group of proteins of *Agkistrodon contortrix contortrix* venom are the phospholipases A2 (PLA_2_)—almost 50%. Enzymes differing in their isoelectric point have been identified, described as acidic (spots # 9) and basic (# 6), showing a similar molecular weight (Figure 1 and Figure 2). All identified PLA_2_ proteins are secreted (sPLA_2_) and belong to group II (GII), characteristic for Viperidae [7]. Most phospholipases A_2_ present in the venom have similar amino acid sequence and three-dimensional structure, but broad spectrum of activity: from neuro-, cardio-, and myotoxic, through hemolytic, anti-coagulant, anti-platelet, and tissue damaging properties [30]. Anticoagulant properties of PLA_2_ result from the inhibition of coagulation complex formation, mainly through hydrolysis of phospholipids-induced inhibition of the intrinsic tenase complex [31]. These proteins also directly affect platelet activity. In low concentrations they initiate aggregation, but in high concentrations they act as inhibitors [32]. The presence of such a large amount of proteins from this group is not surprising as it has been reported many times that it is one of the largest groups in the venom of Viperidae [4,20,33].

Metalloproteinases (SVMPs) are present in large quantities mainly in the viperid venom, but also in some elapid and colubrid [34]. In the studied venom, it is the second largest group (approx. 25%). SVMPs are divided into three basic groups (from P-I to P-III) based on the domain from which the protein is composed [35]. In the venom of *Agkistrodon contortrix contortrix*, we found metalloproteases belonging to all three groups, and thus they include all three domains characteristic of these proteins: catalytic metalloproteinase, disintegrin, and a Cys-rich domain. The spectrum of activity of these enzymes is very broad, but all lead to the disorders of hemostasis. The most important are: activation of prothrombin and factor X, lysis of fibrin and fibrinogen, inhibition of platelet aggregation, hemorrhagic effect, and eliminating inhibitors of serine proteases [36].

All proteases identified in the venom of the southern copperhead (outside metalloproteinases) belong to the class PA family S1 of trypsin-like serine proteinases (SVSPs) [37]. Among snake venom serine proteinases we identified three groups of enzymes: beta-fibrinogenase (#3), thrombin like proteins (#4), and protein C activator (#5) (Figure 1 and Figure 2, Table 1). *A. c. contortrix* fibrinogenases are responsible for the fibrino- and fibrinogenolytic properties of venom and, unusually for this group of species, prefer cutting off fibrinopeptide B. They can also cut the γ chain, responsible for crosslinking [38]. Thrombin-like enzymes, despite the relatively low similarity to the sequence of thrombin (30%), are able to clot fibrinogen [39]. However, most of these enzymes are capable of releasing only fibrinopeptide A or B, rarely both at once, and therefore the strength of the clot is small and it quickly dissolves [36]. Action of both of the above groups of proteins results in blood being unable to clot, since it lacks a functional fibrinogen [38,39,40,41]. The last protein of this group identified in our experiment is a protein C activator, characteristic for the venom of snakes belonging to the genus *Agkistrodon*. Naturally, protein C is a factor preventing the clotting of blood and it is found in serum in an inactive form. The activation of protein C requires α-thrombin complexed to thrombomodulin or venom protein which selectively cuts through the heavy chain of protein C. Protein C activator is not required for thrombomodulin to act and is the only serine protease which exhibits direct anticoagulant effect [41]. Some reports suggest that even though the southern copperhead venom has a large group of serine proteases, it is not able to coagulate human blood, so these enzymes have no thrombin-like activity [42]. This may mean that the proteins identified on gels in area #4 are sequentially related to enzymes like thrombin, but do not possess the same properties.

L-amino acid oxidases (LAAOs) are widely distributed in Viperidae and Elapidae snake venoms and in *A. c. contortrix* venom represent about 4% of the total proteins (Figure 3). These enzymes catalyze the reaction of oxidative deamination of amino acids and the product of this reaction is hydrogen peroxide [43]. That is what has been implicated by the toxic nature of this group of proteins consisting of the influence platelet aggregation and induce hemorrhage resulting from apoptosis of vascular endothelial cells [44]. Reports indicate that LAAOs can both activate and inhibit platelet aggregation, regardless of the effect, however, in southern copperhead they belong to a group of proteins that disturb homeostasis [36].

The least abundant protein in analyzed venom is 5′-nucleotidase (0.3%). The cysteine-rich venom proteins (CRISPs) and C-type lectins are another example of proteins with very low abundance in analyzed sample, 2% and 0.8% respectively (Figure 3). Both groups have been previously identified in this species, however, in our study, their share is slightly smaller, which is probably the result of much greater phospholipases A_2_ presence [4]. CRISPs are widely distributed in the venoms of the Elapidae, Viperidae, and Colubridae [11] and in the case of Viperidae are rarely found in the venom as isoforms, it is mostly a single protein [20,45]. It is no different in the case of southern copperhead with one clearly visible spot at a height of approx. 25 kDa and a pH in the range of approx. 8 was observed (Figure 1). The exact function of this group of proteins is not known, but numerous studies indicate that they are L-type Ca^2+^ or CNG channel-blocking toxins [11]. In turn, the C-type lectins (CTLs) are proteins devoid of enzymatic activity that bind mono- and oligo-saccharides, mainly galactose, in a calcium-dependent manner [36]. This group of proteins has never been the object of attention of researchers, therefore, the precise role is not well described. However, it is known that they have the ability to agglutinate erythrocytes and stimulate the platelet aggregation [46,47].

In the venom of southern copperhead we have also detected the presence of nine peptides of less than 1300 Da (Figure 4), but sequences of only three of them were identified (Table 2). As with our previous studies [20] two of the peptides are modified by the presence of pyroglutamate residue at the N-terminus, which is typical for the peptides present in the venom [48,49]. All three peptides are responsible for regulating blood pressure and the width of the blood vessel, and two (1063 and 1214 *m*/*z*) have been previously described in other species of *Agkistrodon* [48]. Two identified bradykinin-potentiating peptides are inhibitors of metalloproteases and act as a hypotensive peptides [48,50]. In turn, the third identified peptide with *m*/*z* 1063 is a bradykinin inhibitor peptide, present in many species, clearly evolutionarily conserved [4,51]. This peptide antagonizes the vasodilatory actions of bradykinin at the bradykinin B2 receptor and disrupts the functioning of the cardiovascular system [51]. All identified peptides supplement the overall hemotoxic effect of the venom of the southern copperhead.

Snake venom is a complex mixture of hundreds of proteins and peptides of various properties serving as immobilizing or lethal agents, which also support digestion. Viperidae family have a hemotoxin-rich venom, causing abnormal blood clotting. Moreover, venom of Crotalinae, a subfamily belonging to the Viperidae, exhibits neurotoxicity caused by the blockage of calcium channels [52]. From a pharmaceutical point of view, snake venom is an inexhaustible wealth of both new drugs and diagnostic agents. Viperidae venom has mostly hemotoxic properties, its ingredients are used for treating various thrombo-embolic disorders by targeting coagulation, fibrinolysis, or platelet functions and also in diagnosis of function and dysfunction of hemostatic system elements [36]. Getting to know the composition of the venom’s previously undescribed species and the exact characteristics of already known components opens up new possibilities for the treatment of many diseases, as well as the development of effective serums and treatment of bites.

## 4. Materials and Methods

Venom of *Agkistrodo contortrix contortrix* was extracted in the breeding garden Pata near Hlohovec (Slovakia), which had been designed for reptiles conservation of the gene pool under the veterinary certificate No. CHEZ-TT-01. The breeding garden also serves as a quarantine station for imported animals and is an official importer of exotic animals from around the world, having the permission of the State Nature Protection of the Slovak Republic under the No. 03418/06, the trade with endangered species of wild fauna and flora and on amendments to certain laws under Law No. 237/2002 Z.z.

The selected specimens (two females and one male) were caught in a standard way. Special gloves and special hooks were used as well as the rubber pinch fixators fixing the head of the venomous snake as the hand safely grasped the snake closely just behind the head. Venom was extracted directly into eppendorf tubes or micropipettes and stored at −20 °C (transport temperature) then stored in a deep freezer at −80 °C for deep freezing.

The detailed procedure for proteomic and peptidomic analysis was described in our previous work [20]. Peptides under MW 3 kDa were separated with the use of centrifugal filters in accordance with the manufacturer’s instructions (VWR 82031-344). Protein concentration was determined using the 2-D Quant Kit (GE Healthcare, Little Chalfont, UK) with bovine serum albumin as a standard. Aliquots of 405 μg proteins were mixed with standard thiourea rehydration solutions containing IPG buffers (GE Healthcare, Little Chalfont, UK) range pH range 3–10 and 5–8, respectively. Rehydratation and isoelectrofocusing were performed on 17 cm ReadyStrip IPG Strips with pH ranges 5–8 and 3–10 (Bio-Rad, Hercules, CA, USA), the second dimension under reducing and denaturing condition (SDS-PAGE) was performed using 13% polyacrylamide gels (1.5 × 255 × 196 mm) with Roti^®^-Mark PRESTAINED molecular weight marker (Roth, Karlsruhe, Germany) as a standard. Following electrophoresis the gels were stained with colloidal Coomassie Brilliant Blue G-250. Percentage of proteins from different groups has been estimated in Image Master 2D Platinum software (GE Healthcare, Little Chalfont, UK) using %Vol (a ratio of the volume of a particular spot to the total volume of all spots present in the gel). The final result is an average of the spots %Vol determined from all gels (three technical repeats and two pH ranges).

All the spots present on the gels were excised from gels and digested using Sequencing Grade Modified Trypsin (Promega, Madison, WI, USA). Peptides derived from the filtration and those obtained from the proteins digestion were mixed with the matrix α-Cyano-4-hydroxycinnamic acid in 1:1 ratio.

Peptide masses were measured using a MALDI-ToF/ToF MS (Autoflex Speed, Bruker Daltonics, Billerica, MA, USA) with analyzer working in the reflective mode and positive ions were recorded in the mass range between 700 and 3500 Da. Mass calibration was performed after every four samples using standards in the range of analytes (Peptide Calibration Standards I, Bruker Daltonics, Billerica, MA, USA). The obtained peptide mass fingerprint data were exported to the Mascot software for MSDB (Model System Database) or SwissProt database search (www.matrixscience.com). The following search parameters were applied: mass tolerance was set to 0.2 Da, one incomplete cleavage was allowed, alkylation of cysteine by carbamidomethylation as fixed, and oxidation of methionine as variable modification. Particular peptides selected from mass spectrum were sequenced by laser-induced dissociation (LID) using LIFT ion source and tandem mass spectrum were analyzed as described above. The search parameters for MS/MS data were the same as those applied for MALDI-ToF analyses with one exception: mass tolerance was set to 0.4 Da for MS mode and 0.2 Da for MS/MS mode. For peptidome analysis no fixed modifications were marked but additional variable modifications have been selected instead: *N*-terminal glutamate to pyroglutamate conversion and deamidation on asparagine.

## Figures and Tables

**Table 1:** Proteins identified in *A. c. contortrix* venom.

Spot no. ^#^	Identified Protein ^&^	Accession *	Organism ^¥^	Mass [kDa] ^£^	S ^±^	Peptide Sequence ^¤^
1	Snake venom 5′-nucleotidase	V5NTD_CROAD	*Crotalus adamanteus*	65.2	97	ETPVLSNPEGPYLEFR (1719.099)
		V5NTD_CROAD	*Crotalus adamanteus*	65.2	39	QAFEHSVHR (1110.651)
		V5NTD_GLOBB	*Gloydius blomhoffii blomhoffii*	6	115	SFELTILHTNDVMAR (1753.091)
		V5NTD_GLOBR	*Gloydius brevicaudus*	65	65	PMF SC 13.3%
2	l-amino-acid oxidase	OXLA_GLOHA	*Gloydius halys*	57.4	116	ETDYEEFLEIAR (1514.762)
		OXLA_PSEAU	*Pseudechis austarlis*	59	54	PMS SC 10.4%
3	Beta-fibrinogenase brevinase	VSPB_GLOBL	*Gloydius blomhoffii*	26.3	65	VIGGDECNINEHR (1512.767)
	Beta-fibrinogenase	VSPBF_MACLB	*Macrovipera lebetina*	28.2	26	FFCLSSK (888.452)
4	Thrombin-like enzyme bilineobin	VSP2_AGKBI	*Agkistrodon bilineatus*	27.1	64	IIGGDECNINEHR (1526.785)
					28	NSEHIAPLSLPSSPPIVGSVCR (2317.179)
	Thrombin-like enzyme asperase	VSPL_BOTAS	*Bothrops asper*	28.6	118	ETYPDVPHCANINILDHAVCR (2494.238)
	Snake venom serine protease PA	VSPP_TRIST	*Trimeresurus stajnegri*	28.6	40	VVLNEDEQIR (1115.567)
	Snake venom serine proteinase pallabin	VSP1_GLOHA	*Gloydius halys*	29.3	27	LDSPVKNSAHIAPLSLPSSPPVGSDCR (2888.600)
	Snake venom serine proteinase 9	VSP9_CROAD	*Crotalus adamanteus*	11.7	164	ETYPDVPHCANINILDYEVCR (2578.230)
	Snake venom serine proteinase 12	VSPC_CROAD	*Crotalus adamanteus*	29.3	113	DIMLIRLDSPVSNSEHIAPLSLPSSPPSVGSVCR (1823.985)
	Thrombin-like enzyme crotalase	VSPCR_CROAD	*Crotalus adamanteus*	30.1	36	WDKDIMLIR(1189.656)
5	Protein C activator	VSPCA_AGKCO	*Agkistrodon contortrix contortrix*	25.7	82	PMF SC 34,2%
					91	NSAHIAPLSLPSNPPSVGSVCR (2260.075)
		VSPCA_AGKBI	*Agkistrodon bilineatus*	2.2	68	VVGGDECNINEHR (1498.711)
6	Basic phospholipase A2 homolog	PA2HB_AGKPI	*Agkistrodon piscivorus piscivorus*	14.7	80	PMF SC 49%
	Basic phospholipase A2 homolog MT1	PA2H1_AGKCL	*Agkistrodon contrortrix laticinctus*	16.5	80	PMF SC 43%
7	Cysteine-rich venom protein piscivorin	CRVP_AGKPI	*Agkistrodon piscivorus piscivorus*	27.5	102	MEHYPEAAANAER (1537.689)
					76	MEHYPEAAANAER (1553.669)
8	Snake venom metalloproteinase ACLF	VM1A_AGKCL	*Agkistrodon piscivorus loucostroma*	47.1	67	YVELVIIADHR (1327.857)
					29	SHDNAQLLATAIVFDGIIGR (2169.126)
		VM1A_AGKCL	*Agkistrodon contortrix latiematus*	26.7	77	YVELVIVADHR (1313.725 )
	Zinc metalloproteinase disintegrin	VM2AB_AGKCO	*Agkistordon contortrix contortrix*	55.1	185	ISHDNAQLLTAIELDGETIGLANR (2564.272 )
					41	YIELVVVADHR (1313, 713)
	Snake venom metalloproteinase VMP1	VM1V1_AGKPL	*Agkistrodon piscivorus leucostoma*	47.1	136	SHDNAQLLTAIVFDEGIIGR (2169.058)
					37	APLAGMCDPNR (1201.591)
					43	YVELVIVADHR (1327.784)
	Zinc metalloproteinase disintegrin-like HR1a	VM3HA_PROFL	*Protobothrops flavoviridis*	70.9	59	TWVYEIVNTLNEIYR (1912.993)
	Snake venom metalloproteinase fibrolase	VM1F_AGKCO	*Akgistrodon contortrix contortrix*	23.2	42	YVQLVIVADHR (1312.613)
9	Acidic phospholipase A2 BpirPLA2-I	PA2A1_BOTPI	*Bothrops pirajai*	14.4	48	CCFVMDCCYGK (1505.585)
					49	QICECDR (980.433)
	Acidic phospholipase A2 S1E6-b	PA2AB_CALRH	*Calloselasma rhodostoma*	14.3	56	PMF SC 30.2%
	Acidic phospholipase A2	PA2A_GLOHA	*Gloydius halys*	14.7	57	PMF SC 25.8 %
	Acidic phospholipase A2 1	PA2A1_PROFL	*Protobothrops flavoviridis*	15.5	30	AAAICFR (808.334)
10	C-type lectin APL	LECG_AGKPI	*Agkistrodon piscivorus piscivorus*	16.7	103	PMF SC 51.1%
					54	DFSWEWTDR (1241.611)
					105	EFCVELVSLTGYR (1572.785)
					101	GQAEVWIGLWDK (1401.639)
	C- type lectin PAL	LECG_BITAR	*Bitis arietans*	16.6	56	PMF SC 58.5%

^#^ Spot numbering was the same as in Figure 1 and Figure 2; ^&^ Protein name in database; * Database accession number of homologous proteins; ^¥^ Organism from which protein identification originates; ^£^ The mass of molecule; ± Protein identification was performed using the Mascot search with probability based Mowse score. Ions score was −10 × log(P), where P was the probability that the observed match was a random event. Mascot defined thresholds which indicated identity or extensive homology (*p* < 0.05) was 26; **^¤^** Peptide sequence derived from LIFT analysis. Identification of proteins by MS/MS method was conducted by comparing obtained sequences with sequences from database. In brackets: mass of precursor ion. In the case of PMF identification SC—amino acid sequence coverage for the identified proteins. In the PMF identification case the highest score and SC shown. Representative MS and MS/MS spectra used for the protein identification are included as supplementary material.

**Table 2:** Peptides identified in *A. contortrix contortrix* venom.

Parent ion *m/z*	Identified Protein ^&^	Accession *	Organism ^¥^	Peptide Sequence ^≠^	Mass [Da] ^£^	S ^±^
1063.5343	Bradykinin inhibitor peptide	BKIP_AGKBI	*Agkistrodon bilineatus*	TPPAGPDVGPR	1063	9
1214.6465	Bradykinin-potentiating peptide POL-236	BPP36_CROAT	*Crotalus atrox*	QLWPRPQIPP- + Gln- > pyro-Glu (N-term Q)	1231	59
1230.6420	Bradykinin potentiating peptide E	gi|229310	*Gloydius blomhoffii*	EKWDPPPVSPP- + Glu- > pyro-Glu (N-term E)	1248	13

^&^ Peptide name in database; * Database accession number of homologous peptide; ^¥^ Organism from which peptide identification originates; ^£^ The mass of molecule; ^≠^ Peptide sequence derived from LIFT analysis; ^±^ Peptide identification was performed using the Mascot search with probability based Mowse score. Ions score was −10 × log(*P*), where *P* was the probability that the observed match was a random event.

## Supplementary Materials

The following are available online at www.mdpi.com/2072-6651/8/12/372/s1, Figures S4, S19, S20, S35, S36, S40, S41: Annotated MS spectra; Figures S1-S3, S5-S18, S21-S34, S37-S39: Annotated MS/MS spectra for different ions.
