# Supplementary material for: Proteomic Analyses of Agkistrodon contortrix contortrix Venom Using 2D Electrophoresis and MS Techniques
Source: Toxins (Basel). 2016 Dec 13;8(12):372. doi: 10.3390/toxins8120372 (PMC5198566; doi:10.3390/toxins8120372)
Supplement: Supplementary file 1 [file toxins-08-00372-s001.pdf]

Supplementary Materials: Proteomic Analyses of *Agkistrodon contortrix contortrix* Venom Using 2D Electrophoresis and MS Techniques

Aleksandra Bocian, Małgorzata Urbanik, Konrad Hus, Andrzej Łyskowski, Vladimír Petrilla, Zuzana Andrejčáková, Monika Petrillová and Jaroslav Legath

Selected MS and MS/MS annotated spectra used for protein and peptide identification.

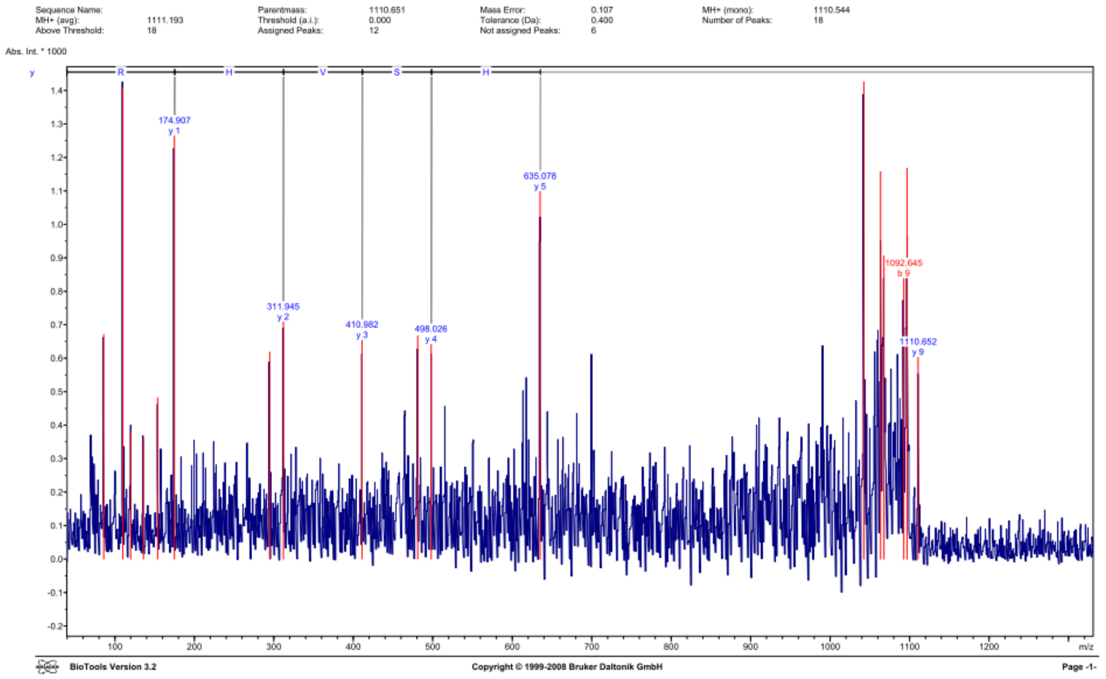

(A)

Display Parameter:

Parentmass:	1110.651	Mass Error:	0.107	MH+ (mono):	1110.544	MH+ (avg):	1111.193
Threshold (a.i.):	0.000	Tolerance (Da):	0.400	Number of Peaks:	18	Above Threshold:	18
Assigned Peaks:	12	Not assigned Peaks:	6				

Peaklist:

Peak	Mass	Intensity	Peak	Mass	Intensity	Peak	Mass	Intensity	Peak	Mass	Intensity	Peak	Mass	Intensity
1	85.931	671.133	2	109.895	1408.531	3	119.864	380.260	4	135.884	361.627	5	153.898	482.150
6	174.907	1265.034	7	294.913	618.522	8	311.945	708.918	9	410.982	654.804	10	481.006	666.813
11	498.026	642.009	12	635.078	1098.612	13	1042.293	1501.746	14	1063.367	1157.338	15	1067.793	906.116
16	1092.645	838.758	17	1096.728	1167.544	18	1110.652	603.743						

Calculated Masses:
QAFEHSVHR

N-Term.	Ion	a	a-17	a-18	b	b-17	b-18	b+18	c	i	x	y	z	C-Term.	Ion
1	Q	101.071	84.044	83.060	129.066	112.039	111.055	147.076	146.092	101.071	201.098	**175.119**	158.092	9	R
2	A	172.108	155.082	**154.097**	200.103	183.076	182.092	218.114	217.130	44.049	338.157	**312.178**	**295.151**	8	H
3	F	319.176	302.150	301.166	347.171	330.145	329.161	365.182	364.198	**120.081**	437.226	**411.246**	394.220	7	V
4	E	448.219	431.193	430.208	476.214	459.187	458.203	494.225	493.241	102.055	524.258	**498.278**	**481.252**	6	S
5	H	585.278	568.251	567.267	613.273	596.246	595.262	631.283	630.299	**110.071**	661.316	**635.337**	618.311	5	H
6	S	672.310	655.283	654.299	700.305	683.278	682.294	718.315	717.331	60.044	790.359	764.380	747.353	4	E
7	V	771.378	754.352	753.368	799.373	782.347	781.363	817.384	816.400	72.081	937.427	911.448	894.422	3	F
8	H	908.437	891.411	890.427	936.432	919.406	918.422	954.443	953.459	**110.071**	1008.465	982.485	965.459	2	A
9	R	1064.538	1047.512	1046.528	**1092.533**	1075.507	1074.523	**1110.544**	1109.560	129.113	1136.523	**1110.544**	1093.517	1	Q

BioTools Version 3.2 Copyright © 1999-2008 Bruker Daltonik GmbH Page -2-

(B)

Figure S1. Annotated MS/MS spectrum of ion 1110.651 *m*/*z* detected in area #1. (**A**) MS/MS spectrum; (**B**) Peak list and amino acid sequence.

Sequence Name:		Parentmass:	1719.099	Mass Error:	0.223	MH+ (mono):	1718.875
MH+ (avg):	1719.915	Threshold (a.i.):	0.000	Tolerance (Da):	0.700	Number of Peaks:	57
Above Threshold:	57	Assigned Peaks:	31	Not assigned Peaks:	26		

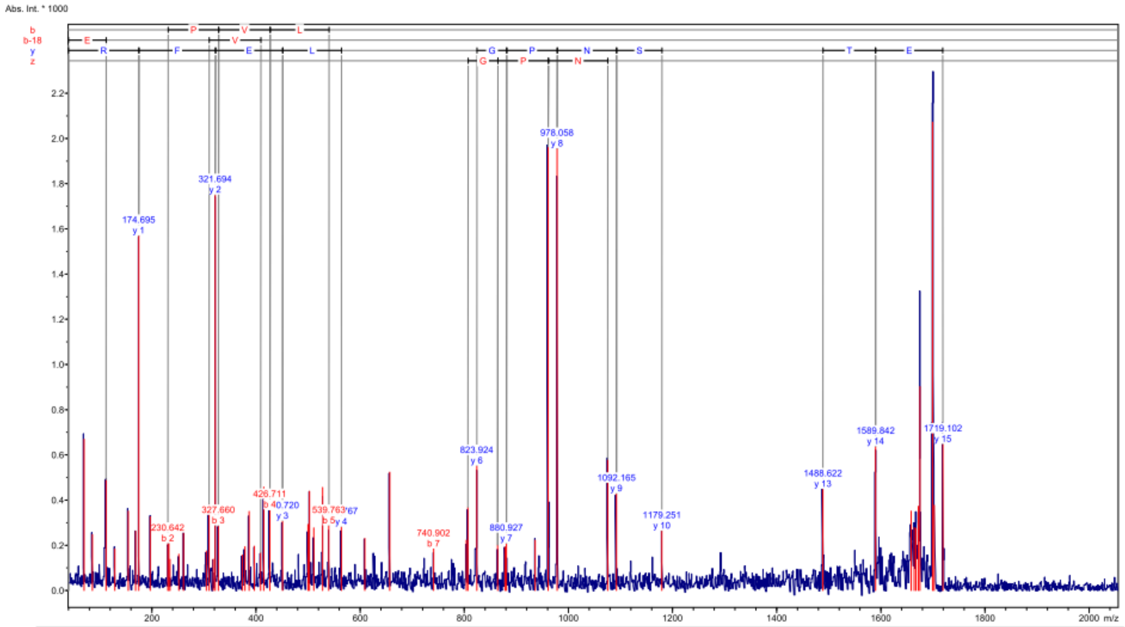

BioTools Version 3.2 Copyright © 1999-2008 Bruker Daltonik GmbH Page -1-

(**A**)

Display Parameter:

Parentmass:	1719.099	Mass Error:	0.223	MH+ (mono):	1718.875	MH+ (avg):	1719.915
Threshold (a.i.):	0.000	Tolerance (Da):	0.700	Number of Peaks:	57	Above Threshold:	57
Assigned Peaks:	31	Not assigned Peaks:	26				

Peaklist:

Peak	Mass	Intensity	Peak	Mass	Intensity	Peak	Mass	Intensity	Peak	Mass	Intensity	Peak	Mass	Intensity
1	69.765	669.717	2	85.752	247.859	3	111.729	489.472	4	128.737	184.337	5	154.679	351.681
6	168.738	262.876	7	174.695	1570.659	8	196.701	326.898	9	230.642	208.711	10	232.668	228.652
11	234.620	138.161	12	251.678	161.135	13	260.649	255.508	14	304.680	174.751	15	309.689	382.696
16	317.635	138.609	17	321.694	1751.162	18	327.660	288.016	19	373.711	157.317	20	378.726	194.354
21	386.704	349.862	22	396.732	196.104	23	408.713	167.135	24	414.694	457.456	25	426.711	358.421
26	450.720	308.492	27	499.766	292.526	28	502.731	439.039	29	510.728	276.864	30	527.751	456.024
31	539.763	288.031	32	563.767	282.616	33	608.795	231.049	34	656.798	523.770	35	740.902	185.291
36	803.885	222.696	37	806.896	367.889	38	823.924	552.701	39	863.920	201.320	40	877.902	192.451
41	880.927	208.691	42	935.991	222.741	43	961.021	1963.224	44	978.058	1956.384	45	1075.117	578.885
46	1092.165	429.696	47	1179.251	264.981	48	1488.622	449.786	49	1589.842	638.157	50	1658.138	351.306
51	1663.882	278.621	52	1667.335	275.526	53	1671.712	372.741	54	1675.135	902.221	55	1699.293	2072.240
56	1702.362	377.086	57	1719.102	648.155									

Calculated Masses:
ETPVLSNPGPYLEFR

N-Term.	Ion	a	a-17	a-18	b	b-17	b-18	b+18	c	i	x	y	z	C-Term.	Ion
1	E	102.055	85.028	84.044	130.050	113.023	**112.039**	148.060	147.076	102.055	201.098	**175.119**	158.092	15	R
2	T	203.103	186.076	185.092	**231.098**	214.071	213.087	249.108	248.124	74.060	348.167	**322.187**	**305.161**	14	F
3	P	300.155	283.129	282.145	**328.150**	311.124	**310.140**	346.161	345.177	**70.065**	477.209	**451.230**	434.203	13	E
4	V	399.224	382.197	381.213	**427.219**	410.192	**409.208**	445.229	444.245	72.081	590.293	**564.314**	547.287	12	L
5	L	512.308	495.281	494.297	**540.303**	523.276	522.292	558.313	557.329	**86.096**	753.357	727.377	710.351	11	Y
6	S	599.340	582.313	581.329	627.335	610.308	**609.324**	645.345	644.361	60.044	850.409	**824.430**	**807.404**	10	P
7	N	713.383	696.356	695.372	**741.378**	724.351	723.367	759.388	758.404	87.055	907.431	**881.452**	**864.425**	9	G
8	P	810.436	793.409	792.425	838.431	821.404	820.420	856.441	855.457	**70.065**	1004.484	**978.504**	**961.478**	8	P
9	G	867.457	850.431	849.446	895.452	**878.425**	**877.441**	913.463	912.479	30.034	1118.527	**1092.547**	**1075.521**	7	N
10	P	964.510	947.483	946.499	992.505	975.478	974.494	1010.515	1009.531	**70.065**	1205.559	**1179.579**	1162.553	6	S
11	Y	1127.573	1110.547	1109.563	1155.568	1138.542	1137.557	1173.579	1172.595	136.076	1318.643	1292.663	1275.637	5	L
12	L	1240.657	1223.631	1222.647	1268.652	1251.626	1250.642	1286.663	1285.679	**86.096**	1417.711	1391.732	1374.705	4	V
13	E	1369.700	1352.673	1351.689	1397.695	1380.668	1379.684	1415.705	1414.721	102.055	1514.764	**1488.785**	1471.758	3	P
14	F	1516.768	1499.742	1498.758	1544.763	1527.737	1526.753	1562.774	1561.790	120.081	1615.811	**1589.832**	1572.806	2	T
15	R	1672.869	1655.843	1654.859	1700.864	1683.838	1682.854	**1718.875**	1717.891	**129.113**	1744.854	**1718.875**	**1701.848**	1	E

BioTools Version 3.2 Copyright © 1999-2008 Bruker Daltonik GmbH Page -2-

(**B**)

Figure S2. Annotated MS/MS spectrum of ion 1719.099 *m*/*z* detected in area #1. (**A**) MS/MS spectrum; (**B**) Peak list and amino acid sequence.

Sequence Name:		Parentmass:	1753.091	Mass Error:	0.188	MH+ (mono):	1752.903
MH+ (avg):	1753.936	Threshold (a.i.):	0.000	Tolerance (Da):	0.700	Number of Peaks:	51
Above Threshold:	51	Assigned Peaks:	33	Not assigned Peaks:	18		

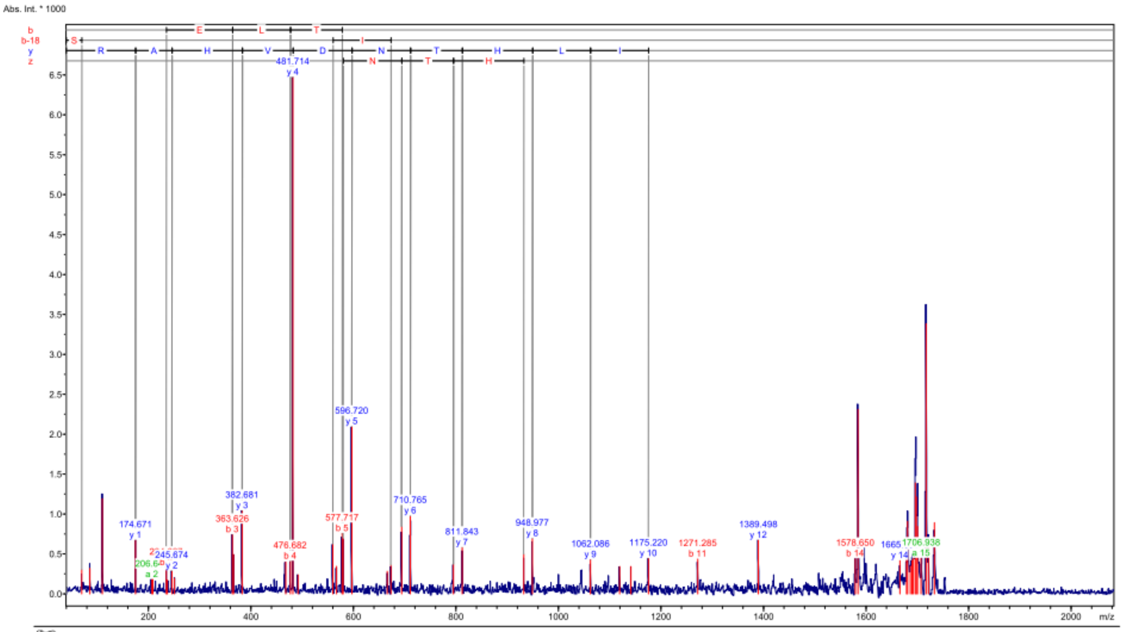

BioTools Version 3.2 Copyright © 1999-2008 Bruker Daltonik GmbH Page -1-

(**A**)

Display Parameter:

Parentmass:	1753.091	Mass Error:	0.188	MH+ (mono):	1752.903	MH+ (avg):	1753.936
Threshold (a.i.):	0.000	Tolerance (Da):	0.700	Number of Peaks:	51	Above Threshold:	51
Assigned Peaks:	33	Not assigned Peaks:	18				

Peaklist:

Peak	Mass	Intensity	Peak	Mass	Intensity	Peak	Mass	Intensity	Peak	Mass	Intensity	Peak	Mass	Intensity
1	69.750	303.442	2	85.728	320.874	3	109.704	1194.728	4	174.671	675.113	5	206.648	183.523
6	208.671	219.308	7	234.627	332.609	8	236.654	175.751	9	245.674	293.376	10	250.663	196.315
11	363.626	746.016	12	365.672	494.541	13	382.681	1045.976	14	467.640	399.995	15	476.682	414.731
16	481.714	6475.628	17	491.723	240.283	18	559.713	624.293	19	566.689	345.385	20	577.717	761.331
21	579.708	694.868	22	596.720	2100.182	23	665.760	283.689	24	672.764	358.865	25	693.766	843.480
26	710.765	980.846	27	794.823	371.885	28	811.843	584.014	29	931.930	496.981	30	948.977	697.156
31	1062.086	432.799	32	1119.110	344.438	33	1141.093	343.229	34	1175.220	447.717	35	1271.285	442.002
36	1389.498	676.671	37	1578.650	444.760	38	1583.907	2313.701	39	1665.690	421.474	40	1678.639	522.613
41	1680.850	908.515	42	1686.069	349.777	43	1689.470	420.338	44	1691.971	449.040	45	1695.870	1392.440
46	1697.894	1214.516	47	1700.147	845.515	48	1706.938	450.152	49	1716.816	3385.501	50	1721.572	595.803
51	1733.024	891.910												

Calculated Masses:
SFELTILHTNDVHAR

N-Term.	Ion	a	a-17	a-18	b	b-17	b-18	b+18	c	i	x	y	z	C-Term.	Ion
1	S	60.044	43.018	42.034	88.039	71.013	**70.029**	106.050	105.066	60.044	201.098	**175.119**	158.092	15	R
2	F	**207.113**	190.086	189.102	**235.108**	218.081	217.097	253.118	252.134	120.081	272.135	**246.156**	229.130	14	A
3	E	336.155	319.129	318.145	**364.150**	347.124	346.140	**382.161**	381.177	102.055	409.194	**383.215**	**366.188**	13	H
4	L	449.239	432.213	431.229	**477.234**	460.208	459.224	495.245	494.261	**86.096**	508.263	**482.283**	465.257	12	V
5	T	550.287	533.261	532.277	**578.282**	561.256	**560.271**	**596.293**	595.309	74.060	623.290	**597.310**	**580.284**	11	D
6	I	663.371	646.345	645.361	691.366	674.340	**673.356**	709.377	708.393	**86.096**	737.333	**711.353**	**694.327**	10	N
7	L	776.455	759.429	758.445	804.450	787.424	786.440	822.461	821.477	**86.096**	838.380	**812.401**	**795.374**	9	T
8	H	913.514	896.488	895.504	941.509	924.483	923.499	959.520	958.536	**110.071**	975.439	**949.460**	**932.433**	8	H
9	T	1014.562	997.535	996.551	1042.557	1025.530	1024.546	1060.567	1059.583	74.060	1088.523	**1062.544**	1045.517	7	L
10	N	1128.605	1111.578	1110.594	1156.600	1139.573	1138.589	**1174.610**	1173.626	87.055	1201.607	**1175.628**	1158.601	6	I
11	D	1243.632	1226.605	1225.621	**1271.627**	1254.600	1253.616	1289.637	1288.653	88.039	1302.655	1276.676	1259.649	5	T
12	V	1342.700	1325.674	1324.690	1370.695	1353.669	1352.684	1388.706	1387.722	72.081	1415.739	**1389.760**	1372.733	4	L
13	H	1479.759	1462.733	1461.748	1507.754	1490.727	1489.743	1525.765	1524.781	**110.071**	1544.782	1518.802	1501.776	3	E
14	A	1550.796	1533.770	1532.786	**1578.791**	1561.765	1560.781	1596.802	1595.818	44.049	**1691.850**	**1665.871**	1648.844	2	F
15	R	**1706.897**	**1689.871**	**1688.887**	1734.892	1717.866	**1716.882**	1752.903	1751.919	129.113	1778.882	1752.903	1735.876	1	S

BioTools Version 3.2 Copyright © 1999-2008 Bruker Daltonik GmbH Page -2-

(**B**)

Figure S3. Annotated MS/MS spectrum of ion 1753.091 *m*/*z* detected in area #1. (**A**) MS/MS spectrum; (**B**) Peak list and amino acid sequence.

Sequence Name:	Snake venom 5'-nucleotidase OS=Gloydius brevicaudus PE=2 SV=1 V5NTD_GLOBR				Formula:		Parentmass:	
Mass Error:		MH+ (mono):	1.008	MH+ (avg): 1.008	Threshold (a.i.):	0.000	Tolerance (Da):	0.500
Number of Peaks:	24	Above Threshold:		Assigned Peaks:	Not assigned Peaks:			

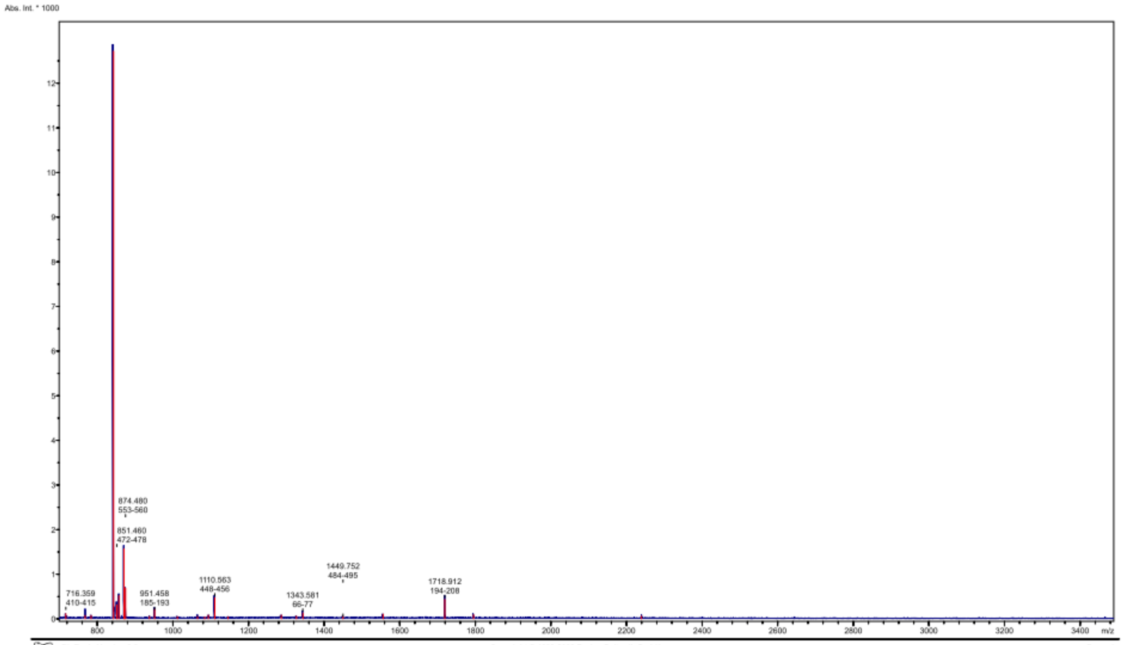

BioTools Version 3.2 Copyright © 1999-2008 Bruker Daltonik GmbH Page -1-

(**A**)

Sequence data:

Snake venom 5'-nucleotidase OS=Gloydius brevicaudus PE=2 SV=1 V5NTD_GLOBR

Intensity Coverage:	13.4 % (2438 cnts)	Sequence Coverage MS:	13.3%
Sequence Coverage MS/MS:	0.0%	pI (isoelectric point):	9.4

10	20	30	40	50	60	70	80	90
MQTPKRRRGA	QGCPRSSPSP	PLLLLVGAVW	FCAALSVAAG	SFELTILHTN	DVHARVEQTS	RDSGKCTGQD	CYGGVARRAT	KIRELRANHS
100	110	120	130	140	150	160	170	180
HVLLLDAGDQ	YQGTIWFSFF	KGREVVKFMN	SLGYDAMALG	NHEFDNGLAG	LLDPLLKHAN	FPILSANIRP	KGSIASNISG	YILPYKIINV
190	200	210	220	230	240	250	260	270
GSEKVGIIGY	TTKETPVLSN	PGPYLEFRDE	VEELQKHANK	LTTLGVNKII	ALGHSGFFED	QRIARKVKGV	DVVVGGHTNT	FLYTGSPPST
280	290	300	310	320	330	340	350	360
EVAAGNYPFM	VKSDDGRQVP	VVQAYAFGKY	LGYLNVIFDD	KGNVIKSSGN	PILLNKNISE	DQDVKAEVNK	MKIQLHNYSS	QEIGKTIVYL
370	380	390	400	410	420	430	440	450
NGTTQACRFM	ECNLGNLICD	AVIYNNVRMP	DYNEWNMVSM	CIVNGGGIRS	PIDERANNGT	ITLEELTAVL	PFGGTFDLLQ	IKGCALKQAF
460	470	480	490	500	510	520	530	540
EHSVHRHGQG	MGELLQVSGI	KVVYDLSRKP	GSRVVSLNVL	CTECRVPTYV	PLEKEKTYKL	LLPSFLAGGG	DGYHMLKGDS	SNHSSGNLDI
550	560	570	580	590				
SIVGDYIKRM	GKVFPAVEGR	VIFSAGTLFQ	AQLFLTWGLC	ISLLYFIL				

Display Parameter:

Sequence Name:	Snake venom 5'-nucleotidase OS=Gloydius brevicaudus PE=2 SV=1 V5NTD_GLOBR			MH+ (mono):	1.008	MH+ (avg):	1.008
Threshold (a.i.):	0.000	Tolerance (Da):	0.500	Number of Peaks:	24		

Peaklist:

Peak	Mass	Intensity	Peak	Mass	Intensity	Peak	Mass	Intensity	Peak	Mass	Intensity	Peak	Mass	Intensity	Peak	Mass	Intensity
1	716.359	119.000	2	767.333	78.000	3	783.386	75.000	4	842.506	12715.453	5	851.460	315.866	6	856.523	522.473
7	870.543	1570.611	8	874.480	691.314	9	937.422	55.000	10	951.458	216.281	11	1011.650	51.000	12	1064.580	54.000
13	1083.534	42.000	14	1093.542	84.000	15	1110.563	472.851	16	1145.589	46.000	17	1286.621	85.000	18	1326.556	62.000
19	1343.581	131.571	20	1449.752	54.000	21	1555.839	105.000	22	1718.912	437.243	23	1794.863	91.472	24	2239.187	61.000

BioTools Version 3.2 Copyright © 1999-2008 Bruker Daltonik GmbH Page -2-

(**B**)

Figure S4. Annotated MS spectrum detected in area #1. (**A**) MS spectrum; (**B**) Peak list and sequence data.

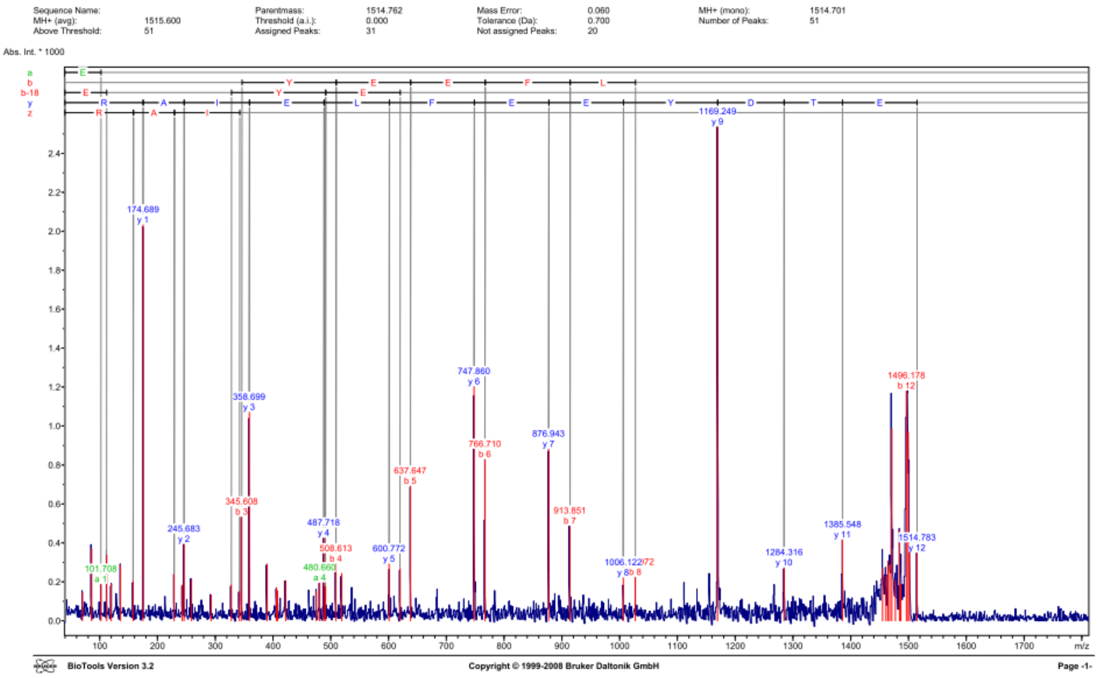

(**A**)

Display Parameter:

Parentmass: 1514.762 Mass Error: 0.060 MH+ (mono): 1514.701 MH+ (avg): 1515.600
Threshold (a.i.): 0.000 Tolerance (Da): 0.700 Number of Peaks: 51 Above Threshold: 51
Assigned Peaks: 31 Not assigned Peaks: 20

Peaklist:

Peak	Mass	Intensity	Peak	Mass	Intensity	Peak	Mass	Intensity	Peak	Mass	Intensity	Peak	Mass	Intensity
1	69.758	144.342	2	85.785	370.602	3	101.708	188.694	4	111.735	338.748	5	119.703	188.962
6	135.687	286.051	7	157.663	206.026	8	174.689	2034.276	9	228.668	241.341	10	242.675	183.162
11	245.683	394.237	12	258.634	211.591	13	292.632	132.562	14	327.606	186.866	15	341.647	153.477
16	345.608	534.592	17	358.699	1071.347	18	389.690	292.154	19	405.622	171.036	20	407.626	154.817
21	421.591	206.166	22	474.613	152.614	23	480.660	195.808	24	487.718	425.958	25	490.618	177.852
26	508.613	293.187	27	518.658	243.713	28	600.772	292.860	29	619.656	270.396	30	637.647	692.782
31	747.860	1203.182	32	766.710	830.132	33	876.943	882.512	34	913.851	487.623	35	1006.122	221.589
36	1026.972	224.336	37	1169.249	2537.872	38	1284.316	273.534	39	1385.548	417.008	40	1454.804	269.168
41	1460.778	231.452	42	1464.070	308.232	43	1467.354	282.247	44	1470.776	986.059	45	1477.052	207.897
46	1484.218	466.328	47	1486.673	187.193	48	1496.178	1182.011	49	1499.215	969.786	50	1501.447	354.050
51	1514.783	349.679												

Calculated Masses:
ETDYEEFLEIAR

N-Term.	Ion	a	a-17	a-18	b	b-17	b-18	b+18	c	i	x	y	z	C-Term.	Ion
1	E	**102.055**	85.028	84.044	130.050	113.023	**112.039**	148.060	147.076	**102.055**	201.098	**175.119**	**158.092**	12	R
2	T	203.103	186.076	185.092	231.098	214.071	213.087	249.108	248.124	74.060	272.135	**246.156**	**229.130**	11	A
3	D	318.130	301.103	300.119	**346.124**	329.098	**328.114**	364.135	363.151	88.039	385.219	**359.240**	**342.214**	10	I
4	Y	**481.193**	464.166	463.182	**509.188**	492.161	**491.177**	527.198	526.214	**136.076**	514.262	**488.283**	471.256	9	E
5	E	610.235	593.209	592.225	**638.230**	621.204	**620.220**	656.241	655.257	**102.055**	627.346	**601.367**	584.340	8	L
6	E	739.278	722.252	721.268	**767.273**	750.246	749.262	785.284	784.300	**102.055**	774.414	**748.435**	731.409	7	F
7	F	886.347	869.320	868.336	**914.341**	897.315	896.331	932.352	931.368	**120.081**	903.457	**877.478**	860.451	6	E
8	L	999.431	982.404	981.420	**1027.425**	1010.399	1009.415	1045.436	1044.452	**86.096**	1032.500	**1006.520**	989.494	5	E
9	E	1128.473	1111.447	1110.463	1156.468	1139.442	1138.458	1174.479	1173.495	**102.055**	1195.563	**1169.584**	1152.557	4	Y
10	I	1241.557	1224.531	1223.547	1269.552	1252.526	1251.542	1287.563	1286.579	**86.096**	1310.590	**1284.611**	1267.584	3	D
11	A	1312.594	1295.568	1294.584	1340.589	1323.563	1322.579	1358.600	1357.616	44.049	1411.638	**1385.658**	1368.632	2	T
12	R	1468.695	1451.669	1450.685	**1496.690**	1479.664	1478.680	**1514.701**	1513.717	129.113	1540.680	**1514.701**	1497.674	1	E

BioTools Version 3.2 Copyright © 1999-2008 Bruker Daltonik GmbH Page -2-

(**B**)

Figure S5. Annotated MS/MS spectrum of ion 1514.762 *m*/*z* detected in area #2. (**A**) MS/MS spectrum. (**B**) Peak list and amino acid sequence.

Sequence Name: L-amino-acid oxidase OS=Pseudechis australis PE=1 SV=1 OXLA_PSEAU Parentmass:
Mass Error: MH+ (mono): 1.008 MH+ (avg): 1.008 Threshold (a.i.): 0.000
Tolerance (Da): 0.500 Number of Peaks: 16 Above Threshold: Assigned Peaks:
Not assigned Peaks:

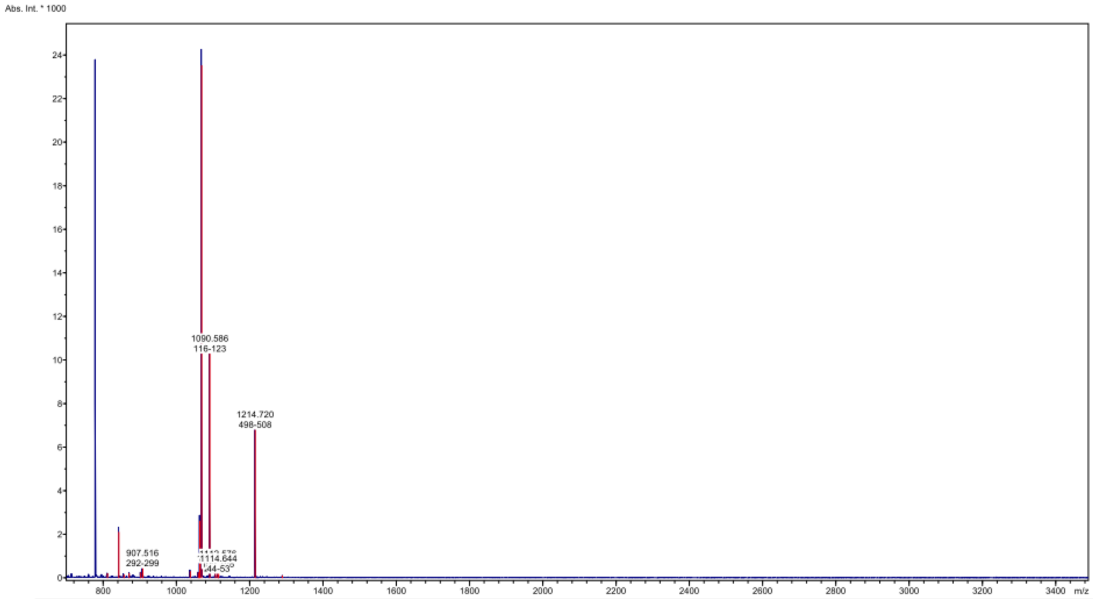

BioTools Version 3.2 Copyright © 1999-2008 Bruker Daltonik GmbH Page -1-

(**A**)

Sequence data:

L-amino-acid oxidase OS=Pseudechis australis PE=1 SV=1 OXLA_PSEAU

Intensity Coverage: 37.9 % (18125 cnts) Sequence Coverage MS: 10.4%
Sequence Coverage MS/MS: 0.0% pI (isoelectric point): 6.3

10	20	30	40	50	60	70	80	90	100	110	120	130	140
MNVFFMFSLL	FLAALGSCAD	DRRRPLEECF	READYEEFLE	IAKNGLQRTS	NPKRVVVVGA	GMAGLSAAYV	LAGAGHQVTL	LEASERVGGR	VNTYRNEKDG	WYVNLGPMRL	PERHRIIREY	IRKFGLELNE	FIQENDNAWY
150	160	170	180	190	200	210	220	230	240	250	260	270	280
FIKNIRKRVS	EVKKDPGVFK	YPVKPSEEGK	SASQLYRESL	QKVIEELKRT	NCSYILNKYD	TYSTKEYLIK	EGNLSPGAVD	MIGDLLNEDS	SYYLSFIESL	KSDDIFSYEK	RFDEIVGGFD	QLPRSMYQAI	AEKVHLNAQV
290	300	310	320	330	340	350	360	370	380	390	400	410	420
IKIQQNAEDV	RVTYQTPAKT	LSYVIADYVI	VCSTSRAARR	IHFEPPLPPK	KAHALRSIHY	RSSTKIFLTC	SQKFWEADGI	HGGKSTTDLP	SRFIYYPNHS	FTSGIGVIVA	YTLADDTDFF	QALDIETSAD	IVINDLSLIH
430	440	450	460	470	480	490	500	510	520				
QLPKEQIQAL	CYPSKIQKWS	LDEYAMGAIT	SFTPYQFQDF	FEIVAAPVGR	IYFAGEYTAS	VHGWLDSTIK	SGLTAARDVN	LASQKPSRIQ	LSNDNEL				

Display Parameter:

Sequence Name: L-amino-acid oxidase OS=Pseudechis australis PE=1 SV=1 OXLA_PSEAU MH+ (mono): 1.008
MH+ (avg): 1.008 Threshold (a.i.): 0.000 Tolerance (Da): 0.500 Number of Peaks: 16

Peaklist:

Peak	Mass	Intensity	Peak	Mass	Intensity	Peak	Mass	Intensity	Peak	Mass	Intensity	Peak	Mass	Intensity
1	811.451	188.457	2	842.537	2093.342	3	856.554	126.476	4	870.576	195.672	5	902.434	229.360
6	907.516	434.994	7	1037.568	289.684	8	1059.552	235.579	9	1063.592	2602.596	10	1068.604	23506.714
11	1090.586	10291.882	12	1106.586	202.855	13	1112.576	400.537	14	1114.644	189.146	15	1214.720	6794.976
16	1288.657	85.256												

BioTools Version 3.2 Copyright © 1999-2008 Bruker Daltonik GmbH Page -2-

(**B**)

Figure S6. Annotated MS spectrum detected in area #2. (**A**) MS spectrum; (**B**) Peak list and sequence data.

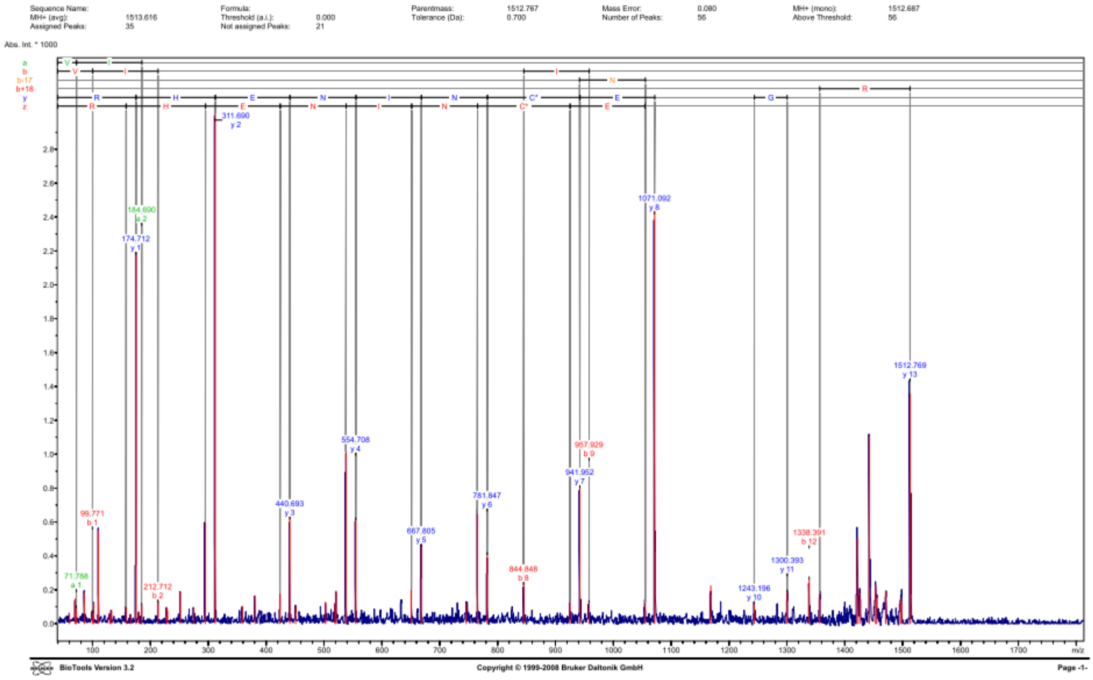

(**A**)

Display Parameter:

Parentmass: 1512.767
Tolerance (Da): 0.700
Mass Error: 0.080
Number of Peaks: 56
MH+ (mono): 1512.687
Above Threshold: 56
MH+ (avg): 1513.616
Assigned Peaks: 35
Threshold (a.i.): 0.000
Not assigned Peaks: 21

Peaklist:

Peak	Mass	Intensity	Peak	Mass	Intensity	Peak	Mass	Intensity	Peak	Mass	Intensity	Peak	Mass	Intensity	Peak	Mass	Intensity
1	59.785	59.866	2	69.783	146.677	3	71.788	90.262	4	85.777	179.973	5	99.771	63.077	6	101.735	120.021
7	109.734	549.747	8	129.723	61.483	9	132.642	77.964	10	157.713	102.051	11	165.634	54.745	12	174.712	2193.195
13	179.671	69.556	14	184.690	104.365	15	212.712	127.397	16	227.686	95.442	17	229.620	75.031	18	251.701	187.788
19	274.591	92.034	20	294.679	599.619	21	311.690	3006.932	22	358.625	102.266	23	380.664	164.671	24	423.697	179.450
25	440.693	630.411	26	450.671	104.638	27	502.662	127.268	28	518.639	114.555	29	520.655	192.240	30	537.699	1016.757
31	554.708	610.024	32	650.796	205.229	33	667.805	469.188	34	745.689	121.403	35	764.833	650.662	36	781.847	404.653
37	844.848	244.218	38	924.868	126.175	39	941.952	815.079	40	957.929	121.211	41	1056.050	124.104	42	1071.092	2432.618
43	1168.134	223.417	44	1243.196	130.734	45	1300.393	183.621	46	1338.391	261.180	47	1356.501	175.919	48	1421.546	504.390
49	1425.687	176.507	50	1442.172	1108.926	51	1452.884	222.727	52	1454.967	146.701	53	1470.235	188.963	54	1495.997	145.320
55	1498.087	123.096	56	1512.769	1360.078												

Calculated Masses:

VIGGDECNINEHR 7: Carbamidomethyl (C)

N-Term.	Ion	a	a-17	a-18	b	b-17	b-18	b+18	c	i	x	y	z	C-Term.	Ion
1	V	**72.081**	55.054	54.070	**100.076**	83.049	82.065	118.086	117.102	**72.081**	201.098	**175.119**	**158.092**	13	R
2	I	**185.165**	168.138	167.154	**213.160**	196.133	195.149	231.170	**230.186**	**86.096**	338.157	**312.178**	**295.151**	12	H
3	G	242.186	225.160	224.176	270.181	253.155	**252.171**	288.192	287.208	30.034	467.200	**441.220**	**424.194**	11	E
4	G	299.208	282.181	281.197	327.203	310.176	309.192	345.213	344.229	30.034	581.243	**555.263**	**538.237**	10	N
5	D	414.235	397.208	396.224	442.230	425.203	**424.219**	460.240	459.256	88.039	694.327	**668.347**	**651.321**	9	I
6	E	543.277	526.251	525.267	571.272	**554.246**	553.262	589.283	588.299	**102.055**	808.370	**782.390**	**765.364**	8	N
7	C*	703.308	686.281	685.297	731.303	714.276	713.292	749.313	748.329	**133.043**	968.400	**942.421**	**925.394**	7	C*
8	N	817.351	800.324	799.340	**845.346**	828.319	827.335	863.358	862.372	87.055	1097.443	**1071.464**	**1054.437**	6	E
9	I	930.435	913.408	912.424	**958.430**	**941.403**	940.419	976.440	975.456	**86.096**	1212.470	1186.491	1169.464	5	D
10	N	1044.478	1027.451	1026.467	1072.473	**1055.446**	**1054.462**	1090.483	1089.499	87.055	1269.491	**1243.512**	1226.485	4	G
11	E	1173.520	1156.494	1155.510	1201.515	1184.489	1183.505	1219.526	1218.542	**102.055**	1326.513	**1300.533**	1283.507	3	G
12	H	1310.579	1293.553	1292.569	**1338.574**	1321.548	1320.564	**1356.585**	1355.601	**110.071**	1439.597	1413.618	1396.591	2	I
13	R	1466.680	1449.654	1448.670	1494.675	1477.649	1476.665	**1512.686**	1511.702	**129.113**	1538.665	**1512.686**	**1495.659**	1	V

BioTools Version 3.2 Copyright © 1999-2008 Bruker Daltonik GmbH Page -2-

(**B**)

Figure S7. Annotated MS/MS spectrum of ion 1512.767 *m/z* detected in area #3. (**A**) MS/MS spectrum; (**B**) Peak list and amino acid sequence.

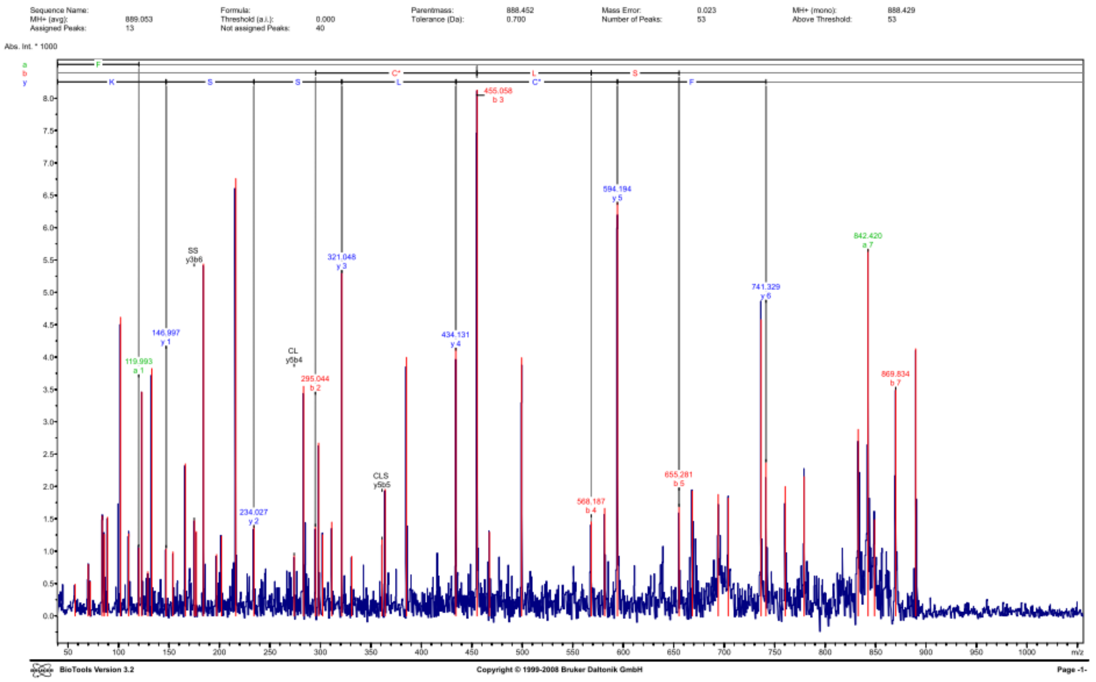

(**A**)

Display Parameter:

Parentmass: 888.452 Mass Error: 0.023 MH+ (mono): 888.429 MH+ (avg): 889.053 Threshold (a.i.): 0.000
Tolerance (Da): 0.700 Number of Peaks: 53 Above Threshold: 53 Assigned Peaks: 13 Not assigned Peaks: 40

Peaklist:

Peak	Mass	Intensity	Peak	Mass	Intensity	Peak	Mass	Intensity	Peak	Mass	Intensity	Peak	Mass	Intensity	Peak	Mass	Intensity
1	56.902	488.413	2	69.942	795.242	3	71.986	526.068	4	83.972	1544.545	5	86.009	1261.505	6	88.965	1525.353
7	102.000	4615.877	8	109.973	1266.961	9	119.993	1061.291	10	123.020	3459.474	11	129.000	683.436	12	132.973	3822.179
13	146.997	1048.126	14	153.984	990.236	15	166.055	2349.984	16	175.014	1473.619	17	177.026	1303.520	18	184.083	5571.883
19	197.007	948.157	20	202.025	1231.880	21	216.052	6758.664	22	234.027	1400.228	23	274.017	931.482	24	283.206	3548.925
25	295.044	1380.273	26	298.168	2668.943	27	302.138	1255.318	28	311.227	1448.461	29	321.048	5308.246	30	331.043	918.732
31	361.047	1179.058	32	364.046	1966.261	33	385.274	3995.934	34	434.131	4140.731	35	455.058	8495.811	36	467.361	1294.450
37	499.343	3991.787	38	568.187	1466.639	39	581.456	1661.112	40	594.194	6395.061	41	655.281	1687.851	42	668.454	1931.467
43	694.094	1876.646	44	704.033	1820.428	45	736.480	4577.611	46	741.329	2376.846	47	760.405	2000.854	48	779.201	2152.524
49	832.711	2882.154	50	842.420	5668.875	51	848.794	1487.540	52	869.834	3536.499	53	889.653	4127.877			

Calculated Masses:

FFCLSSK 3: Carbamidomethyl (C)

N-Term.	Ion	a	b	y	C-Term.	Ion
1	F	**120.081**	148.076	**147.113**	7	K
2	F	267.149	**295.144**	**234.145**	6	S
3	C*	427.180	**455.175**	**321.177**	5	S
4	L	540.264	**568.259**	**434.261**	4	L
5	S	627.296	**655.291**	**594.292**	3	C*
6	S	714.328	742.323	**741.360**	2	F
7	K	**842.423**	**870.418**	888.428	1	F

BioTools Version 3.2 Copyright © 1999-2008 Bruker Daltonik GmbH Page -2-

(**B**)

Figure S8. Annotated MS/MS spectrum of ion 888.452 *m/z* detected in area #3. (**A**) MS/MS spectrum; (**B**) Peak list and amino acid sequence.

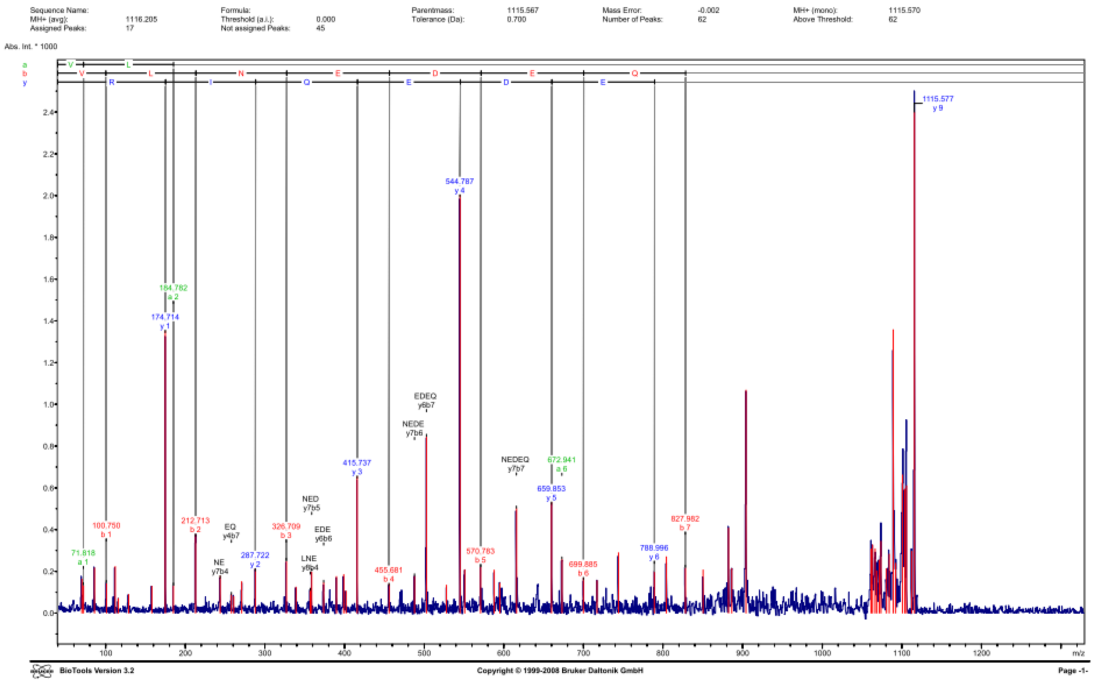

(A)

Display Parameter:

Parentmass: 1115.567 Mass Error: -0.002 MH+ (mono): 1115.570 MH+ (avg): 1116.205 Threshold (a.i.): 0.000
Tolerance (Da): 0.700 Number of Peaks: 62 Above Threshold: 62 Assigned Peaks: 17 Not assigned Peaks: 45

Peaklist:

Peak	Mass	Intensity	Peak	Mass	Intensity	Peak	Mass	Intensity	Peak	Mass	Intensity	Peak	Mass	Intensity	Peak	Mass	Intensity
1	69.801	162.772	2	71.818	133.170	3	85.797	216.070	4	100.750	144.370	5	111.754	223.086	6	115.730	69.729
7	128.740	89.156	8	157.712	128.617	9	174.714	1354.550	10	184.782	131.867	11	212.713	379.884	12	243.662	179.477
13	257.651	87.941	14	260.747	78.878	15	270.707	151.336	16	287.722	213.736	17	326.709	250.746	18	338.721	124.439
19	356.694	107.354	20	358.616	200.946	21	373.653	145.252	22	389.678	175.100	23	398.722	184.012	24	401.641	106.049
25	415.737	657.056	26	455.681	142.477	27	487.677	176.223	28	502.722	845.016	29	527.793	133.938	30	544.787	2004.269
31	550.809	208.013	32	570.783	223.747	33	587.785	206.506	34	594.850	143.392	35	615.799	500.921	36	659.853	532.651
37	672.941	257.170	38	699.885	170.463	39	716.921	155.498	40	743.976	289.273	41	788.996	198.547	42	804.058	270.002
43	827.982	217.547	44	850.110	207.224	45	882.097	407.070	46	886.111	213.136	47	904.157	1067.658	48	1061.141	307.327
49	1062.957	327.131	50	1066.180	307.778	51	1068.226	202.362	52	1071.515	253.411	53	1073.595	344.276	54	1080.388	212.059
55	1083.852	282.207	56	1089.093	1357.291	57	1091.978	238.944	58	1100.520	661.542	59	1103.585	594.707	60	1105.656	609.623
61	1112.270	295.817	62	1115.577	2399.545												

Calculated Masses:
VLNEDEQIR

N-Term.	Ion	a	b	y	C-Term.	Ion
1	V	**72.081**	**100.076**	**175.119**	9	R
2	L	**185.165**	**213.160**	**288.203**	8	I
3	N	299.208	**327.203**	**416.262**	7	Q
4	E	428.250	**456.245**	**545.304**	6	E
5	D	543.277	**571.272**	**660.331**	5	D
6	E	**672.320**	**700.315**	**789.374**	4	E
7	Q	800.378	**828.373**	903.417	3	N
8	I	913.463	941.457	1016.501	2	L
9	R	1069.564	1097.559	**1115.569**	1	V

BioTools Version 3.2 Copyright © 1999-2008 Bruker Daltonik GmbH Page -2-

(B)

Figure S9. Annotated MS/MS spectrum of ion 1115.567 *m*/*z* detected in area #4. (**A**) MS/MS spectrum; (**B**) Peak list and amino acid sequence.

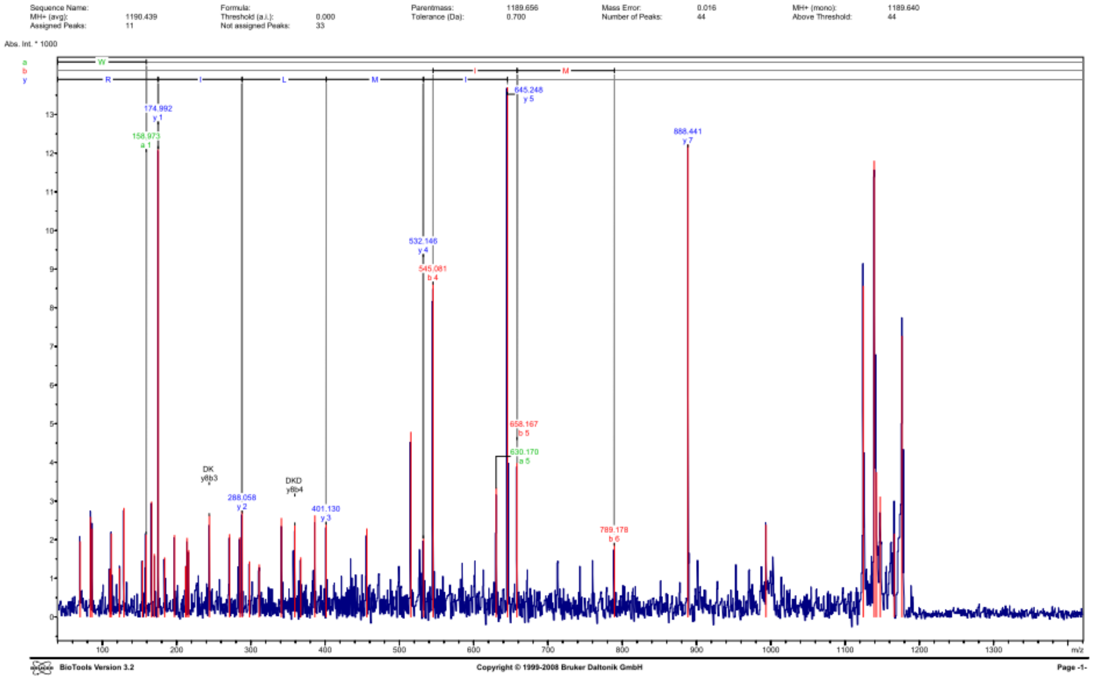

(**A**)

Display Parameter:

Parentmass: 1189.656 Mass Error: 0.016 MH+ (mono): 1189.640 MH+ (avg): 1190.439 Threshold (a.i.): 0.000
Tolerance (Da): 0.700 Number of Peaks: 44 Above Threshold: 44 Assigned Peaks: 11 Not assigned Peaks: 33

Peaklist:

Peak	Mass	Intensity	Peak	Mass	Intensity	Peak	Mass	Intensity	Peak	Mass	Intensity	Peak	Mass	Intensity	Peak	Mass	Intensity
1	69.988	1951.459	2	83.975	2581.839	3	86.009	2275.108	4	109.939	1227.294	5	111.972	2149.867	6	122.999	1256.430
7	128.997	2813.319	8	153.962	1444.612	9	158.973	2136.322	10	166.011	2977.716	11	169.931	1621.322	12	174.992	12107.406
13	183.988	1531.105	14	196.992	2114.026	15	211.998	1302.597	16	213.998	2036.555	17	215.989	1723.260	18	243.996	2613.610
19	271.030	2135.326	20	285.019	2055.698	21	288.058	2748.332	22	298.051	1431.459	23	311.067	1352.426	24	341.019	2551.690
25	359.018	2369.847	26	367.077	1536.193	27	386.099	2618.908	28	401.130	2404.586	29	456.090	2280.326	30	515.140	4780.260
31	532.146	2035.626	32	545.081	8669.033	33	630.170	3330.533	34	645.248	13950.409	35	658.167	3993.054	36	789.178	1913.414
37	888.441	12218.851	38	993.368	2377.892	39	1124.561	8559.726	40	1139.183	11796.402	41	1142.121	3739.024	42	1147.181	3103.887
43	1166.125	2145.961	44	1176.590	7263.730												

Calculated Masses:
WDKDIMLIR

N-Term.	Ion	a	b	y	C-Term.	Ion
1	W	**159.092**	187.087	**175.119**	9	R
2	D	274.119	302.114	**288.203**	8	I
3	K	402.214	430.208	**401.287**	7	L
4	D	517.241	**545.235**	**532.328**	6	M
5	I	**630.325**	**658.320**	**645.412**	5	I
6	M	761.365	**789.360**	760.439	4	D
7	L	874.449	902.444	**888.534**	3	K
8	I	987.533	1015.528	1003.560	2	D
9	R	1143.634	1171.629	1189.640	1	W

BioTools Version 3.2 Copyright © 1999-2008 Bruker Daltonik GmbH Page -2-

(**B**)

Figure S10. Annotated MS/MS spectrum of ion 1189.656 *m*/*z* detected in area #4. (**A**) MS/MS spectrum; (**B**) Peak list and amino acid sequence.

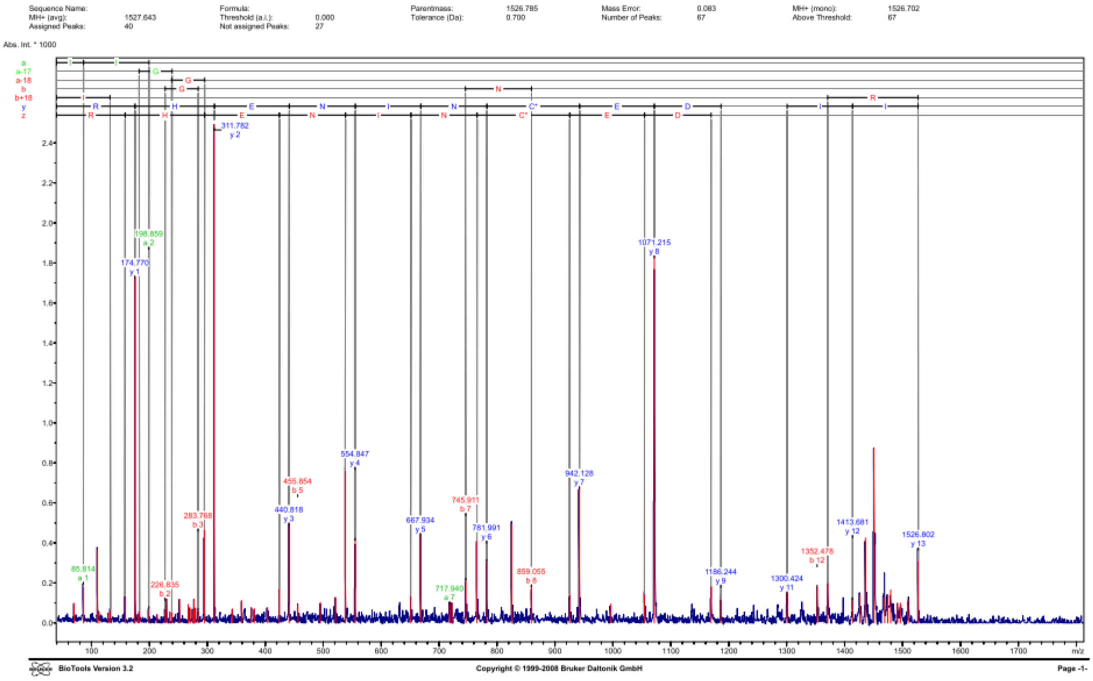

(**A**)

Display Parameter:

Parentmass: 1526.785 Mass Error: 0.083 MH+ (mono): 1526.702 MH+ (avg): 1527.643 Threshold (a.i.): 0.000
Tolerance (Da): 0.700 Number of Peaks: 67 Above Threshold: 67 Assigned Peaks: 40 Not assigned Peaks: 27

Peaklist:

Peak	Mass	Intensity	Peak	Mass	Intensity	Peak	Mass	Intensity	Peak	Mass	Intensity	Peak	Mass	Intensity	Peak	Mass	Intensity
1	69.829	98.345	2	85.814	182.933	3	109.806	367.976	4	112.804	53.661	5	128.869	49.567	6	132.745	67.378
7	157.773	134.170	8	174.770	1734.463	9	179.720	40.705	10	182.785	59.381	11	198.859	68.603	12	212.705	42.236
13	226.835	71.669	14	229.733	120.539	15	234.773	55.046	16	238.634	53.641	17	251.781	118.467	18	266.727	92.421
19	269.784	75.934	20	274.686	62.603	21	276.755	117.054	22	278.826	71.518	23	283.768	59.781	24	294.772	466.515
25	311.782	2515.691	26	342.787	68.638	27	358.743	112.912	28	375.773	74.147	29	380.763	71.948	30	403.764	76.614
31	423.801	172.965	32	440.818	500.165	33	455.854	83.595	34	494.828	102.887	35	520.792	129.107	36	537.822	764.447
37	554.847	410.009	38	650.869	135.674	39	667.934	437.293	40	717.940	105.481	41	721.955	100.226	42	745.911	210.880
43	765.011	416.266	44	781.991	304.364	45	824.990	493.219	46	859.055	190.696	47	925.085	144.130	48	942.128	681.771
49	996.188	94.912	50	1054.179	145.896	51	1071.215	1835.533	52	1169.301	182.987	53	1186.244	115.419	54	1300.424	155.583
55	1352.478	176.369	56	1370.523	198.607	57	1413.681	111.440	58	1425.717	141.983	59	1435.682	424.777	60	1450.278	869.670
61	1468.902	130.838	62	1474.258	139.369	63	1479.294	164.836	64	1490.915	98.521	65	1496.049	99.189	66	1509.469	115.874
67	1526.802	312.636															

Calculated Masses:
IIGGDECNINEHR 7: Carbamidomethyl (C)

N-Term.	Ion	a	a-17	a-18	b	b-17	b-18	b+18	c	i	x	y	z	C-Term.	Ion
1	I	**86.096**	69.070	68.086	114.091	97.065	96.081	**132.102**	131.118	**86.096**	201.098	**175.119**	**158.092**	13	R
2	I	**199.180**	**182.154**	181.170	**227.175**	210.149	209.165	245.186	244.202	**86.096**	338.157	**312.178**	**295.151**	12	H
3	G	256.202	**239.175**	**238.191**	**284.197**	**267.170**	**266.186**	302.207	301.223	30.034	467.200	**441.220**	**424.194**	11	E
4	G	313.223	296.197	**295.213**	341.218	324.192	323.208	**359.229**	**358.245**	30.034	581.243	**555.263**	**538.237**	10	N
5	D	428.250	411.224	410.240	**456.245**	439.219	438.235	474.256	473.272	88.039	694.327	**668.347**	**651.321**	9	I
6	E	557.293	540.266	539.282	585.288	568.261	567.277	603.298	602.314	102.055	808.370	**782.390**	**765.364**	8	N
7	C*	**717.324**	700.297	699.313	**745.319**	728.292	727.308	763.329	762.345	**133.043**	968.400	**942.421**	**925.394**	7	C*
8	N	831.367	814.340	813.356	**859.361**	842.335	841.351	877.372	876.388	87.055	1097.443	**1071.464**	**1054.437**	6	E
9	I	944.451	927.424	926.440	972.446	955.419	954.435	990.456	989.472	**86.096**	1212.470	**1186.491**	**1169.464**	5	D
10	N	1058.494	1041.467	1040.483	1086.488	1069.462	1068.478	1104.499	1103.515	87.055	1269.491	1243.512	1226.485	4	G
11	E	1187.536	1170.510	**1169.526**	1215.531	1198.504	1197.520	1233.542	1232.558	102.055	1326.513	**1300.533**	1283.507	3	G
12	H	1324.595	1307.568	1306.584	**1352.590**	1335.563	1334.579	**1370.601**	1369.616	**110.071**	1439.597	**1413.618**	1396.591	2	I
13	R	1480.696	1463.670	1462.686	1508.691	1491.665	**1490.680**	**1526.702**	1525.718	**129.113**	1552.681	**1526.702**	**1509.675**	1	I

BioTools Version 3.2 Copyright © 1999-2008 Bruker Daltonik GmbH Page -2-

(**B**)

Figure S11. Annotated MS/MS spectrum of ion 1526.785 *m*/*z* detected in area #4. (**A**) MS/MS spectrum; (**B**) Peak list and amino acid sequence.

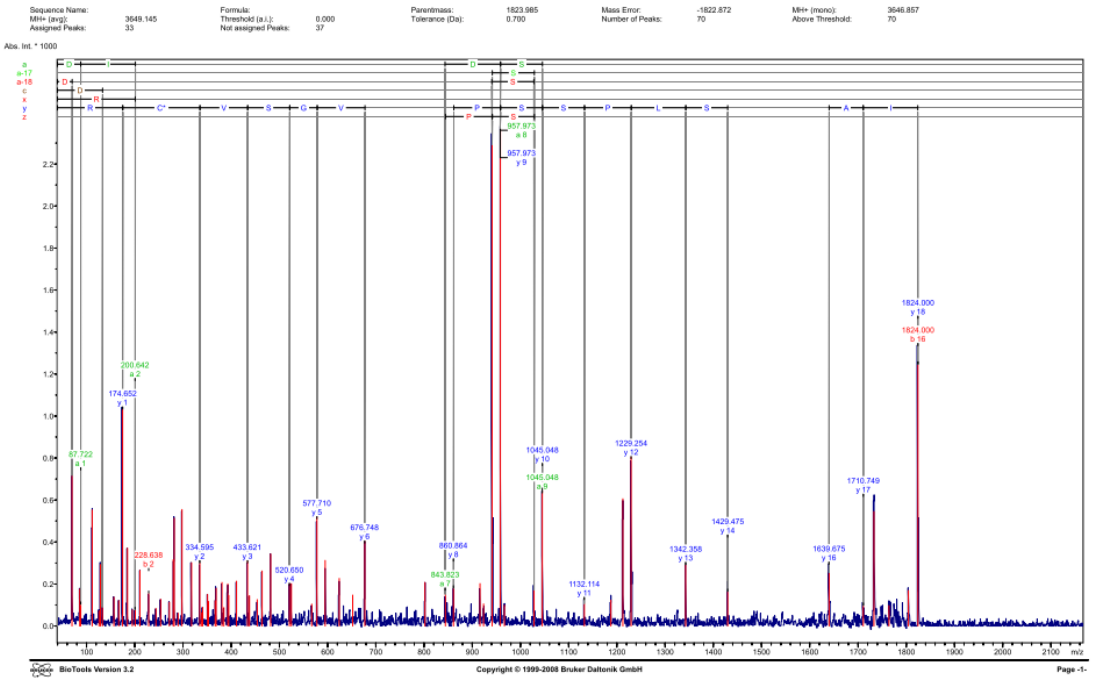

(**A**)

Display Parameter:

Parentmass: 1823.985 Mass Error: -1822.872 MH+ (mono): 3646.857 MH+ (avg): 3649.145 Threshold (a.i.): 0.000
Tolerance (Da): 0.700 Number of Peaks: 70 Above Threshold: 70 Assigned Peaks: 33 Not assigned Peaks: 37

Peaklist:

Peak	Mass	Intensity	Peak	Mass	Intensity	Peak	Mass	Intensity	Peak	Mass	Intensity	Peak	Mass	Intensity	Peak	Mass	Intensity
1	69.735	716.461	2	85.750	176.134	3	87.722	102.810	4	111.685	552.728	5	125.691	71.902	6	128.692	293.236
7	132.588	88.917	8	156.654	137.888	9	166.627	121.717	10	174.652	1033.850	11	184.629	371.186	12	194.630	77.177
13	200.642	77.593	14	210.621	265.236	15	228.638	154.311	16	243.593	83.846	17	253.594	127.588	18	271.606	113.684
19	279.625	316.083	20	281.626	514.057	21	289.611	61.115	22	297.626	555.251	23	317.567	301.840	24	334.595	301.697
25	338.588	64.681	26	340.621	89.014	27	350.609	150.687	28	352.652	117.343	29	366.638	120.254	30	368.617	179.850
31	380.633	207.579	32	384.675	88.277	33	392.689	200.589	34	394.724	146.167	35	410.672	214.083	36	433.621	311.873
37	437.698	144.424	38	453.698	125.644	39	463.692	262.433	40	481.707	343.074	41	520.650	204.153	42	524.700	203.851
43	566.830	104.495	44	577.710	521.748	45	594.783	312.199	46	623.722	226.630	47	651.736	147.956	48	676.748	405.566
49	801.785	207.355	50	843.823	151.683	51	860.864	180.269	52	915.934	202.411	53	923.006	105.140	54	940.935	2293.048
55	957.973	2368.479	56	966.560	100.153	57	1027.990	172.647	58	1045.048	645.005	59	1132.114	106.775	60	1187.189	123.005
61	1212.169	606.199	62	1229.254	807.435	63	1342.358	293.441	64	1429.475	164.036	65	1639.675	255.052	66	1710.749	97.246
67	1732.879	545.429	68	1764.019	97.352	69	1804.050	169.233	70	1824.000	1246.122						

Calculated Masses:

DIMLIRLDSPVSNSEHIAPLSLPSSPPSVGSVCR 3: Oxidation (M) 33: Carbamidomethyl (C)

N-Term.	Ion	a	a-17	a-18	b	b-17	b-18	b+18	c	i	x	y	z	C-Term.	Ion
1	D	**88.039**	71.013	**70.029**	116.034	99.008	98.024	134.045	**133.061**	**88.039**	**201.098**	**175.119**	158.092	34	R
2	I	**201.123**	**184.097**	183.113	**229.118**	212.092	**211.108**	247.129	246.145	**86.096**	361.129	**335.150**	**318.123**	33	C*
3	M*	348.159	331.132	330.148	376.154	359.127	358.143	**394.164**	**393.180**	120.048	460.197	**434.218**	417.191	32	V
4	L	461.243	444.216	443.232	489.238	472.211	471.227	507.248	506.264	**86.096**	547.229	**521.250**	504.223	31	S
5	I	574.327	557.300	556.316	602.322	585.295	584.311	620.332	619.348	**86.096**	604.251	**578.272**	561.245	30	G
6	R	730.428	713.401	712.417	758.423	741.396	740.412	776.433	775.449	**129.113**	703.319	**677.340**	660.313	29	V
7	L	**843.512**	826.486	825.502	871.507	854.480	853.496	889.518	888.534	**86.096**	790.351	764.372	747.345	28	S
8	D	**958.539**	**941.512**	**940.528**	986.534	969.507	968.523	1004.544	1003.560	**88.039**	887.404	**861.425**	**844.398**	27	P
9	S	**1045.571**	**1028.544**	**1027.560**	1073.566	1056.539	1055.555	1091.577	1090.593	60.044	984.457	**958.477**	**941.451**	26	P
10	P	1142.624	1125.597	1124.613	1170.619	1153.592	1152.608	1188.629	**1187.645**	**70.065**	1071.489	**1045.510**	**1028.483**	25	S
11	V	1241.692	1224.666	1223.682	1269.687	1252.661	1251.677	1287.698	1286.714	72.081	1158.521	**1132.542**	1115.515	24	S
12	S	1328.724	1311.698	1310.714	1356.719	1339.693	1338.709	1374.730	1373.746	60.044	1255.574	**1229.594**	**1212.568**	23	P
13	N	1442.767	1425.741	1424.757	1470.762	1453.736	1452.752	1488.773	1487.789	**87.055**	1368.658	**1342.678**	1325.652	22	L
14	S	1529.799	1512.773	1511.789	1557.794	1540.768	1539.784	1575.805	1574.821	60.044	1455.690	**1429.710**	1412.684	21	S
15	E	1658.842	1641.815	1640.831	1686.837	1669.810	1668.826	1704.847	1703.863	102.055	1568.774	1542.794	1525.768	20	L
16	H	1795.901	1778.874	1777.890	**1823.896**	1806.869	1805.885	1841.906	1840.922	110.071	1665.826	**1639.847**	1622.821	19	P
17	I	1908.985	1891.958	1890.974	1936.980	1919.953	1918.969	1954.990	1954.006	**86.096**	1736.864	**1710.884**	1693.858	18	A
18	A	1980.022	1962.995	1962.011	2008.017	1990.990	1990.006	2026.027	2025.043	44.049	1849.948	**1823.968**	1806.942	17	I
19	P	2077.075	2060.048	2059.064	2105.070	2088.043	2087.059	2123.080	2122.096	**70.065**	1987.007	1961.027	1944.001	16	H
20	L	2190.159	2173.132	2172.148	2218.154	2201.127	2200.143	2236.164	2235.180	**86.096**	2116.049	2090.070	2073.043	15	E
21	S	2277.191	2260.164	2259.180	2305.186	2288.159	2287.175	2323.196	2322.212	60.044	2203.081	2177.102	2160.075	14	S
22	L	2390.275	2373.248	2372.264	2418.270	2401.243	2400.259	2436.280	2435.296	**86.096**	2317.124	2291.145	2274.118	13	N
23	P	2487.328	2470.301	2469.317	2515.322	2498.296	2497.312	2533.333	2532.349	**70.065**	2404.156	2378.177	2361.150	12	S
24	S	2574.360	2557.333	2556.349	2602.355	2585.328	2584.344	2620.365	2619.381	60.044	2503.225	2477.245	2460.219	11	V
25	S	2661.392	2644.365	2643.381	2689.387	2672.360	2671.376	2707.397	2706.413	60.044	2600.277	2574.298	2557.272	10	P
26	P	2758.444	2741.418	2740.434	2786.439	2769.413	2768.429	2804.450	2803.466	**70.065**	2687.309	2661.330	2644.304	9	S
27	P	2855.497	2838.471	2837.487	2883.492	2866.466	2865.482	2901.503	2900.519	**70.065**	2802.336	2776.357	2759.330	8	D
28	S	2942.529	2925.503	2924.519	2970.524	2953.498	2952.514	2988.535	2987.551	60.044	2915.420	2889.441	2872.415	7	L
29	V	3041.598	3024.571	3023.587	3069.593	3052.566	3051.582	3087.603	3086.619	72.081	3071.521	3045.542	3028.516	6	R
30	G	3098.619	3081.593	3080.608	3126.614	3109.587	3108.603	3144.625	3143.641	30.034	3184.606	3158.626	3141.600	5	I
31	S	3185.651	3168.625	3167.641	3213.646	3196.619	3195.635	3231.657	3230.673	60.044	3297.690	3271.710	3254.684	4	L
32	V	3284.720	3267.693	3266.709	3312.714	3295.688	3294.704	3330.725	3329.741	72.081	3444.725	3418.746	3401.719	3	M*
33	C*	3444.750	3427.724	3426.740	3472.745	3455.719	3454.735	3490.756	3489.772	**133.043**	3557.809	3531.830	3514.803	2	I
34	R	3600.851	3583.825	3582.841	3628.846	3611.820	3610.836	3646.857	3645.873	**129.113**	3672.836	3646.857	3629.830	1	D

BioTools Version 3.2 Copyright © 1999-2008 Bruker Daltonik GmbH Page -2-

(**B**)

Figure S12. Annotated MS/MS spectrum of ion 1823.985 *m*/*z* detected in area #4. (**A**) MS/MS spectrum; (**B**) Peak list and amino acid sequence.

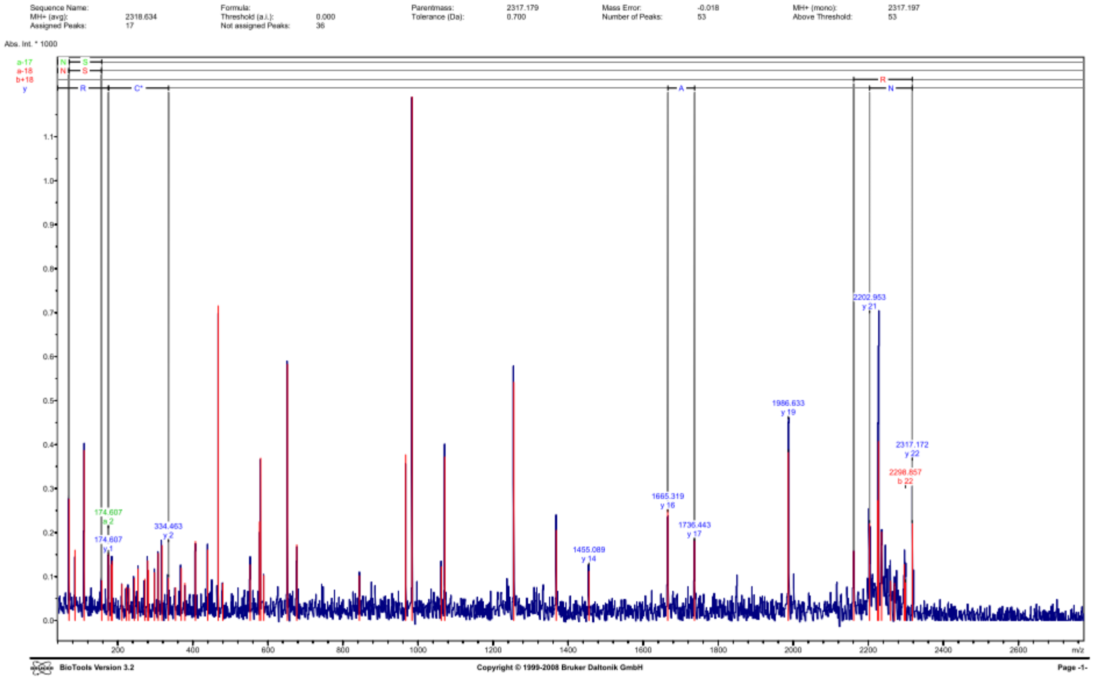

(**A**)

Display Parameter:

Parentmass: 2317.179 Mass Error: -0.018 MH+ (mono): 2317.197 MH+ (avg): 2318.634 Threshold (a.i.): 0.000
Tolerance (Da): 0.700 Number of Peaks: 53 Above Threshold: 53 Assigned Peaks: 17 Not assigned Peaks: 36

Peaklist:

Peak	Mass	Intensity	Peak	Mass	Intensity	Peak	Mass	Intensity	Peak	Mass	Intensity	Peak	Mass	Intensity	Peak	Mass	Intensity
1	69.706	278.377	2	85.656	159.886	3	109.663	386.801	4	156.616	92.616	5	174.607	153.692	6	182.602	127.297
7	184.541	134.946	8	210.599	82.568	9	222.607	75.365	10	229.585	73.080	11	243.498	95.809	12	253.560	117.264
13	271.457	93.931	14	279.506	135.479	15	281.486	114.292	16	297.541	108.840	17	307.514	152.649	18	317.452	170.476
19	334.463	98.343	20	353.461	74.418	21	368.500	119.247	22	378.530	84.718	23	406.543	179.802	24	439.446	160.864
25	467.438	715.459	26	479.314	84.190	27	552.510	126.849	28	577.492	224.443	29	580.508	369.088	30	588.711	102.271
31	651.533	582.377	32	676.564	172.040	33	843.685	101.740	34	966.723	376.646	35	983.759	1253.696	36	1061.849	122.308
37	1070.801	372.109	38	1254.930	541.696	39	1368.000	205.293	40	1455.089	111.766	41	1665.319	252.267	42	1736.443	182.339
43	1986.633	382.786	44	2160.778	158.796	45	2202.953	221.661	46	2224.681	272.528	47	2226.948	406.767	48	2235.143	201.232
49	2259.176	89.708	50	2269.543	84.401	51	2295.228	127.642	52	2298.857	86.875	53	2317.172	221.589			

Calculated Masses:
NSEHIAPLSLPSSPPIVGSVCR 21: Carbamidomethyl (C)

N-Term.	Ion	a	a-17	a-18	b	b-17	b-18	b+18	c	i	x	y	z	C-Term.	Ion
1	N	87.055	**70.029**	**69.045**	115.050	98.024	97.040	133.061	132.077	87.055	201.098	**175.119**	158.092	22	R
2	S	**174.087**	**157.061**	**156.077**	202.082	**185.056**	**184.072**	220.093	219.109	60.044	361.129	**335.150**	**318.123**	21	C*
3	E	303.130	286.103	285.119	331.125	314.098	313.114	349.135	348.151	102.055	460.197	434.218	417.191	20	V
4	H	440.189	423.162	422.178	468.184	451.157	450.173	486.194	485.210	**110.071**	547.229	521.250	504.223	19	S
5	I	553.273	536.246	535.262	581.268	564.241	563.257	599.278	598.294	**86.096**	604.251	578.272	561.245	18	G
6	A	624.310	607.283	606.299	652.305	635.278	634.294	670.315	669.331	44.049	703.319	677.340	660.313	17	V
7	P	721.363	704.336	703.352	749.358	732.331	731.347	767.368	766.384	**70.065**	816.403	790.424	773.397	16	I
8	L	834.447	817.420	816.436	862.442	845.415	844.431	880.452	879.468	**86.096**	913.456	887.477	870.450	15	P
9	S	921.479	904.452	903.468	949.474	932.447	931.463	967.484	**966.500**	60.044	1010.509	984.530	967.503	14	P
10	L	1034.563	1017.536	1016.552	1062.558	1045.531	1044.547	1080.568	1079.584	**86.096**	1097.541	1071.562	1054.535	13	S
11	P	1131.616	1114.589	1113.605	1159.611	1142.584	1141.600	1177.621	1176.637	**70.065**	1184.573	1158.594	1141.567	12	S
12	S	1218.648	1201.621	1200.637	1246.643	1229.616	1228.632	1264.653	1263.669	60.044	1281.626	1255.646	1238.620	11	P
13	S	1305.680	1288.653	1287.669	1333.675	1316.648	1315.664	1351.685	1350.701	60.044	1394.710	1368.730	1351.704	10	L
14	P	1402.733	1385.706	1384.722	1430.727	1413.701	1412.717	1448.738	1447.754	**70.065**	1481.742	**1455.762**	1438.736	9	S
15	P	1499.785	1482.759	1481.775	1527.780	1510.754	1509.770	1545.791	1544.807	**70.065**	1594.826	1568.846	1551.820	8	L
16	I	1612.869	1595.843	1594.859	1640.864	1623.838	1622.854	1658.875	1657.891	**86.096**	1691.879	**1665.899**	1648.873	7	P
17	V	1711.938	1694.911	1693.927	1739.933	1722.906	1721.922	1757.943	1756.959	72.081	1762.916	**1736.936**	1719.910	6	A
18	G	1768.959	1751.933	1750.949	1796.954	1779.928	1778.944	1814.965	1813.981	30.034	1876.000	1850.020	1832.994	5	I
19	S	1855.991	1838.965	1837.981	1883.986	1866.960	1865.976	1901.997	1901.013	60.044	2013.059	**1987.079**	1970.053	4	H
20	V	1955.060	1938.033	1937.049	1983.055	1966.028	1965.044	2001.065	2000.081	72.081	2142.101	2116.122	2099.095	3	E
21	C*	2115.090	2098.064	2097.080	2143.085	2126.059	2125.075	**2161.096**	**2160.112**	133.043	2229.133	**2203.154**	2186.127	2	S
22	R	2271.191	2254.165	2253.181	**2299.186**	2282.160	2281.176	**2317.197**	2316.213	129.113	2343.176	**2317.197**	2300.170	1	N

BioTools Version 3.2 Copyright © 1999-2008 Bruker Daltonik GmbH Page -2-

(**B**)

Figure S13. Annotated MS/MS spectrum of ion 2317.179 *m*/*z* detected in area #4. (**A**) MS/MS spectrum; (**B**) Peak list and amino acid sequence.

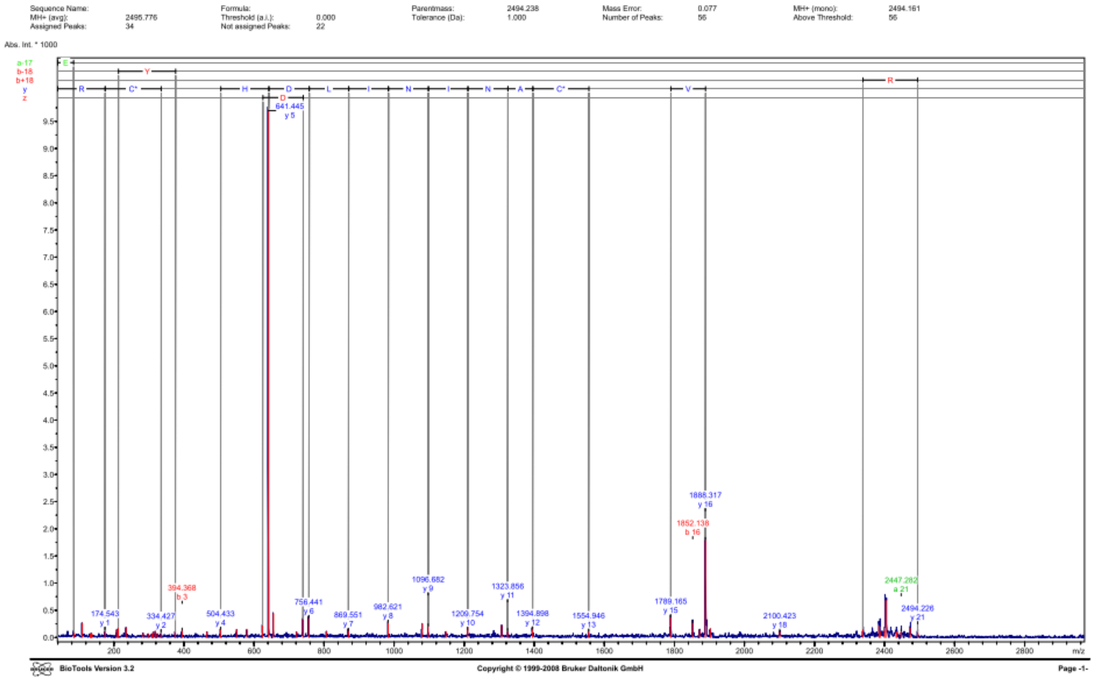

(**A**)

Display Parameter:

Parentmass: 2494.238 Mass Error: 0.077 MH+ (mono): 2494.161 MH+ (avg): 2495.776 Threshold (a.i.): 0.000
Tolerance (Da): 1.000 Number of Peaks: 56 Above Threshold: 56 Assigned Peaks: 34 Not assigned Peaks: 22

Peaklist:

Peak	Mass	Intensity	Peak	Mass	Intensity	Peak	Mass	Intensity	Peak	Mass	Intensity	Peak	Mass	Intensity	Peak	Mass	Intensity
1	85.257	85.270	2	86.477	93.812	3	109.580	264.758	4	132.442	61.973	5	135.564	80.752	6	137.513	62.684
7	174.543	113.063	8	208.485	144.105	9	212.470	175.140	10	234.421	176.402	11	283.438	77.847	12	297.418	69.291
13	307.445	61.146	14	311.429	88.433	15	317.338	113.598	16	323.369	68.656	17	334.427	68.138	18	375.385	109.309
19	394.368	124.761	20	465.382	100.494	21	504.433	153.510	22	550.468	146.789	23	579.407	149.176	24	624.432	231.695
25	641.445	9727.433	26	655.421	441.772	27	721.412	106.782	28	739.416	341.757	29	756.441	367.219	30	807.481	113.001
31	869.551	172.093	32	982.621	282.371	33	1079.614	259.067	34	1096.682	203.975	35	1147.719	113.227	36	1209.754	191.016
37	1306.770	222.237	38	1323.856	116.429	39	1394.898	198.577	40	1554.946	118.141	41	1789.165	381.080	42	1852.138	269.385
43	1871.517	134.541	44	1888.317	1793.340	45	1902.635	162.367	46	2100.423	112.729	47	2337.780	158.569	48	2384.744	214.543
49	2387.051	111.762	50	2399.492	129.801	51	2403.048	685.865	52	2432.803	102.181	53	2435.514	105.724	54	2447.282	112.303
55	2472.959	174.610	56	2494.226	99.648												

Calculated Masses:

ETYPDVPHCANINILDHAVCR 9: Carbamidomethyl (C) 20: Carbamidomethyl (C)

N-Term.	Ion	a	a-17	a-18	b	b-17	b-18	b+18	c	i	x	y	z	C-Term.	Ion
1	E	102.055	**85.028**	84.044	130.050	113.023	112.039	148.060	147.076	102.055	201.098	**175.119**	158.092	21	R
2	T	203.103	186.076	185.092	231.098	214.071	**213.087**	249.108	248.124	74.060	361.129	**335.150**	**318.123**	20	C*
3	Y	366.166	349.139	348.155	**394.161**	377.134	**376.150**	412.171	411.187	**136.076**	460.197	434.218	417.191	19	V
4	P	463.219	446.192	445.208	491.214	474.187	473.203	509.224	508.240	70.065	531.234	**505.255**	488.229	18	A
5	D	578.246	561.219	560.235	606.241	589.214	588.230	**624.251**	623.267	88.039	668.293	**642.314**	**625.287**	17	H
6	V	677.314	660.288	659.304	705.309	688.282	687.298	723.320	**722.336**	72.081	783.320	**757.341**	**740.314**	16	D
7	P	774.367	**757.340**	**756.356**	802.362	785.335	784.351	820.372	819.388	70.065	896.404	**870.425**	853.398	15	L
8	H	911.426	894.399	893.415	939.421	922.394	921.410	957.431	956.447	**110.071**	1009.488	**983.509**	966.483	14	I
9	C*	1071.456	1054.430	1053.446	1099.451	1082.425	1081.441	1117.462	1116.478	**133.043**	1123.531	**1097.552**	**1080.525**	13	N
10	A	1142.494	1125.467	1124.483	1170.488	1153.462	1152.478	1188.499	1187.515	44.049	1236.615	**1210.636**	1193.610	12	I
11	N	1256.536	1239.510	1238.526	1284.531	1267.505	1266.521	1302.542	1301.558	**87.055**	1350.658	**1324.679**	**1307.652**	11	N
12	I	1369.621	1352.594	1351.610	1397.615	1380.589	1379.605	1415.626	1414.642	**86.096**	1421.695	**1395.716**	1378.690	10	A
13	N	1483.663	1466.637	1465.653	1511.658	1494.632	1493.648	1529.669	1528.685	**87.055**	1581.726	**1555.747**	1538.720	9	C*
14	I	1596.748	1579.721	1578.737	1624.742	1607.716	1606.732	1642.753	1641.769	**86.096**	1718.785	1692.806	1675.779	8	H
15	L	1709.832	1692.805	1691.821	1737.826	1720.800	1719.816	1755.837	1754.853	**86.096**	1815.838	**1789.858**	1772.832	7	P
16	D	1824.859	1807.832	1806.848	**1852.853**	1835.827	1834.843	**1870.864**	1869.880	88.039	1914.906	**1888.927**	**1871.900**	6	V
17	H	1961.917	1944.891	1943.907	1989.912	1972.886	1971.902	2007.923	2006.939	**110.071**	2029.933	2003.954	1986.927	5	D
18	A	2032.955	2015.928	2014.944	2060.949	2043.923	2042.939	2078.960	2077.976	44.049	2126.986	**2101.007**	2083.980	4	P
19	V	2132.023	2114.996	2114.012	2160.018	2142.991	2142.007	2178.028	2177.044	72.081	2290.049	2264.070	2247.043	3	Y
20	C*	2292.054	2275.027	2274.043	2320.049	2303.022	2302.038	**2338.059**	**2337.075**	**133.043**	2391.097	2365.118	2348.091	2	T
21	R	**2448.155**	2431.128	2430.144	2476.150	2459.123	2458.139	**2494.160**	2493.176	129.113	2520.139	**2494.160**	2477.134	1	E

BioTools Version 3.2 Copyright © 1999-2008 Bruker Daltonik GmbH Page -2-

(**B**)

Figure S14. Annotated MS/MS spectrum of ion 2494.238 *m*/*z* detected in area #4. (**A**) MS/MS spectrum; (**B**) Peak list and amino acid sequence.

Sequence Name:
MH+ (avg): 2579.846
Assigned Peaks: 46
Formula:
Threshold (a.i.): 0.000
Not assigned Peaks: 27
Parentmass: 2578.230
Tolerance (Da): 0.700
Mass Error: 0.059
Number of Peaks: 73
MH+ (mono): 2578.171
Above Threshold: 73

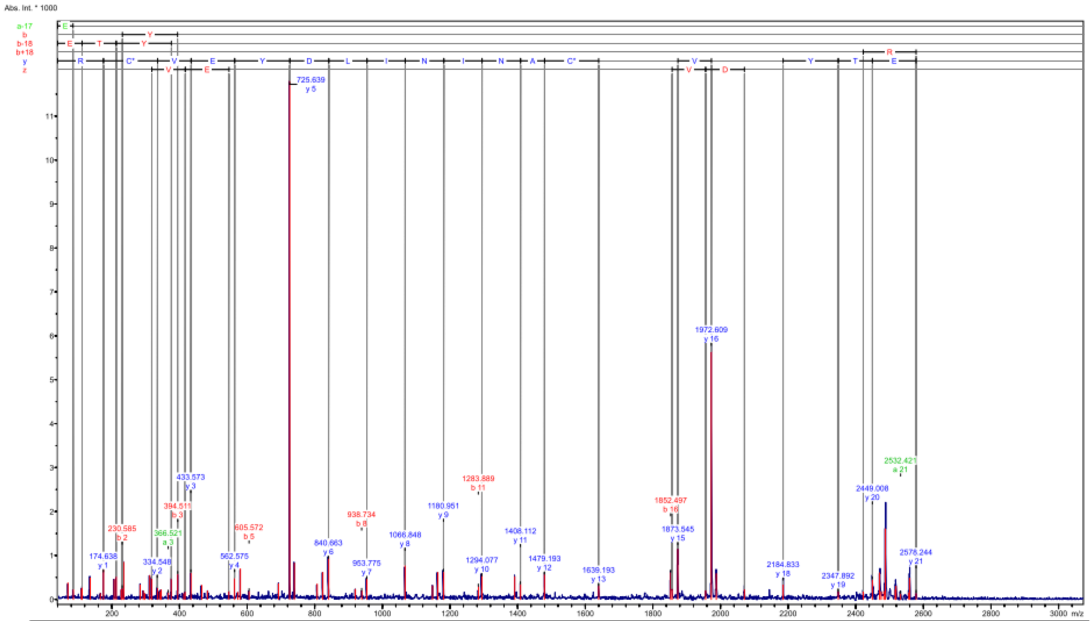

BioTools Version 3.2 Copyright © 1999-2008 Bruker Daltonik GmbH Page -1-

(**A**)

Display Parameter:

Parentmass: 2578.230
Tolerance (Da): 0.700
Mass Error: 0.059
Number of Peaks: 73
MH+ (mono): 2578.171
Above Threshold: 73
MH+ (avg): 2579.846
Assigned Peaks: 46
Threshold (a.i.): 0.000
Not assigned Peaks: 27

Peaklist:

Peak	Mass	Intensity	Peak	Mass	Intensity	Peak	Mass	Intensity	Peak	Mass	Intensity	Peak	Mass	Intensity	Peak	Mass	Intensity
1	69.700	376.040	2	85.679	231.014	3	109.585	230.031	4	111.730	279.548	5	135.639	489.977	6	165.594	134.352
7	174.638	678.918	8	206.625	449.403	9	212.566	520.955	10	227.619	217.414	11	230.585	255.159	12	234.585	846.002
13	283.579	333.839	14	292.536	192.718	15	297.516	134.256	16	311.543	548.273	17	317.526	479.529	18	334.548	267.158
19	341.566	173.600	20	345.528	217.863	21	366.521	152.814	22	375.519	467.887	23	394.511	578.618	24	412.571	135.836
25	416.580	183.486	26	433.573	614.878	27	465.543	321.953	28	482.541	193.746	29	545.595	188.835	30	562.575	489.774
31	572.566	165.586	32	579.560	699.405	33	605.572	183.967	34	692.645	360.840	35	725.639	11812.787	36	739.595	827.768
37	806.691	353.121	38	823.627	616.417	39	840.663	983.566	40	919.731	230.191	41	938.734	193.042	42	953.775	476.317
43	1066.848	785.071	44	1147.896	310.984	45	1163.929	617.815	46	1180.951	631.844	47	1283.889	291.192	48	1294.077	592.239
49	1391.134	519.119	50	1408.112	344.819	51	1479.193	579.966	52	1639.193	319.342	53	1852.497	612.474	54	1856.517	243.848
55	1873.545	1152.734	56	1955.801	218.038	57	1972.609	5642.374	58	1987.098	587.198	59	2070.584	238.864	60	2184.833	348.976
61	2347.892	188.212	62	2421.804	186.955	63	2449.008	456.297	64	2470.225	607.481	65	2474.865	173.612	66	2482.763	183.988
67	2487.270	1607.908	68	2516.257	219.834	69	2518.800	304.551	70	2532.421	130.747	71	2556.582	147.670	72	2559.132	485.514
73	2578.244	149.686															

Calculated Masses:

ETYPDVPHCANINILDYEVCR 9: Carbamidomethyl (C) 20: Carbamidomethyl (C)

N-Term.	Ion	a	a-17	a-18	b	b-17	b-18	b+18	c	i	x	y	z	C-Term.	Ion
1	E	102.055	**85.028**	84.044	130.050	113.023	**112.039**	148.060	147.076	102.055	201.098	**175.119**	158.092	21	R
2	T	203.103	186.076	185.092	**231.098**	214.071	**213.087**	249.108	248.124	74.060	361.129	**335.150**	**318.123**	20	C*
3	Y	**366.166**	349.139	348.155	**394.161**	377.134	**376.150**	**412.171**	411.187	**136.076**	460.197	**434.218**	**417.191**	19	V
4	P	463.219	446.192	445.208	491.214	474.187	473.203	509.224	508.240	**70.065**	589.240	**563.261**	**546.234**	18	E
5	D	578.246	561.219	560.235	**606.241**	589.214	588.230	624.251	623.267	88.039	752.303	**726.324**	709.297	17	Y
6	V	677.314	660.288	659.304	705.309	688.282	687.298	723.320	722.336	72.081	867.330	**841.351**	**824.324**	16	D
7	P	774.367	757.340	756.356	802.362	785.335	784.351	820.372	819.388	**70.065**	980.414	**954.435**	937.408	15	L
8	H	911.426	894.399	893.415	**939.421**	922.394	921.410	957.431	956.447	**110.071**	1093.498	**1067.519**	1050.492	14	I
9	C*	1071.456	1054.430	1053.446	1099.451	1082.425	1081.441	1117.462	1116.478	133.043	1207.541	**1181.562**	**1164.535**	13	N
10	A	1142.494	1125.467	1124.483	1170.488	1153.462	1152.478	1188.499	1187.515	44.049	1320.625	**1294.646**	1277.619	12	I
11	N	1256.536	1239.510	1238.526	**1284.531**	1267.505	1266.521	1302.542	1301.558	87.055	1434.668	**1408.689**	**1391.662**	11	N
12	I	1369.621	1352.594	1351.610	1397.615	1380.589	1379.605	1415.626	1414.642	**86.096**	1505.705	**1479.726**	1462.699	10	A
13	N	1483.663	1466.637	1465.653	1511.658	1494.632	1493.648	1529.669	1528.685	87.055	1665.736	**1639.757**	1622.730	9	C*
14	I	1596.748	1579.721	1578.737	1624.742	1607.716	1606.732	1642.753	1641.769	**86.096**	1802.795	1776.816	1759.789	8	H
15	L	1709.832	1692.805	1691.821	1737.826	1720.800	1719.816	1755.837	1754.853	**86.096**	1899.848	**1873.868**	**1856.842**	7	P
16	D	1824.859	1807.832	1806.848	**1852.853**	1835.827	1834.843	1870.864	1869.880	88.039	1998.916	**1972.937**	**1955.910**	6	V
17	Y	1987.922	1970.895	1969.911	2015.917	1998.890	1997.906	2033.927	2032.943	**136.076**	2113.943	2087.964	**2070.937**	5	D
18	E	2116.964	2099.938	2098.954	2144.959	2127.933	2126.949	2162.970	2161.986	102.055	2210.996	**2185.016**	2167.990	4	P
19	V	2216.033	2199.006	2198.022	2244.028	2227.001	2226.017	2262.038	2261.054	72.081	2374.059	**2348.080**	2331.053	3	Y
20	C*	2376.063	2359.037	2358.053	2404.058	2387.032	2386.048	**2422.069**	2421.085	133.043	**2475.107**	**2449.127**	2432.101	2	T
21	R	**2532.165**	2515.138	2514.154	2560.160	2543.133	2542.149	**2578.170**	2577.186	129.113	2604.149	**2578.170**	2561.144	1	E

BioTools Version 3.2 Copyright © 1999-2008 Bruker Daltonik GmbH Page -2-

(**B**)

Figure S15. Annotated MS/MS spectrum of ion 2578.230 *m*/*z* detected in area #4. (**A**) MS/MS spectrum; (**B**) Peak list and amino acid sequence.

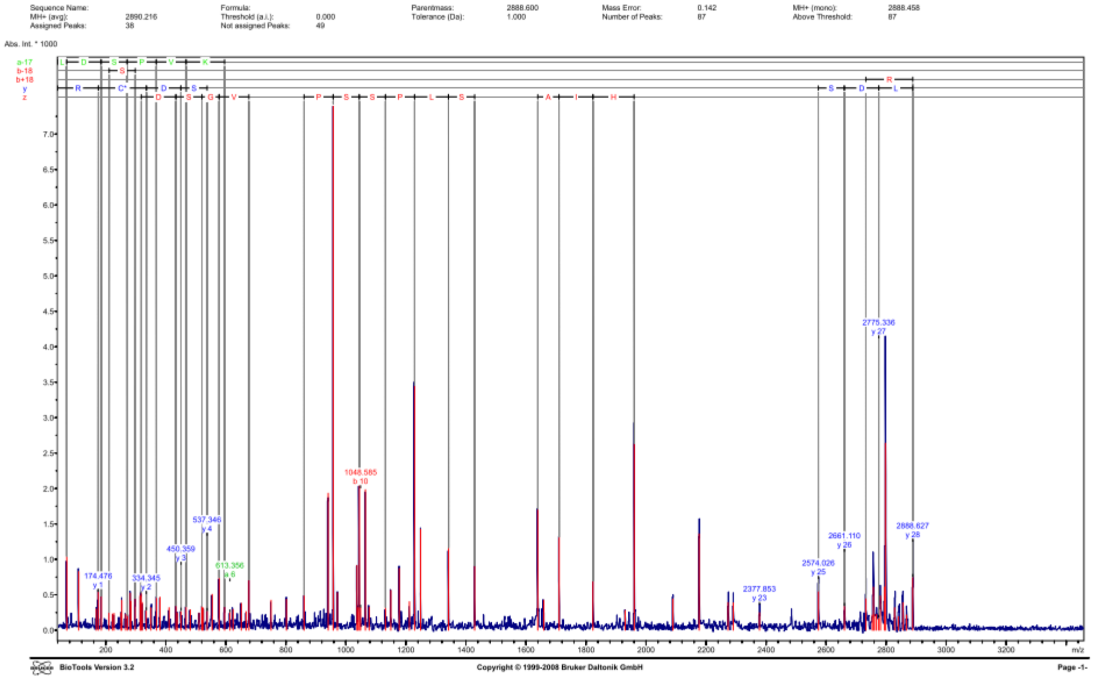

(**A**)

Display Parameter:

Parentmass:	2888.600	Mass Error:	0.142	MH+ (mono):	2888.458	MH+ (avg):	2890.216	Threshold (a.i.):	0.000
Tolerance (Da):	1.000	Number of Peaks:	87	Above Threshold:	87	Assigned Peaks:	38	Not assigned Peaks:	49

Peaklist:

Peak	Mass	Intensity	Peak	Mass	Intensity	Peak	Mass	Intensity	Peak	Mass	Intensity	Peak	Mass	Intensity	Peak	Mass	Intensity
1	69.612	1044.837	2	109.558	828.769	3	168.541	300.748	4	174.476	550.143	5	184.471	484.818	6	210.472	250.425
7	222.472	228.768	8	227.431	239.092	9	250.425	244.326	10	253.412	423.298	11	266.385	226.446	12	271.353	212.223
13	281.385	524.023	14	283.408	408.311	15	297.410	447.519	16	314.378	486.146	17	317.336	524.646	18	321.393	390.941
19	334.345	283.007	20	350.353	319.271	21	368.376	463.513	22	380.349	466.976	23	410.400	319.046	24	433.340	345.915
25	437.353	243.421	26	450.359	277.691	27	467.317	329.999	28	481.384	287.973	29	510.396	242.178	30	520.337	342.326
31	524.349	315.951	32	537.346	274.650	33	554.301	505.662	34	577.341	742.261	35	595.378	332.957	36	613.356	252.578
37	623.409	292.732	38	651.371	380.656	39	667.396	264.468	40	676.397	707.208	41	750.385	422.626	42	801.434	436.498
43	860.483	493.705	44	940.513	1933.697	45	957.524	7425.337	46	971.493	531.580	47	1036.590	914.950	48	1039.936	319.539
49	1044.549	2055.481	50	1048.585	320.021	51	1064.565	1984.184	52	1077.254	326.232	53	1131.635	327.267	54	1149.667	563.222
55	1177.643	874.001	56	1211.666	403.275	57	1228.669	3454.984	58	1248.686	1423.413	59	1341.736	1163.336	60	1428.802	909.491
61	1639.019	1705.566	62	1659.084	423.802	63	1710.089	1315.785	64	1823.209	692.845	65	1930.222	278.797	66	1960.310	2632.719
67	2089.412	441.665	68	2176.592	1359.398	69	2273.440	392.289	70	2290.482	386.149	71	2377.853	247.777	72	2574.026	554.621
73	2661.110	344.600	74	2732.276	456.891	75	2753.852	213.199	76	2757.245	608.095	77	2760.775	221.364	78	2768.008	221.940
79	2775.336	177.636	80	2780.429	484.841	81	2794.277	611.566	82	2797.649	2639.946	83	2828.100	318.485	84	2840.733	192.031
85	2855.644	390.067	86	2867.716	215.680	87	2888.627	753.201									

Calculated Masses:
LDSPVKNSAHIAPLSLPSSPPSVGSDCR 27: Carbamidomethyl (C)

N-Term.	Ion	a	a-17	a-18	b	b-17	b-18	b+18	c	i	x	y	z	C-Term.	Ion
1	L	86.096	**69.070**	68.086	114.091	97.065	96.081	132.102	131.118	86.096	201.098	**175.119**	158.092	28	R
2	D	201.123	**184.097**	183.113	229.118	212.092	**211.108**	247.129	246.145	88.039	361.129	**335.150**	**318.123**	27	C*
3	S	288.155	**271.129**	270.145	316.150	299.124	**298.140**	**334.161**	333.177	60.044	476.156	**450.177**	**433.150**	26	D
4	P	385.208	**368.182**	367.198	413.203	396.177	395.193	431.214	430.230	**70.065**	563.188	**537.209**	**520.182**	25	S
5	V	484.277	**467.250**	466.266	512.271	495.245	494.261	530.282	529.298	72.081	620.209	594.230	**577.203**	24	G
6	K	**612.372**	**595.345**	594.361	640.366	**623.340**	622.356	658.377	657.393	101.107	719.278	693.298	**676.272**	23	V
7	N	726.414	709.388	708.404	754.409	737.383	736.399	772.420	771.436	87.055	806.310	780.330	763.304	22	S
8	S	813.446	796.420	795.436	841.441	824.415	823.431	859.452	858.468	60.044	903.363	877.383	**860.357**	21	P
9	A	884.484	867.457	866.473	912.479	895.452	894.468	930.489	929.505	44.049	1000.415	974.436	**957.409**	20	P
10	H	1021.543	1004.516	1003.532	**1049.537**	1032.511	1031.527	1067.548	1066.564	**110.071**	1087.447	1061.468	**1044.441**	19	S
11	I	1134.627	1117.600	1116.616	1162.621	1145.595	1144.611	1180.632	1179.648	86.096	1174.479	1148.500	**1131.474**	18	S
12	A	1205.664	1188.637	1187.653	1233.659	1216.632	1215.648	1251.669	1250.685	44.049	1271.532	1245.553	**1228.526**	17	P
13	P	1302.716	1285.690	1284.706	1330.711	1313.685	1312.701	1348.722	1347.738	**70.065**	1384.616	1358.637	**1341.610**	16	L
14	L	1415.801	1398.774	1397.790	1443.795	1426.769	1425.785	1461.806	1460.822	86.096	1471.648	1445.669	**1428.642**	15	S
15	S	1502.833	1485.806	1484.822	1530.827	1513.801	1512.817	1548.838	1547.854	60.044	1584.732	1558.753	1541.726	14	L
16	L	1615.917	1598.890	1597.906	1643.912	1626.885	1625.901	1661.922	1660.938	86.096	1681.785	1655.806	**1638.779**	13	P
17	P	1712.969	1695.943	1694.959	1740.964	1723.938	1722.954	1758.975	1757.991	**70.065**	1752.822	1726.843	**1709.816**	12	A
18	S	1800.001	1782.975	1781.991	1827.996	1810.970	1809.986	1846.007	1845.023	60.044	1865.906	1839.927	**1822.900**	11	I
19	S	1887.033	1870.007	1869.023	1915.028	1898.002	1897.018	1933.039	1932.055	60.044	2002.965	1976.986	**1959.959**	10	H
20	P	1984.086	1967.060	1966.076	2012.081	1995.055	1994.071	2030.092	2029.108	**70.065**	2074.002	2048.023	2030.996	9	A
21	P	2081.139	2064.112	2063.128	2109.134	2092.107	2091.123	2127.144	2126.160	**70.065**	2161.034	2135.055	2118.028	8	S
22	S	2168.171	2151.144	2150.160	2196.166	2179.139	2178.155	2214.176	2213.192	60.044	2275.077	2249.098	2232.071	7	N
23	V	2267.239	2250.213	2249.229	2295.234	2278.208	2277.224	2313.245	2312.261	72.081	2403.172	**2377.193**	2360.166	6	K
24	G	2324.261	2307.234	2306.250	2352.256	2335.229	2334.245	2370.266	2369.282	30.034	2502.241	2476.261	2459.235	5	V
25	S	2411.293	2394.266	2393.282	2439.288	2422.261	2421.277	2457.298	2456.314	60.044	2599.293	**2573.314**	2556.287	4	P
26	D	2526.320	2509.293	2508.309	2554.315	2537.288	2536.304	2572.325	2571.341	88.039	2686.325	**2660.346**	2643.320	3	S
27	C*	2686.350	2669.324	2668.340	2714.345	2697.319	2696.335	**2732.356**	**2731.372**	133.043	2801.352	**2775.373**	2758.346	2	D
28	R	2842.452	2825.425	2824.441	2870.447	2853.420	2852.436	**2888.457**	2887.473	129.113	2914.436	**2888.457**	2871.431	1	L

BioTools Version 3.2 Copyright © 1999-2008 Bruker Daltonik GmbH Page -2-

(**B**)

Figure S16. Annotated MS/MS spectrum of ion 2888.600 *m/z* detected in area #4. (**A**) MS/MS spectrum; (**B**) Peak list and amino acid sequence.

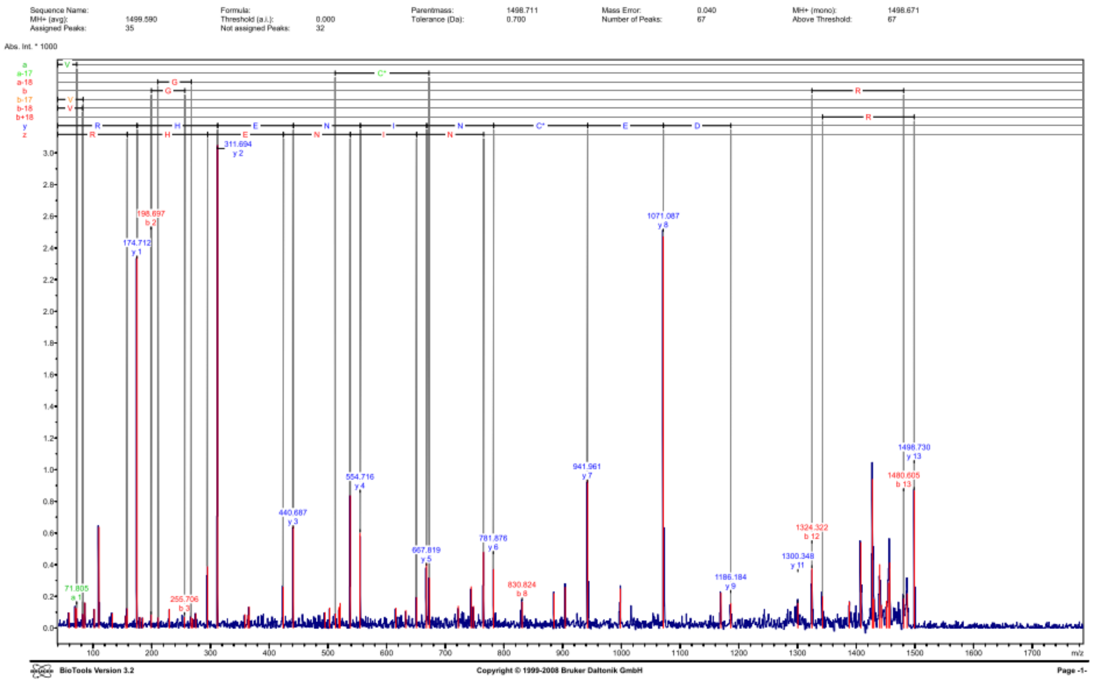

(**A**)

Display Parameter:

Parentmass: 1498.711 Mass Error: 0.040 MH+ (mono): 1498.671 MH+ (avg): 1499.590 Threshold (a.i.): 0.000
Tolerance (Da): 0.700 Number of Peaks: 67 Above Threshold: 67 Assigned Peaks: 35 Not assigned Peaks: 32

Peaklist:

Peak	Mass	Intensity	Peak	Mass	Intensity	Peak	Mass	Intensity	Peak	Mass	Intensity	Peak	Mass	Intensity	Peak	Mass	Intensity
1	58.812	95.958	2	69.780	129.891	3	71.805	126.555	4	82.371	90.899	5	85.794	159.395	6	101.761	118.402
7	109.743	636.892	8	132.676	90.692	9	155.681	75.012	10	157.690	127.502	11	174.712	2349.373	12	179.674	67.503
13	184.628	63.366	14	198.697	85.686	15	210.682	68.113	16	229.650	117.898	17	255.706	71.742	18	266.647	71.961
19	269.676	57.613	20	274.600	90.762	21	294.683	392.726	22	311.694	3051.575	23	358.646	83.924	24	363.612	79.396
25	365.662	133.442	26	423.690	266.178	27	440.687	645.732	28	494.676	97.430	29	502.702	126.684	30	512.683	96.237
31	518.579	130.019	32	520.677	155.766	33	537.693	840.275	34	554.716	605.425	35	615.750	125.058	36	632.664	111.319
37	650.779	198.326	38	667.819	409.160	39	672.818	318.974	40	721.801	137.597	41	743.865	260.592	42	747.796	136.987
43	764.842	481.463	44	781.876	377.488	45	830.824	163.807	46	884.958	217.629	47	903.950	257.446	48	941.961	936.006
49	998.088	246.822	50	1071.087	2479.055	51	1169.173	220.505	52	1186.184	158.093	53	1300.348	157.029	54	1324.322	378.673
55	1342.401	202.574	56	1387.957	164.193	57	1407.577	538.723	58	1427.361	939.009	59	1429.826	181.219	60	1439.895	400.223
61	1442.852	143.990	62	1451.294	169.531	63	1454.785	284.285	64	1456.833	411.418	65	1480.605	189.430	66	1486.335	215.236
67	1498.730	871.387															

Calculated Masses:

VVGGDECNINEHR 7: Carbamidomethyl (C)

N-Term.	Ion	a	a-17	a-18	b	b-17	b-18	b+18	c	i	x	y	z	C-Term.	Ion
1	V	**72.081**	55.054	54.070	100.076	**83.049**	**82.065**	118.086	117.102	**72.081**	201.098	**175.119**	**158.092**	13	R
2	V	171.149	154.123	153.139	**199.144**	182.118	181.134	217.155	216.171	**72.081**	338.157	**312.178**	**295.151**	12	H
3	G	228.171	**211.144**	**210.160**	**256.166**	239.139	238.155	**274.176**	273.192	30.034	467.200	**441.220**	**424.194**	11	E
4	G	285.192	268.166	**267.182**	313.187	296.160	**295.176**	331.198	330.214	30.034	581.243	**555.263**	**538.237**	10	N
5	D	400.219	383.193	382.208	428.214	411.187	410.203	446.225	445.241	88.039	694.327	**668.347**	**651.321**	9	I
6	E	529.262	**512.235**	511.251	557.257	540.230	539.246	575.267	574.283	**102.055**	808.370	**782.390**	**765.364**	8	N
7	C*	689.292	**672.266**	671.282	717.287	700.261	699.277	735.298	734.314	**133.043**	968.400	**942.421**	925.394	7	C*
8	N	803.335	786.309	785.325	**831.330**	814.304	813.320	849.341	848.357	87.055	1097.443	**1071.464**	1054.437	6	E
9	I	916.419	899.393	898.409	944.414	927.388	926.404	962.425	961.441	**86.096**	1212.470	**1186.491**	**1169.464**	5	D
10	N	1030.462	1013.436	1012.452	1058.457	1041.431	1040.447	1076.468	1075.484	87.055	1269.491	1243.512	1226.485	4	G
11	E	1159.505	1142.478	1141.494	1187.500	1170.473	**1169.489**	1205.510	1204.526	**102.055**	1326.513	**1300.533**	1283.507	3	G
12	H	1296.564	1279.537	1278.553	**1324.559**	1307.532	1306.548	**1342.569**	1341.585	**110.071**	1425.581	1399.602	1382.575	2	V
13	R	1452.665	1435.638	1434.654	**1480.660**	1463.633	1462.649	**1498.670**	1497.686	129.113	1524.650	**1498.670**	1481.644	1	V

BioTools Version 3.2 Copyright © 1999-2008 Bruker Daltonik GmbH Page -2-

(**B**)

Figure S17. Annotated MS/MS spectrum of ion 1498.711 *m*/*z* detected in area #5. (**A**) MS/MS spectrum; (**B**) Peak list and amino acid sequence.

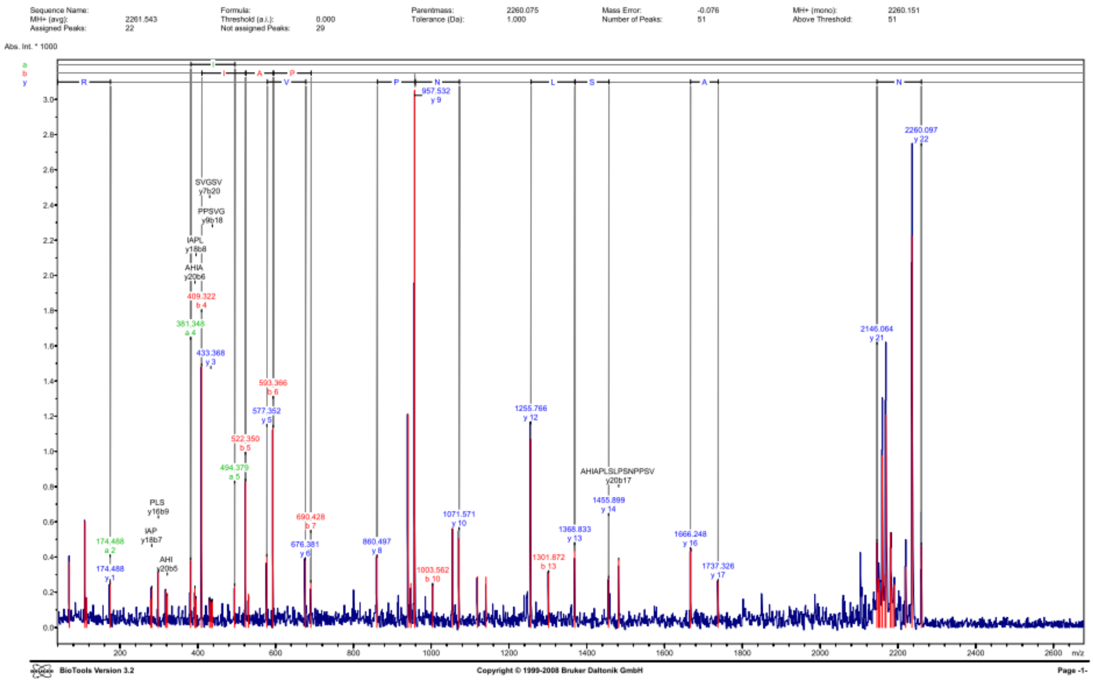

(**A**)

Display Parameter:

Parentmass:	2260.075	Mass Error:	-0.076	MH+ (mono):	2260.151	MH+ (avg):	2261.543	Threshold (a.i.):	0.000		
Tolerance (Da):	1.000	Number of Peaks:	51	Above Threshold:	51	Assigned Peaks:	22	Not assigned Peaks:	29		

Peaklist:

Peak	Mass	Intensity	Peak	Mass	Intensity	Peak	Mass	Intensity	Peak	Mass	Intensity	Peak	Mass	Intensity	Peak	Mass	Intensity
1	69.642	372.161	2	109.572	601.251	3	113.653	167.752	4	174.488	257.491	5	279.434	160.235	6	281.387	220.933
7	298.352	311.239	8	317.348	211.296	9	321.388	178.766	10	381.348	381.507	11	392.359	221.009	12	395.358	162.315
13	409.322	1482.901	14	430.431	156.503	15	433.368	143.919	16	437.343	146.558	17	494.379	234.062	18	522.350	828.708
19	530.436	189.889	20	577.352	400.903	21	593.366	1134.339	22	676.381	397.149	23	690.428	253.662	24	860.497	412.123
25	940.520	1211.176	26	947.554	251.150	27	957.532	3140.404	28	1003.562	239.730	29	1054.571	564.723	30	1071.571	508.682
31	1118.660	289.592	32	1140.675	286.479	33	1255.766	1073.871	34	1301.872	323.498	35	1368.833	434.444	36	1455.899	276.728
37	1481.994	380.625	38	1666.248	442.547	39	1737.326	261.889	40	2146.064	484.333	41	2150.710	279.743	42	2154.088	210.197
43	2159.692	975.778	44	2166.861	301.439	45	2169.162	1209.387	46	2180.794	535.641	47	2184.987	224.302	48	2190.110	247.268
49	2219.771	343.718	50	2236.237	2223.892	51	2260.097	465.331									

Calculated Masses:
NSAHIAPLSLPSNPPSVGSVCR 21: Carbamidomethyl (C)

N-Term.	Ion	a	b	y	C-Term.	Ion
1	N	87.055	115.050	**175.119**	22	R
2	S	**174.087**	202.082	335.150	21	C*
3	A	245.124	273.119	**434.218**	20	V
4	H	**382.183**	**410.178**	521.250	19	S
5	I	**495.267**	**523.262**	**578.272**	18	G
6	A	566.305	**594.299**	**677.340**	17	V
7	P	663.357	**691.352**	764.372	16	S
8	L	776.441	804.436	**861.425**	15	P
9	S	863.473	891.468	**958.477**	14	P
10	L	976.557	**1004.552**	**1072.520**	13	N
11	P	1073.610	1101.605	1159.552	12	S
12	S	1160.642	1188.637	**1256.605**	11	P
13	N	1274.685	**1302.680**	**1369.689**	10	L
14	P	1371.738	1399.733	**1456.721**	9	S
15	P	1468.791	1496.786	1569.805	8	L
16	S	1555.823	1583.818	**1666.858**	7	P
17	V	1654.891	1682.886	**1737.895**	6	A
18	G	1711.913	1739.908	1850.979	5	I
19	S	1798.945	1826.940	1988.038	4	H
20	V	1898.013	1926.008	2059.075	3	A
21	C*	2058.044	2086.039	**2146.107**	2	S
22	R	2214.145	2242.140	**2260.150**	1	N

BioTools Version 3.2 Copyright © 1999-2008 Bruker Daltonik GmbH Page -2-

(**B**)

Figure S18. Annotated MS/MS spectrum of ion 2260.075 *m*/*z* detected in area #5. (**A**) MS/MS spectrum. (**B**) Peak list and amino acid sequence.

Sequence Name: Protein C activator OS=Agkistrodon contortrix contortrix PE=1 SV=2 VSPCA_AGKCO
Formula:
Parentmass:
Mass Error: MH+ (mono): 1.008 MH+ (avg): 1.008 Threshold (a.i.): 0.000 Tolerance (Da): 0.500
Number of Peaks: 13 Above Threshold: Assigned Peaks: Not assigned Peaks:

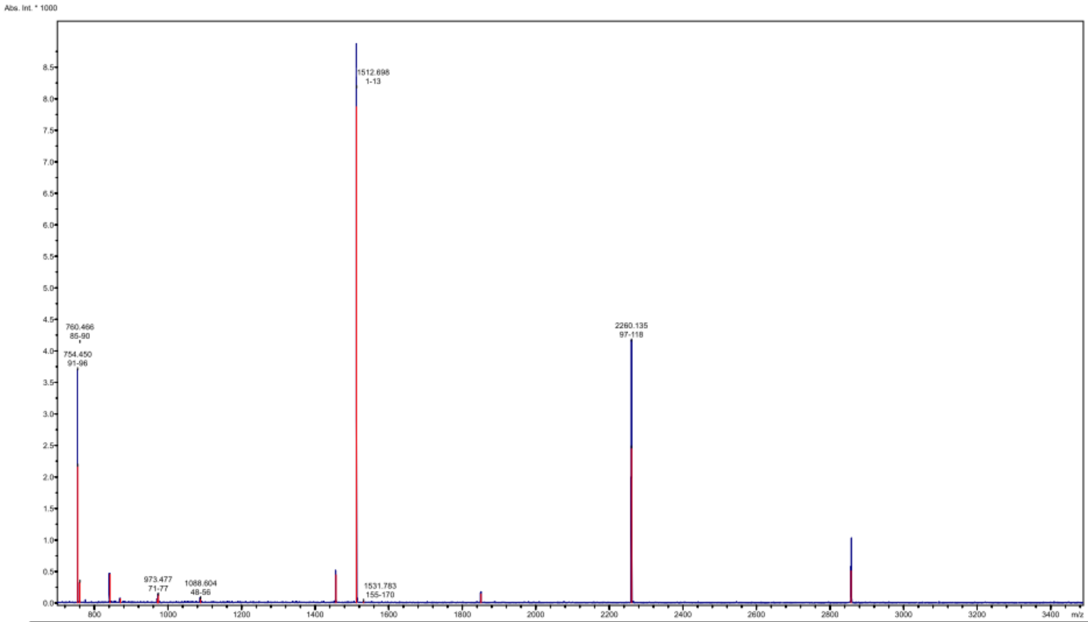

BioTools Version 3.2 Copyright © 1999-2008 Bruker Daltonik GmbH Page -1-

(A)

Sequence data:

Protein C activator OS=Agkistrodon contortrix contortrix PE=1 SV=2 VSPCA_AGKCO

Intensity Coverage: 88.1 % (12970 cnts) Sequence Coverage MS: 34.2%
Sequence Coverage MS/MS: 0.0% pI (isoelectric point): 9.7

10	20	30	40	50	60	70	80	90	100	110
VIGGDECNIN	EHRFLALVYA	NGSLCGGTLI	NQEWVLTARH	CDRGNMRIYL	GMHNLKVLNK	DALRRFPKEK	YFCLNTRNDT	IWDKDIMLIR	LNRPVRNSAH	IAPLSLPSNP
**120**	**130**	**140**	**150**	**160**	**170**	**180**	**190**	**200**	**210**	**220**
PSVGSVCRIM	GWGTITSPNA	TLPDVPHCAN	INILDYAVCQ	AAYKGLAATT	LCAGILEGGK	DTCKGDSGGP	LICNGQFQGI	LSVGGNPCAQ	PRKPGIYTKV	FDYTDWIQSI
**230**	**240**									
ISGNTDATCP	P									

Display Parameter:

Sequence Name: Protein C activator OS=Agkistrodon contortrix contortrix PE=1 SV=2 VSPCA_AGKCO MH+ (mono): 1.008 MH+ (avg): 1.008
Threshold (a.i.): 0.000 Tolerance (Da): 0.500 Number of Peaks: 13

Peaklist:

Peak	Mass	Intensity	Peak	Mass	Intensity	Peak	Mass	Intensity	Peak	Mass	Intensity	Peak	Mass	Intensity	Peak	Mass	Intensity
1	754.450	2164.000	2	760.466	324.000	3	842.523	453.716	4	870.559	65.345	5	971.567	60.437	6	973.477	127.624
7	1088.604	70.228	8	1456.740	446.727	9	1512.698	7873.273	10	1531.783	27.000	11	1850.978	142.599	12	2260.135	2451.404
13	2857.311	510.887															

BioTools Version 3.2 Copyright © 1999-2008 Bruker Daltonik GmbH Page -2-

(B)

Figure S19. Annotated MS spectrum detected in area #5. (**A**) MS spectrum; (**B**) Peak list and sequence data.

Sequence Name: Basic phospholipase A2 homolog OS=Agkistrodon piscivorus piscivorus PE=1 SV=1 PA2HB_AGKPI
Formula:
Parentmass:
Mass Error:
MH+ (mono): 1.008
MH+ (avg): 1.008
Threshold (a.i.): 0.000
Tolerance (Da): 0.500
Number of Peaks: 17
Above Threshold:
Assigned Peaks:
Not assigned Peaks:

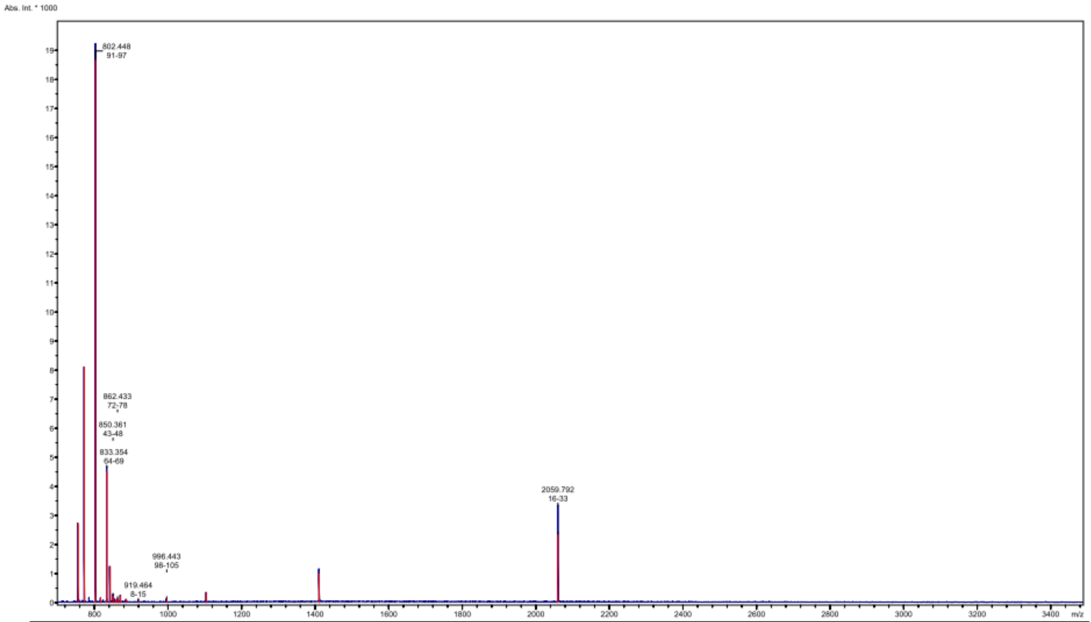

BioTools Version 3.2 Copyright © 1999-2008 Bruker Daltonik GmbH Page -1-

(**A**)

Sequence data:

Basic phospholipase A2 homolog OS=Agkistrodon piscivorus piscivorus PE=1 SV=1 PA2HB_AGKPI

Intensity Coverage: 64.9 % (26086 cnts)
Sequence Coverage MS/MS: 0.0%
Sequence Coverage MS: 49.6%
pI (isoelectric point): 10.0

```
         10         20         30         40         50         60         70         80         90        100        110
SVLELGKMIL QETGKNAITS YGSYGCNCGW GHRGQPKDAT DRCCFVHKCC YKKLTDCNHK TDRYSYSWKN KAIICEEKNP CLKEMCECDK AVAICLRENL DTYNKKYKAY
        120        130
FKLKCKKPDT C
```

Display Parameter:

Sequence Name: Basic phospholipase A2 homolog OS=Agkistrodon piscivorus piscivorus PE=1 SV=1 PA2HB_AGKPI
MH+ (mono): 1.008
MH+ (avg): 1.008
Threshold (a.i.): 0.000
Tolerance (Da): 0.500
Number of Peaks: 17

Peaklist:

Peak	Mass	Intensity	Peak	Mass	Intensity	Peak	Mass	Intensity	Peak	Mass	Intensity	Peak	Mass	Intensity	Peak	Mass	Intensity
1	755.288	2729.000	2	772.314	8109.000	3	802.448	18674.538	4	816.459	146.171	5	833.354	4506.474	6	842.496	1231.694
7	850.361	234.526	8	856.519	114.431	9	862.433	124.000	10	865.364	88.000	11	870.534	234.583	12	886.376	115.000
13	919.464	75.000	14	996.443	125.000	15	1103.413	335.107	16	1410.520	1006.110	17	2059.792	2346.739			

BioTools Version 3.2 Copyright © 1999-2008 Bruker Daltonik GmbH Page -2-

(**B**)

Figure S20. Annotated MS spectrum detected in area #6. (**A**) MS spectrum; (**B**) Peak list and sequence data.

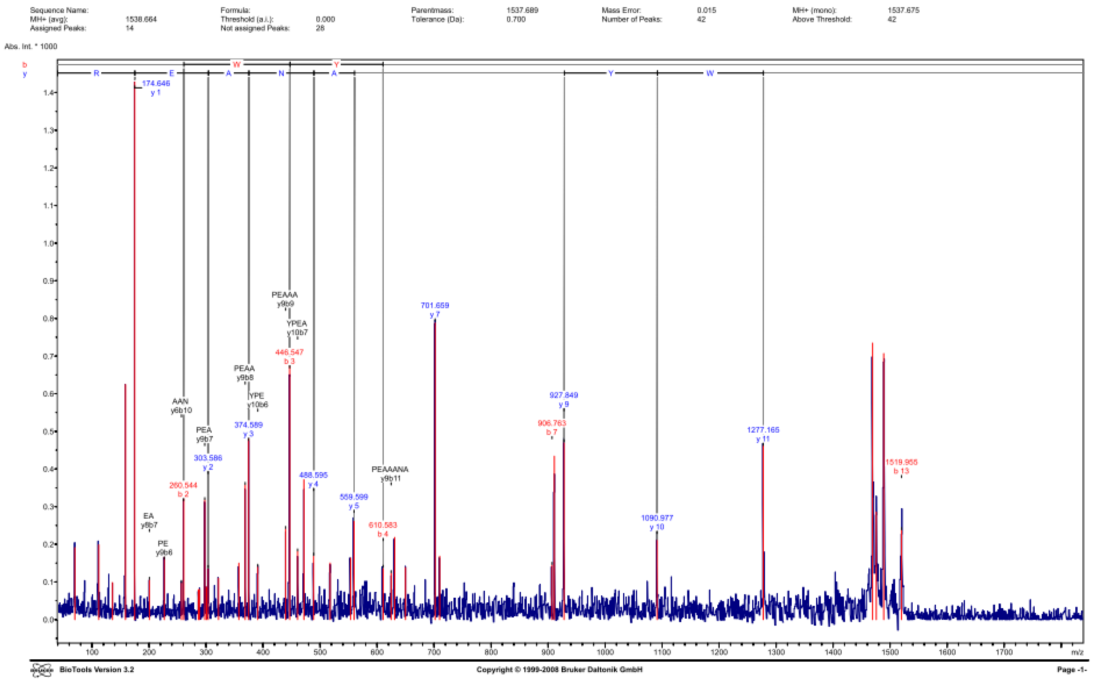

(**A**)

Display Parameter:

Parentmass: 1537.689 Mass Error: 0.015 MH+ (mono): 1537.675 MH+ (avg): 1538.664 Threshold (a.i.): 0.000
Tolerance (Da): 0.700 Number of Peaks: 42 Above Threshold: 42 Assigned Peaks: 14 Not assigned Peaks: 28

Peaklist:

Peak	Mass	Intensity	Peak	Mass	Intensity	Peak	Mass	Intensity	Peak	Mass	Intensity	Peak	Mass	Intensity	Peak	Mass	Intensity
1	69.736	191.706	2	111.712	199.027	3	135.665	98.715	4	158.634	624.835	5	174.646	1434.016	6	200.552	105.650
7	226.596	166.881	8	256.613	97.210	9	260.544	321.461	10	286.561	79.908	11	288.547	80.255	12	297.570	317.376
13	300.565	86.750	14	303.586	136.642	15	321.596	110.425	16	357.551	150.069	17	368.564	357.901	18	374.589	481.942
19	390.577	140.414	20	439.563	241.021	21	446.547	674.706	22	460.571	181.084	23	471.563	373.817	24	488.595	169.667
25	517.540	150.616	26	553.548	163.703	27	559.599	262.805	28	610.583	137.552	29	624.608	123.420	30	630.607	218.757
31	649.632	138.638	32	701.659	768.346	33	709.640	168.762	34	906.763	145.267	35	910.778	434.375	36	927.849	471.356
37	1090.977	212.287	38	1277.165	468.729	39	1469.146	734.927	40	1475.690	286.271	41	1488.484	707.030	42	1519.955	237.926

Calculated Masses:
MEWYPEAAANAER

N-Term.	Ion	a	b	y	C-Term.	Ion
1	M	104.053	132.048	**175.119**	13	R
2	E	233.095	**261.090**	**304.162**	12	E
3	W	419.175	**447.170**	**375.199**	11	A
4	Y	582.238	**610.233**	**489.242**	10	N
5	P	679.291	707.286	**560.279**	9	A
6	E	808.333	836.328	631.316	8	A
7	A	879.371	**907.365**	**702.353**	7	A
8	A	950.408	978.403	831.396	6	E
9	A	1021.445	1049.440	**928.448**	5	P
10	N	1135.488	1163.483	**1091.512**	4	Y
11	A	1206.525	1234.520	**1277.591**	3	W
12	E	1335.567	1363.562	1406.634	2	E
13	R	1491.669	**1519.663**	1537.674	1	M

BioTools Version 3.2 Copyright © 1999-2008 Bruker Daltonik GmbH Page -2-

(**B**)

Figure S21. Annotated MS/MS spectrum of ion 1537.689 *m*/*z* detected in area #7. (**A**) MS/MS spectrum; (**B**) Peak list and amino acid sequence.

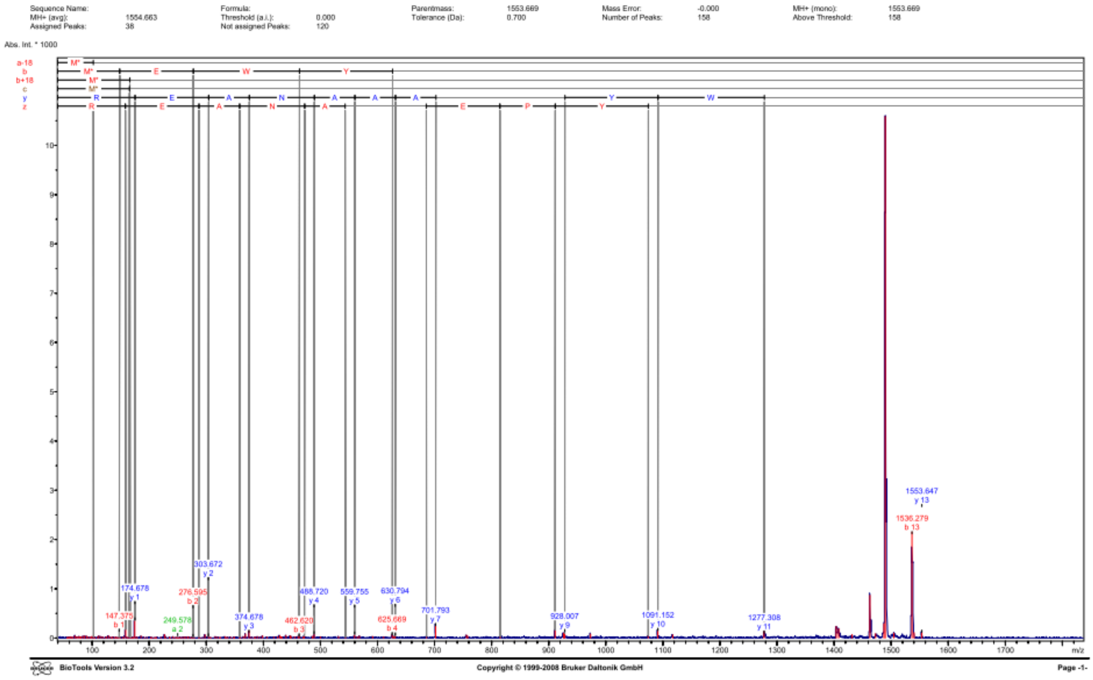

(**A**)

Display Parameter:

Parentmass:	1553.669	Mass Error:	-0.000	MH+ (mono):	1553.669	MH+ (avg):	1554.663	Threshold (a.i.):	0.000
Tolerance (Da):	0.700	Number of Peaks:	158	Above Threshold:	158	Assigned Peaks:	38	Not assigned Peaks:	120

Peaklist:

Peak	Mass	Intensity	Peak	Mass	Intensity	Peak	Mass	Intensity	Peak	Mass	Intensity	Peak	Mass	Intensity	Peak	Mass	Intensity
1	55.800	11.094	2	57.492	10.683	3	58.720	15.123	4	60.664	19.673	5	62.441	24.144	6	64.833	17.476
7	66.168	10.261	8	69.715	45.893	9	72.840	21.158	10	74.870	24.067	11	77.373	31.301	12	78.976	23.409
13	80.651	19.814	14	83.575	32.995	15	86.658	40.660	16	90.257	38.085	17	93.243	13.159	18	95.782	17.230
19	98.343	21.440	20	99.754	33.265	21	102.319	20.607	22	104.762	11.792	23	109.717	22.099	24	111.734	41.734
25	115.678	27.685	26	118.274	20.355	27	122.262	9.827	28	124.795	25.457	29	128.715	42.947	30	132.369	17.573
31	134.488	9.767	32	135.713	19.677	33	138.729	15.174	34	141.289	12.358	35	142.717	16.705	36	145.481	6.897
37	147.375	11.675	38	149.174	11.417	39	153.646	15.116	40	155.088	12.429	41	158.684	175.413	42	161.524	13.912
43	164.728	22.511	44	166.665	12.548	45	168.621	12.493	46	170.562	12.494	47	171.898	12.013	48	174.678	359.673
49	177.227	12.864	50	178.950	14.898	51	185.658	40.393	52	191.177	16.360	53	195.716	11.130	54	202.228	12.058
55	203.500	10.623	56	205.438	7.424	57	209.073	8.884	58	210.853	8.113	59	212.671	13.426	60	220.539	15.382
61	226.648	72.131	62	229.534	10.421	63	235.157	13.672	64	236.624	10.027	65	241.678	25.251	66	244.606	9.004
67	247.202	14.454	68	249.578	9.804	69	251.013	7.034	70	253.764	15.470	71	256.653	9.313	72	258.621	17.847
73	261.995	16.626	74	264.370	18.376	75	267.636	25.078	76	270.618	16.352	77	273.675	11.923	78	276.595	20.686
79	278.977	8.787	80	286.617	24.759	81	288.951	8.012	82	293.271	7.557	83	297.639	67.814	84	303.672	36.740
85	305.888	7.818	86	307.757	10.596	87	315.640	27.349	88	319.191	17.810	89	323.167	10.367	90	324.553	10.398
91	332.867	27.450	92	335.746	10.017	93	337.602	14.268	94	342.658	16.686	95	347.635	12.171	96	349.136	19.502
97	358.033	22.918	98	359.718	22.015	99	364.984	29.504	100	368.650	85.193	101	374.678	161.409	102	378.918	13.859
103	384.209	12.683	104	385.686	15.531	105	389.636	28.806	106	394.445	13.183	107	398.649	45.133	108	406.614	20.304
109	418.228	21.482	110	424.877	25.746	111	428.711	50.970	112	430.628	25.463	113	432.966	18.433	114	439.673	53.983
115	446.670	46.231	116	448.625	30.127	117	454.671	25.819	118	460.678	31.091	119	462.620	44.660	120	469.246	21.154
121	471.661	52.537	122	485.787	31.483	123	488.720	82.815	124	499.720	25.329	125	531.690	30.382	126	543.694	20.882
127	559.755	73.555	128	564.503	23.026	129	568.277	39.777	130	591.441	31.556	131	610.655	23.877	132	625.669	72.664
133	628.760	29.503	134	630.794	48.683	135	685.829	32.817	136	701.793	299.013	137	755.807	59.949	138	757.829	34.112
139	814.833	33.157	140	910.935	162.083	141	926.020	101.491	142	928.007	92.324	143	973.113	78.956	144	1074.044	48.339
145	1091.152	156.358	146	1117.177	65.137	147	1277.308	146.028	148	1403.559	235.019	149	1407.964	188.540	150	1431.431	70.935
151	1462.549	869.450	152	1472.633	88.957	153	1487.428	291.560	154	1489.631	10586.258	155	1504.737	100.457	156	1536.279	2162.022
157	1540.109	108.759	158	1553.647	109.696												

Calculated Masses:
MEWYPEAAAANAER 1: Oxidation (M)

N-Term.	Ion	a	a-17	a-18	b	b-17	b-18	b+18	c	i	x	y	z	C-Term.	Ion
1	M*	120.048	103.021	**102.037**	**148.043**	131.016	130.032	**166.053**	**165.069**	120.048	201.098	**175.119**	**158.092**	13	R
2	E	**249.090**	232.064	231.080	**277.085**	260.059	**259.075**	295.096	294.112	**102.055**	330.141	**304.162**	**287.135**	12	E
3	W	435.170	**418.143**	417.159	**463.165**	**446.138**	445.154	481.175	480.191	**159.092**	401.178	**375.199**	**358.172**	11	A
4	Y	598.233	581.206	580.222	**626.228**	609.201	608.217	644.238	643.254	**136.076**	515.221	**489.242**	**472.215**	10	N
5	P	695.286	678.259	677.275	723.281	706.254	705.270	741.291	740.307	**70.065**	586.258	**560.279**	**543.252**	9	A
6	E	824.328	807.302	806.318	852.323	835.297	834.313	870.334	869.350	**102.055**	657.295	**631.316**	614.289	8	A
7	A	895.365	878.339	877.355	923.360	906.334	905.350	941.371	940.387	44.049	728.332	**702.353**	**685.326**	7	A
8	A	966.403	949.376	948.392	994.397	977.371	976.387	1012.408	1011.424	44.049	857.375	831.396	**814.369**	6	E
9	A	1037.440	1020.413	1019.429	1065.435	1048.408	1047.424	1083.445	1082.461	44.049	954.428	**928.448**	**911.422**	5	P
10	N	1151.483	1134.456	1133.472	1179.478	1162.451	1161.467	1197.488	1196.504	**87.055**	**1117.491**	**1091.512**	**1074.485**	4	Y
11	A	1222.520	1205.493	1204.509	1250.515	1233.488	1232.504	1268.525	1267.541	44.049	1303.570	**1277.591**	1260.564	3	W
12	E	1351.562	1334.536	1333.552	1379.557	1362.531	1361.547	1397.568	1396.584	**102.055**	1432.613	1406.634	1389.607	2	E
13	R	1507.663	1490.637	**1489.653**	**1535.658**	1518.632	1517.648	**1553.669**	1552.685	**129.113**	1579.648	**1553.669**	**1536.642**	1	M*

BioTools Version 3.2 Copyright © 1999-2008 Bruker Daltonik GmbH Page -2-

(**B**)

Figure S22. Annotated MS/MS spectrum of ion 1553.669 *m*/*z* detected in area #7. (**A**) MS/MS spectrum; (**B**) Peak list and amino acid sequence.

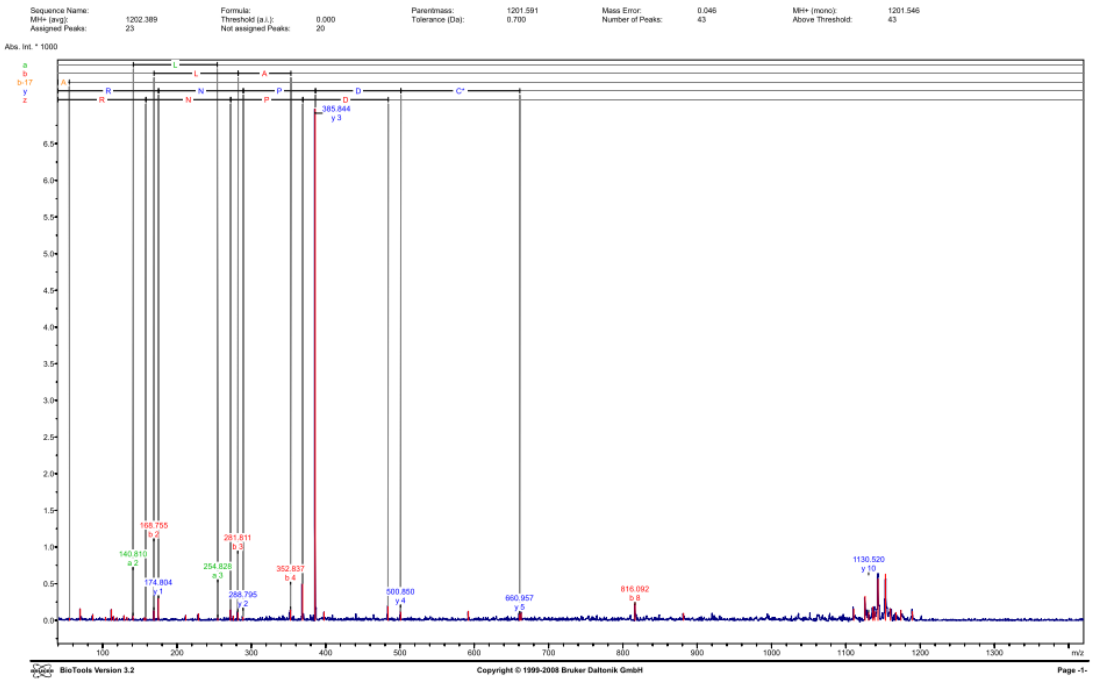

(**A**)

Display Parameter:

Parentmass: 1201.591 Mass Error: 0.046 MH+ (mono): 1201.546 MH+ (avg): 1202.389 Threshold (a.i.): 0.000
Tolerance (Da): 0.700 Number of Peaks: 43 Above Threshold: 43 Assigned Peaks: 23 Not assigned Peaks: 20

Peaklist:

Peak	Mass	Intensity	Peak	Mass	Intensity	Peak	Mass	Intensity	Peak	Mass	Intensity	Peak	Mass	Intensity	Peak	Mass	Intensity
1	55.332	40.287	2	69.853	155.321	3	86.824	76.311	4	103.780	40.076	5	111.804	145.963	6	114.832	51.141
7	118.987	33.037	8	128.806	63.344	9	132.755	40.125	10	140.810	63.271	11	155.952	31.470	12	157.788	45.820
13	168.755	137.183	14	174.804	308.905	15	211.793	66.205	16	228.754	88.692	17	254.828	39.064	18	271.770	152.387
19	275.703	57.990	20	281.811	113.565	21	288.795	53.373	22	352.837	149.384	23	368.829	501.592	24	385.844	6996.125
25	397.864	113.645	26	483.867	203.052	27	500.850	124.333	28	592.021	123.968	29	660.957	94.361	30	663.031	105.022
31	816.092	245.580	32	881.485	96.141	33	1110.452	153.185	34	1125.797	327.988	35	1130.520	106.004	36	1136.407	182.011
37	1138.584	117.748	38	1143.198	564.451	39	1153.182	630.921	40	1159.909	155.270	41	1167.271	106.782	42	1174.149	138.063
43	1189.495	119.986															

Calculated Masses:

APLAGMCDPNR 7: Carbamidomethyl (C)

N-Term.	Ion	a	a-17	a-18	b	b-17	b-18	b+18	c	i	x	y	z	C-Term.	Ion
1	A	44.049	27.023	26.039	72.044	**55.018**	54.034	90.055	89.071	44.049	201.098	**175.119**	**158.092**	11	R
2	P	**141.102**	124.076	123.092	**169.097**	152.071	151.087	187.108	186.124	**70.065**	315.141	**289.162**	**272.135**	10	N
3	L	**254.186**	237.160	236.176	**282.181**	265.155	264.171	300.192	299.208	86.096	412.194	**386.215**	**369.188**	9	P
4	A	325.223	308.197	307.213	**353.218**	336.192	335.208	371.229	370.245	44.049	527.221	**501.242**	**484.215**	8	D
5	G	382.245	365.218	364.234	410.240	393.213	392.229	428.250	427.266	30.034	687.251	**661.272**	644.246	7	C*
6	M	513.285	496.259	495.275	541.280	524.254	523.270	559.291	558.307	**104.053**	818.292	792.313	775.286	6	M
7	C*	673.316	656.289	655.305	701.311	684.284	683.300	719.321	718.337	**133.043**	875.313	849.334	832.308	5	G
8	D	788.343	771.316	770.332	**816.338**	799.311	798.327	834.348	833.364	88.039	946.351	920.371	903.345	4	A
9	P	885.396	868.369	867.385	913.391	896.364	895.380	931.401	930.417	**70.065**	1059.435	1033.455	1016.429	3	L
10	N	999.439	982.412	981.428	1027.434	1010.407	1009.423	1045.444	1044.460	**87.055**	1156.487	**1130.508**	1113.482	2	P
11	R	1155.540	**1138.513**	1137.529	1183.535	1166.508	1165.524	1201.545	1200.561	**129.113**	1227.525	1201.545	1184.519	1	A

BioTools Version 3.2 Copyright © 1999-2008 Bruker Daltonik GmbH Page -2-

(**B**)

Figure S23. Annotated MS/MS spectrum of ion 1201.591 *m*/*z* detected in area #8. (**A**) MS/MS spectrum; (**B**) Peak list and amino acid sequence.

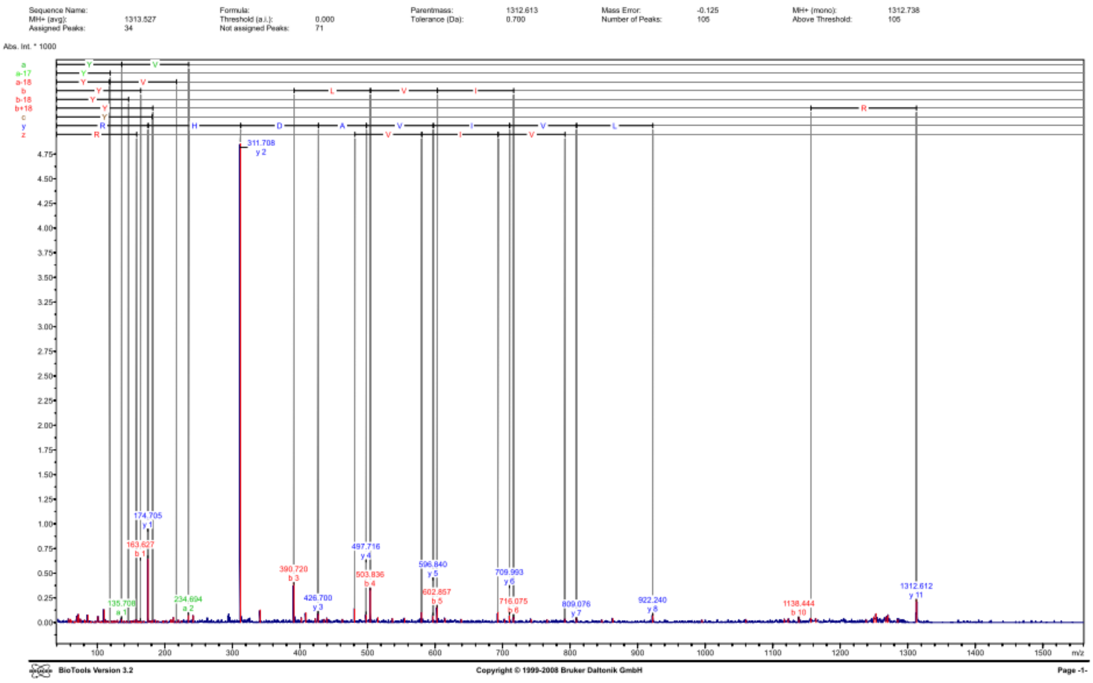

(**A**)

Display Parameter:

Parentmass: 1312.613 Mass Error: -0.125 MH+ (mono): 1312.738 MH+ (avg): 1313.527 Threshold (a.i.): 0.000
Tolerance (Da): 0.700 Number of Peaks: 105 Above Threshold: 105 Assigned Peaks: 34 Not assigned Peaks: 71

Peaklist:

Peak	Mass	Intensity	Peak	Mass	Intensity	Peak	Mass	Intensity	Peak	Mass	Intensity	Peak	Mass	Intensity	Peak	Mass	Intensity
1	57.355	35.651	2	59.760	31.986	3	61.397	19.103	4	65.311	7.928	5	67.015	7.784	6	69.819	59.837
7	71.797	82.821	8	74.986	33.397	9	80.640	23.148	10	82.877	13.111	11	85.766	74.218	12	89.430	8.233
13	90.838	8.532	14	94.953	7.392	15	97.246	10.450	16	100.751	59.315	17	103.752	6.956	18	105.427	9.072
19	106.687	9.481	20	109.714	129.378	21	112.652	19.609	22	114.423	14.711	23	116.851	10.151	24	118.757	5.929
25	121.023	13.710	26	122.773	12.589	27	124.818	18.454	28	126.771	14.570	29	129.808	21.398	30	135.708	39.724
31	139.766	16.711	32	142.736	12.679	33	145.736	14.152	34	149.558	16.313	35	152.256	14.360	36	154.683	20.793
37	157.680	35.231	38	160.299	9.912	39	161.588	9.607	40	163.627	14.212	41	165.608	8.796	42	168.721	12.323
43	174.705	649.691	44	178.073	14.303	45	180.301	17.442	46	181.643	30.149	47	185.726	24.273	48	189.921	16.275
49	197.090	16.518	50	198.384	14.239	51	202.488	19.190	52	208.701	26.877	53	210.707	26.821	54	212.714	52.102
55	216.840	14.117	56	219.935	23.575	57	224.713	16.814	58	234.694	80.752	59	241.690	69.498	60	311.708	4878.544
61	340.756	126.694	62	390.720	406.548	63	401.860	47.590	64	408.683	99.417	65	422.729	38.784	66	426.700	93.409
67	435.372	29.714	68	439.841	42.120	69	462.774	30.952	70	480.716	144.092	71	497.716	82.304	72	503.836	352.751
73	515.120	48.129	74	536.779	39.637	75	554.815	38.351	76	579.838	107.914	77	596.840	70.156	78	602.857	174.989
79	614.581	39.246	80	638.717	31.027	81	692.904	110.175	82	702.943	29.169	83	709.993	76.948	84	716.075	59.710
85	742.095	30.968	86	765.956	26.531	87	791.987	38.731	88	809.076	52.352	89	847.017	23.485	90	863.040	38.978
91	922.240	94.181	92	995.179	23.698	93	1060.312	26.523	94	1117.098	31.873	95	1122.585	35.667	96	1138.444	58.771
97	1156.475	52.279	98	1164.025	39.368	99	1238.261	25.021	100	1249.855	57.686	101	1252.835	88.791	102	1267.538	55.403
103	1270.559	72.042	104	1284.949	28.549	105	1312.612	235.868									

Calculated Masses:
YVQLVIVADHR

N-Term.	Ion	a	a-17	a-18	b	b-17	b-18	b+18	c	i	x	y	z	C-Term.	Ion
1	Y	136.076	119.049	118.065	164.071	147.044	146.060	182.081	181.097	136.076	201.098	175.119	158.092	11	R
2	V	235.144	218.118	217.134	263.139	246.112	245.128	281.150	280.166	72.081	338.157	312.178	295.151	10	H
3	Q	363.203	346.176	345.192	391.198	374.171	373.187	409.208	408.224	101.071	453.184	427.205	410.178	9	D
4	L	476.287	459.260	458.276	504.282	487.255	486.271	522.292	521.308	86.096	524.221	498.242	481.215	8	A
5	V	575.355	558.329	557.345	603.350	586.324	585.340	621.361	620.377	72.081	623.290	597.310	580.284	7	V
6	I	688.439	671.413	670.429	716.434	699.408	698.424	734.445	733.461	86.096	736.374	710.394	693.368	6	I
7	V	787.508	770.481	769.497	815.503	798.476	797.492	833.513	832.529	72.081	835.442	809.463	792.436	5	V
8	A	858.545	841.518	840.534	886.540	869.513	868.529	904.550	903.566	44.049	948.526	922.547	905.520	4	L
9	D	973.572	956.545	955.561	1001.567	984.540	983.556	1019.577	1018.593	88.039	1076.585	1050.605	1033.579	3	Q
10	H	1110.631	1093.604	1092.620	1138.626	1121.599	1120.615	1156.636	1155.652	110.071	1175.653	1149.674	1132.647	2	V
11	R	1266.732	1249.705	1248.721	1294.727	1277.700	1276.716	1312.737	1311.753	129.113	1338.716	1312.737	1295.711	1	Y

BioTools Version 3.2 Copyright © 1999-2008 Bruker Daltonik GmbH Page -2-

(**B**)

Figure S24. Annotated MS/MS spectrum of ion 1312.613 *m*/*z* detected in area #8. (**A**) MS/MS spectrum; (**B**) Peak list and amino acid sequence.

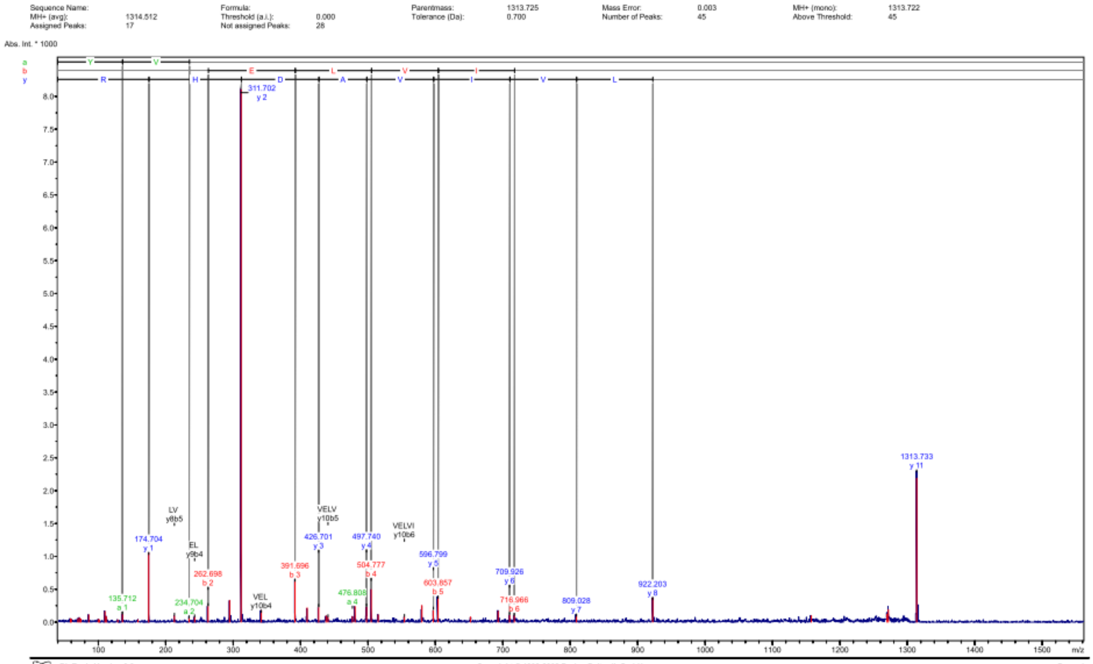

BioTools Version 3.2 Copyright © 1999-2008 Bruker Daltonik GmbH Page -1-

(**A**)

Display Parameter:

Parentmass: 1313.725 Mass Error: 0.003 MH+ (mono): 1313.722 MH+ (avg): 1314.512 Threshold (a.i.): 0.000
Tolerance (Da): 0.700 Number of Peaks: 45 Above Threshold: 45 Assigned Peaks: 17 Not assigned Peaks: 28

Peaklist:

Peak	Mass	Intensity	Peak	Mass	Intensity	Peak	Mass	Intensity	Peak	Mass	Intensity	Peak	Mass	Intensity	Peak	Mass	Intensity
1	58.667	53.727	2	59.918	43.812	3	69.771	50.542	4	71.800	67.062	5	74.201	47.544	6	85.770	112.165
7	99.744	40.167	8	101.744	35.559	9	109.732	163.055	10	111.702	82.882	11	128.746	36.538	12	135.712	125.324
13	158.711	34.697	14	174.704	1061.481	15	212.721	96.064	16	234.704	97.346	17	242.710	72.461	18	262.698	244.703
19	294.682	332.370	20	311.702	8116.495	21	341.720	182.961	22	391.696	655.716	23	409.687	219.057	24	426.701	231.012
25	437.662	95.141	26	440.742	75.216	27	476.808	84.024	28	480.719	239.688	29	497.740	236.284	30	504.777	507.900
31	515.126	112.365	32	553.842	80.067	33	579.802	253.996	34	596.799	190.140	35	603.857	396.149	36	651.904	78.939
37	692.943	166.112	38	709.926	115.382	39	716.966	104.519	40	809.028	123.612	41	922.203	377.187	42	1157.438	97.621
43	1269.717	123.071	44	1271.825	190.902	45	1313.733	2190.939									

Calculated Masses:
YVELVIVADHR

N-Term.	Ion	a	b	y	C-Term.	Ion
1	Y	**136.076**	164.071	**175.119**	11	R
2	V	**235.144**	**263.139**	**312.178**	10	H
3	E	364.187	**392.182**	**427.205**	9	D
4	L	**477.271**	**505.266**	**498.242**	8	A
5	V	576.339	**604.334**	**597.310**	7	V
6	I	689.423	**717.418**	**710.394**	6	I
7	V	788.492	816.487	**809.463**	5	V
8	A	859.529	887.524	**922.547**	4	L
9	D	974.556	1002.551	1051.589	3	E
10	H	1111.615	1139.610	1150.658	2	V
11	R	1267.716	1295.711	**1313.721**	1	Y

BioTools Version 3.2 Copyright © 1999-2008 Bruker Daltonik GmbH Page -2-

(**B**)

Figure S25. Annotated MS/MS spectrum of ion 1313.725 *m*/*z* detected in area #8. (**A**) MS/MS spectrum; (**B**) Peak list and amino acid sequence.

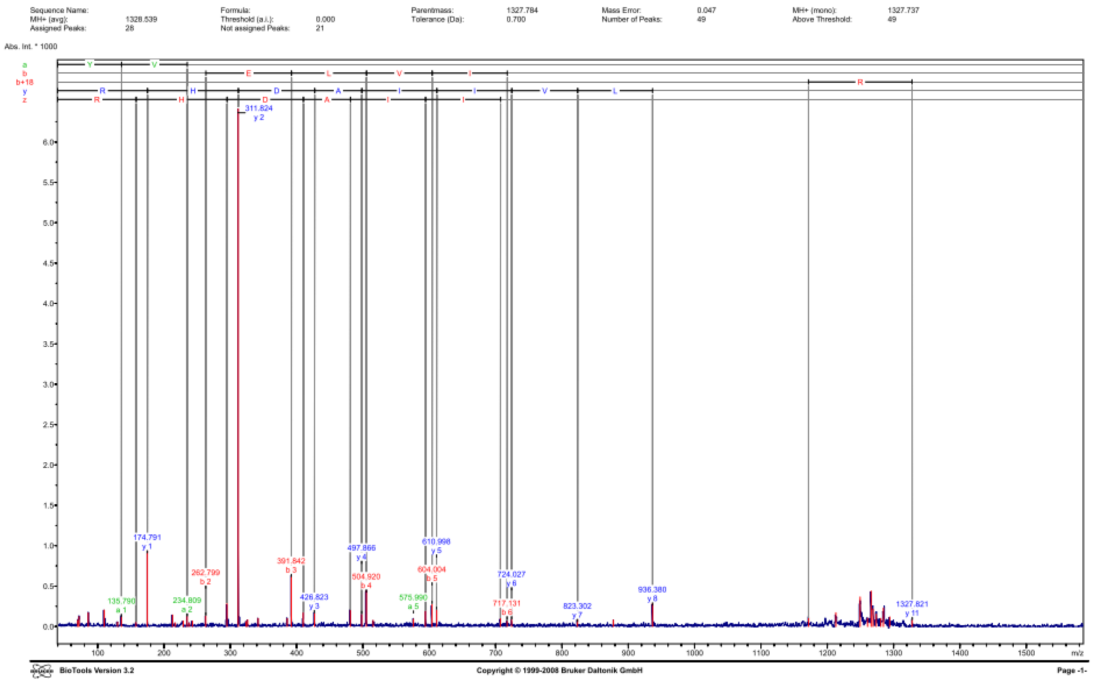

(**A**)

Display Parameter:

Parentmass: 1327.784 Mass Error: 0.047 MH+ (mono): 1327.737 MH+ (avg): 1328.539 Threshold (a.i.): 0.000
Tolerance (Da): 0.700 Number of Peaks: 49 Above Threshold: 49 Assigned Peaks: 28 Not assigned Peaks: 21

Peaklist:

Peak	Mass	Intensity	Peak	Mass	Intensity	Peak	Mass	Intensity	Peak	Mass	Intensity	Peak	Mass	Intensity	Peak	Mass	Intensity
1	69.825	76.833	2	71.804	123.079	3	85.838	169.349	4	109.800	204.148	5	129.654	55.923	6	135.790	125.809
7	157.760	58.172	8	174.791	939.928	9	212.821	142.789	10	216.943	46.626	11	228.809	69.116	12	234.809	132.888
13	238.202	43.973	14	242.812	68.378	15	262.799	137.755	16	294.806	291.509	17	311.824	6436.074	18	323.800	61.092
19	325.874	82.731	20	341.840	101.478	21	385.833	99.408	22	391.842	646.945	23	409.815	177.273	24	426.823	172.359
25	480.864	206.353	26	497.866	157.917	27	504.920	453.614	28	515.336	78.026	29	575.990	91.326	30	593.954	197.739
31	604.004	280.446	32	610.998	209.061	33	707.084	107.421	34	717.131	70.159	35	724.027	89.748	36	823.302	88.774
37	877.316	80.385	38	936.380	293.190	39	1171.657	110.569	40	1212.821	171.127	41	1249.456	366.942	42	1260.474	103.423
43	1265.949	441.315	44	1268.949	218.909	45	1273.839	153.221	46	1280.465	101.600	47	1284.531	229.327	48	1293.891	118.548
49	1327.821	85.937															

Calculated Masses:
YVELVIIADHR

N-Term.	Ion	a	a-17	a-18	b	b-17	b-18	b+18	c	i	x	y	z	C-Term.	Ion
1	Y	**136.076**	119.049	118.065	164.071	147.044	146.060	182.081	181.097	**136.076**	201.098	**175.119**	**158.092**	11	R
2	V	**235.144**	218.118	**217.134**	**263.139**	246.112	245.128	281.150	280.166	**72.081**	338.157	**312.178**	**295.151**	10	H
3	E	364.187	347.160	346.176	**392.182**	375.155	374.171	**410.192**	**409.208**	102.055	453.184	**427.205**	**410.178**	9	D
4	L	477.271	460.244	459.260	**505.266**	488.239	487.255	523.276	522.292	**86.096**	524.221	**498.242**	**481.215**	8	A
5	V	**576.339**	559.313	558.329	**604.334**	587.308	586.324	622.345	621.361	**72.081**	637.305	**611.326**	**594.299**	7	I
6	I	689.423	672.397	671.413	**717.418**	700.392	699.408	735.429	734.445	**86.096**	750.389	**724.410**	**707.383**	6	I
7	I	802.507	785.481	784.497	830.502	813.476	812.492	848.513	847.529	**86.096**	849.458	**823.478**	806.452	5	V
8	A	873.544	856.518	855.534	901.539	884.513	883.529	919.550	918.566	44.049	962.542	**936.563**	919.536	4	L
9	D	988.571	971.545	970.561	1016.566	999.540	998.556	1034.577	1033.593	88.039	1091.584	1065.605	1048.579	3	E
10	H	1125.630	1108.604	1107.620	1153.625	1136.599	1135.615	**1171.636**	1170.652	**110.071**	1190.653	1164.674	1147.647	2	V
11	R	1281.731	1264.705	1263.721	1309.726	1292.700	1291.716	**1327.737**	1326.753	129.113	1353.716	**1327.737**	1310.710	1	Y

BioTools Version 3.2 Copyright © 1999-2008 Bruker Daltonik GmbH Page -2-

(**B**)

Figure S26. Annotated MS/MS spectrum of ion 1327.784 *m*/*z* detected in area #8. (**A**) MS/MS spectrum; (**B**) Peak list and amino acid sequence.

Sequence Name:		Formula:		Parentmass: 1327.857	Mass Error: 0.120
MH+ (avg): 1328.539		Threshold (a.i.): 0.000		Tolerance (Da): 0.700	Number of Peaks: 75
Assigned Peaks: 33		Not assigned Peaks: 42		MH+ (mono): 1327.737	Above Threshold: 75

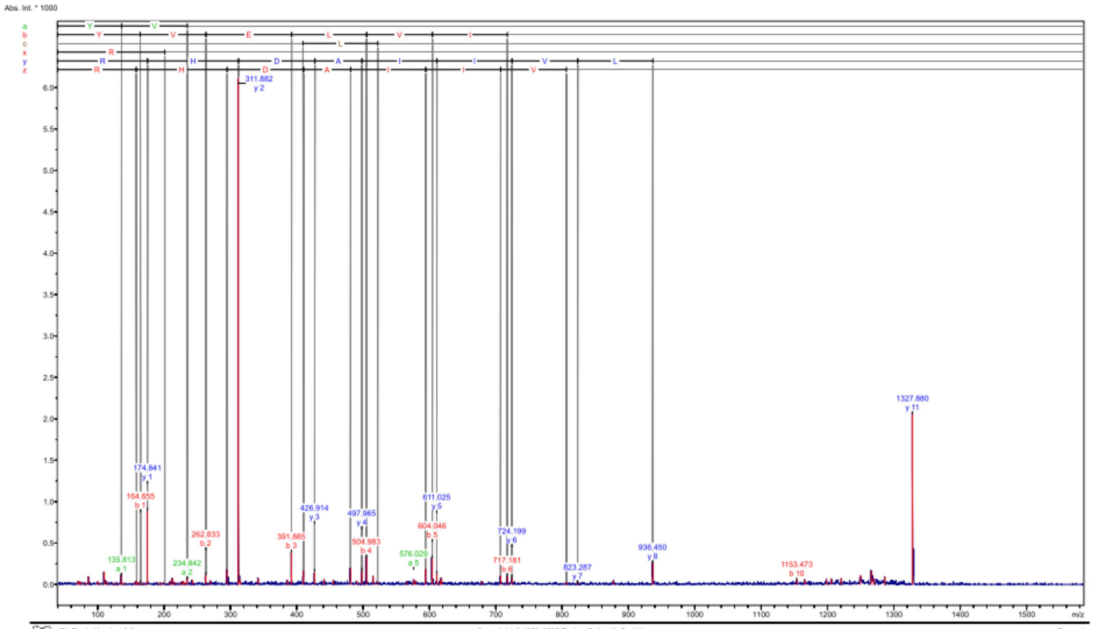

BioTools Version 3.2 Copyright © 1999-2008 Bruker Daltonik GmbH Page -1-

(**A**)

Display Parameter:

Parentmass:	1327.857	Mass Error:	0.120	MH+ (mono):	1327.737	MH+ (avg):	1328.539	Threshold (a.i.):	0.000		
Tolerance (Da):	0.700	Number of Peaks:	75	Above Threshold:	75	Assigned Peaks:	33	Not assigned Peaks:	42		

Peaklist:

Peak	Mass	Intensity	Peak	Mass	Intensity	Peak	Mass	Intensity	Peak	Mass	Intensity	Peak	Mass	Intensity	Peak	Mass	Intensity
1	69.824	36.126	2	71.812	35.036	3	74.778	26.680	4	80.937	22.039	5	85.864	91.285	6	99.855	30.451
7	102.150	20.611	8	109.835	151.723	9	113.912	34.319	10	122.119	15.478	11	126.913	17.427	12	133.266	15.162
13	135.813	114.599	14	140.470	15.805	15	155.787	22.543	16	158.781	46.523	17	162.150	20.322	18	164.655	13.631
19	169.604	16.178	20	174.841	890.131	21	191.288	16.056	22	195.380	21.092	23	201.172	16.991	24	209.162	33.920
25	212.858	74.767	26	216.852	24.917	27	224.837	23.940	28	228.802	35.660	29	230.773	24.608	30	234.842	71.237
31	242.881	51.102	32	262.833	114.620	33	269.850	43.647	34	294.855	187.076	35	311.882	6124.015	36	314.885	49.543
37	341.905	74.537	38	385.931	49.939	39	391.885	391.627	40	409.886	175.302	41	426.914	153.477	42	440.931	55.499
43	454.972	48.432	44	480.894	202.673	45	490.951	38.063	46	497.965	164.061	47	504.983	331.153	48	515.309	98.765
49	521.956	40.375	50	565.998	37.123	51	576.029	56.536	52	579.179	42.649	53	594.002	194.909	54	604.046	318.309
55	611.025	129.378	56	615.836	36.581	57	618.088	76.234	58	678.949	36.879	59	707.165	116.726	60	717.181	105.625
61	724.199	77.217	62	806.246	35.811	63	823.287	48.087	64	877.284	41.857	65	936.450	262.693	66	1153.473	56.289
67	1165.827	57.149	68	1198.543	59.509	69	1205.664	64.535	70	1220.799	71.477	71	1249.505	109.300	72	1265.898	162.894
73	1269.462	69.378	74	1285.973	89.158	75	1327.880	2061.121									

Calculated Masses:
YVELVIIADHR

N-Term.	Ion	a	a-17	a-18	b	b-17	b-18	b+18	c	i	x	y	z	C-Term.	Ion
1	Y	**136.076**	119.049	118.065	**164.071**	147.044	146.060	182.081	181.097	**136.076**	**201.098**	**175.119**	**158.092**	11	R
2	V	**235.144**	218.118	**217.134**	**263.139**	246.112	245.128	281.150	280.166	**72.081**	338.157	**312.178**	**295.151**	10	H
3	E	364.187	347.160	346.176	**392.182**	375.155	374.171	**410.192**	**409.208**	**102.055**	453.184	**427.205**	**410.178**	9	D
4	L	477.271	460.244	459.260	**505.266**	488.239	487.255	523.276	**522.292**	**86.096**	524.221	**498.242**	**481.215**	8	A
5	V	**576.339**	559.313	558.329	**604.334**	587.308	586.324	622.345	621.361	**72.081**	637.305	**611.326**	**594.299**	7	I
6	I	689.423	672.397	671.413	**717.418**	700.392	699.408	735.429	734.445	**86.096**	750.389	**724.410**	**707.383**	6	I
7	I	802.507	785.481	784.497	830.502	813.476	812.492	848.513	847.529	**86.096**	849.458	**823.478**	**806.452**	5	V
8	A	873.544	856.518	855.534	901.539	884.513	883.529	919.550	918.566	44.049	962.542	**936.563**	919.536	4	L
9	D	988.571	971.545	970.561	1016.566	999.540	998.556	1034.577	1033.593	88.039	1091.584	1065.605	1048.579	3	E
10	H	1125.630	1108.604	1107.620	**1153.625**	1136.599	1135.615	1171.636	1170.652	**110.071**	1190.653	1164.674	1147.647	2	V
11	R	1281.731	1264.705	1263.721	1309.726	1292.700	1291.716	**1327.737**	1326.753	129.113	1353.716	**1327.737**	1310.710	1	Y

BioTools Version 3.2 Copyright © 1999-2008 Bruker Daltonik GmbH Page -2-

(**B**)

Figure S27. Annotated MS/MS spectrum of ion 1327.857 *m/z* detected in area #8. (**A**) MS/MS spectrum; (**B**) Peak list and amino acid sequence.

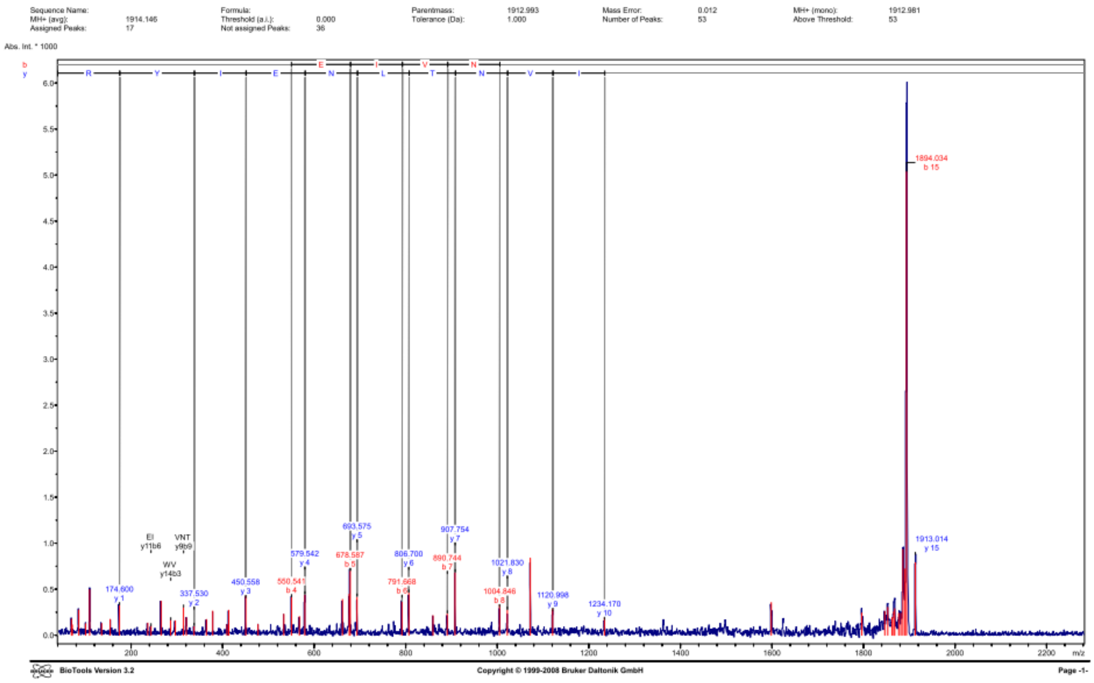

(**A**)

Display Parameter:

Parentmass:	1912.993	Mass Error:	0.012	MH+ (mono):	1912.981	MH+ (avg):	1914.146	Threshold (a.i.):	0.000
Tolerance (Da):	1.000	Number of Peaks:	53	Above Threshold:	53	Assigned Peaks:	17	Not assigned Peaks:	36

Peaklist:

Peak	Mass	Intensity	Peak	Mass	Intensity	Peak	Mass	Intensity	Peak	Mass	Intensity	Peak	Mass	Intensity	Peak	Mass	Intensity
1	69.741	177.178	2	71.729	113.845	3	85.728	275.503	4	100.650	126.243	5	109.625	498.080	6	135.601	129.201
7	154.680	173.896	8	174.600	333.416	9	236.543	133.174	10	243.513	99.481	11	265.534	370.102	12	286.534	164.760
13	296.488	157.790	14	314.506	301.815	15	320.529	191.708	16	337.530	103.200	17	364.530	172.033	18	378.547	262.932
19	409.541	114.851	20	413.521	271.790	21	450.558	433.887	22	477.568	118.161	23	533.527	229.241	24	550.541	421.127
25	567.557	204.689	26	579.542	446.314	27	661.583	389.449	28	676.584	446.923	29	678.587	705.217	30	693.575	421.644
31	791.668	369.304	32	806.700	465.626	33	859.668	213.051	34	890.744	238.835	35	907.754	686.660	36	1004.846	333.319
37	1021.830	279.344	38	1071.964	839.532	39	1120.998	294.084	40	1234.170	197.087	41	1598.419	356.688	42	1796.492	242.836
43	1846.626	258.868	44	1852.875	303.772	45	1862.258	193.338	46	1865.860	254.931	47	1868.161	289.940	48	1876.845	239.378
49	1881.656	239.737	50	1886.195	933.984	51	1890.921	720.813	52	1894.034	5039.537	53	1913.014	788.727			

Calculated Masses:
TWVYEIVNTLNEIYR

N-Term.	Ion	a	b	y	C-Term.	Ion
1	T	74.060	102.055	**175.119**	15	R
2	W	260.139	288.134	**338.182**	14	Y
3	V	359.208	387.203	**451.266**	13	I
4	Y	522.271	**550.266**	**580.309**	12	E
5	E	651.314	**679.309**	**694.352**	11	N
6	I	764.398	**792.393**	**807.436**	10	L
7	V	863.466	**891.461**	**908.484**	9	T
8	N	977.509	**1005.504**	**1022.527**	8	N
9	T	1078.557	1106.552	**1121.595**	7	V
10	L	1191.641	1219.636	**1234.679**	6	I
11	N	1305.684	1333.679	1363.722	5	E
12	E	1434.726	1462.721	1526.785	4	Y
13	I	1547.810	1575.805	1625.853	3	V
14	Y	1710.874	1738.869	1811.933	2	W
15	R	1866.975	**1894.970**	**1912.980**	1	T

BioTools Version 3.2 Copyright © 1999-2008 Bruker Daltonik GmbH Page -2-

(**B**)

Figure S28. Annotated MS/MS spectrum of ion 1912.993 *m*/*z* detected in area #8. (**A**) MS/MS spectrum; (**B**) Peak list and amino acid sequence.

Sequence Name: | Formula: | Parentmass: 2169.126 | Mass Error: -0.005 | MH+ (mono): 2169.130
MH+ (avg): 2170.406 | Threshold (a.i.): 0.000 | Tolerance (Da): 0.700 | Number of Peaks: 76 | Above Threshold: 76
Assigned Peaks: 54 | Not assigned Peaks: 22

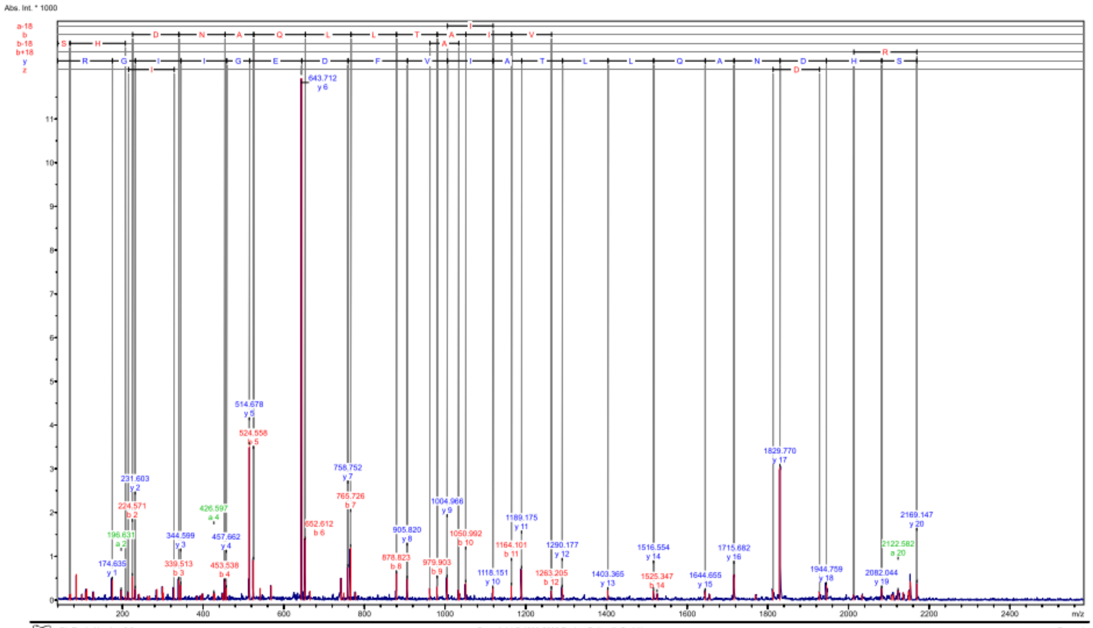

BioTools Version 3.2 Copyright © 1999-2008 Bruker Daltonik GmbH Page -1-

(**A**)

Display Parameter:

Parentmass: 2169.126 | Mass Error: -0.005 | MH+ (mono): 2169.130 | MH+ (avg): 2170.406 | Threshold (a.i.): 0.000
Tolerance (Da): 0.700 | Number of Peaks: 76 | Above Threshold: 76 | Assigned Peaks: 54 | Not assigned Peaks: 22

Peaklist:

Peak	Mass	Intensity	Peak	Mass	Intensity	Peak	Mass	Intensity	Peak	Mass	Intensity	Peak	Mass	Intensity	Peak	Mass	Intensity
1	69.771	142.105	2	71.720	153.930	3	85.743	585.258	4	99.703	120.702	5	109.702	188.465	6	111.668	250.719
7	128.656	177.505	8	174.635	477.441	9	196.631	221.808	10	207.565	92.591	11	214.604	185.371	12	224.571	548.869
13	231.603	258.974	14	241.592	132.355	15	262.533	75.385	16	267.590	92.013	17	285.590	247.299	18	299.559	296.857
19	301.535	160.029	20	327.607	288.953	21	339.513	473.667	22	344.599	351.990	23	384.627	91.153	24	394.574	100.175
25	398.622	143.261	26	426.597	155.640	27	428.526	135.896	28	448.503	149.736	29	453.538	488.389	30	457.662	275.480
31	514.678	3548.671	32	524.558	918.785	33	541.587	267.032	34	568.664	328.004	35	643.712	12016.913	36	652.612	1442.838
37	664.820	205.510	38	741.727	489.514	39	748.736	163.313	40	758.752	751.788	41	765.726	1206.454	42	778.130	172.210
43	878.823	622.602	44	905.820	484.658	45	961.909	274.410	46	979.903	317.296	47	1004.966	512.137	48	1032.923	241.664
49	1034.896	154.202	50	1050.992	397.776	51	1118.151	322.726	52	1164.101	331.483	53	1189.175	715.873	54	1263.205	214.937
55	1290.177	283.958	56	1403.365	242.059	57	1516.554	226.897	58	1525.347	142.065	59	1644.655	216.098	60	1655.570	124.710
61	1715.682	600.309	62	1769.904	116.246	63	1812.835	250.965	64	1829.770	3058.274	65	1927.906	192.242	66	1944.759	358.266
67	2012.678	128.327	68	2033.153	112.099	69	2082.044	276.389	70	2107.228	111.343	71	2110.910	114.925	72	2122.582	211.166
73	2135.656	165.540	74	2148.875	198.522	75	2152.722	423.516	76	2169.147	394.962						

Calculated Masses:
SHDNAQLLTAIVFDEGIIGR

N-Term.	Ion	a	a-17	a-18	b	b-17	b-18	b+18	c	i	x	y	z	C-Term.	Ion
1	S	60.044	43.018	42.034	88.039	71.013	**70.029**	106.050	105.066	60.044	201.098	**175.119**	158.092	20	R
2	H	**197.103**	180.077	179.093	**225.098**	**208.072**	**207.088**	243.109	**242.125**	**110.071**	258.120	**232.140**	**215.114**	19	G
3	D	312.130	295.104	294.120	**340.125**	323.099	322.115	358.136	357.152	88.039	371.204	**345.224**	**328.198**	18	I
4	N	**426.173**	409.147	408.163	**454.168**	437.142	436.158	472.179	471.195	87.055	484.288	**458.309**	441.282	17	I
5	A	497.210	480.184	479.200	**525.205**	508.179	507.195	543.216	**542.232**	44.049	**541.309**	**515.330**	498.303	16	G
6	Q	625.269	608.242	607.258	**653.264**	636.237	635.253	671.274	670.290	101.071	670.352	**644.373**	627.346	15	E
7	L	738.353	721.326	720.342	**766.348**	**749.321**	**748.337**	784.358	783.374	**86.096**	785.379	**759.400**	**742.373**	14	D
8	L	851.437	834.410	833.426	**879.432**	862.405	861.421	897.442	896.458	**86.096**	932.447	**906.468**	889.441	13	F
9	T	952.485	935.458	934.474	**980.480**	963.453	**962.469**	998.490	997.506	74.060	1031.516	**1005.536**	988.510	12	V
10	A	1023.522	1006.495	**1005.511**	**1051.517**	**1034.490**	**1033.506**	1069.527	1068.543	44.049	1144.600	**1118.620**	1101.594	11	I
11	I	1136.606	1119.579	**1118.595**	**1164.601**	1147.574	1146.590	1182.611	1181.627	**86.096**	1215.637	**1189.658**	1172.631	10	A
12	V	1235.674	1218.648	1217.664	**1263.669**	1246.643	1245.659	1281.680	1280.696	**72.081**	1316.684	**1290.705**	1273.679	9	T
13	F	1382.743	1365.716	1364.732	1410.738	1393.711	1392.727	1428.748	1427.764	120.081	1429.789	**1403.789**	1386.763	8	L
14	D	1497.770	1480.743	1479.759	**1525.765**	1508.738	1507.754	1543.775	1542.791	88.039	1542.853	**1516.873**	1499.847	7	L
15	E	1626.812	1609.786	1608.802	1654.807	1637.781	1636.797	1672.818	1671.834	102.055	1670.911	**1644.932**	1627.905	6	Q
16	G	1683.834	1666.807	1665.823	1711.829	1694.802	1693.818	1729.839	1728.855	30.034	1741.948	**1715.969**	1698.942	5	A
17	I	1796.918	1779.891	1778.907	1824.913	1807.886	1806.902	1842.923	1841.939	**86.096**	1855.991	**1830.012**	**1812.985**	4	N
18	I	1910.002	1892.975	1891.991	1937.997	1920.970	1919.986	1956.007	1955.023	**86.096**	1971.018	**1945.039**	**1928.012**	3	D
19	G	1967.023	1949.997	1949.013	1995.018	1977.992	1977.008	**2013.029**	**2012.045**	30.034	2108.077	**2082.098**	2065.071	2	H
20	R	**2123.124**	2106.098	2105.114	2151.119	2134.093	2133.109	**2169.130**	2168.146	**129.113**	2195.109	**2169.130**	**2152.103**	1	S

BioTools Version 3.2 Copyright © 1999-2008 Bruker Daltonik GmbH Page -2-

(**B**)

Figure S29. Annotated MS/MS spectrum of ion 2169.126 *m/z* detected in area #8. (**A**) MS/MS spectrum; (**B**) Peak list and amino acid sequence.

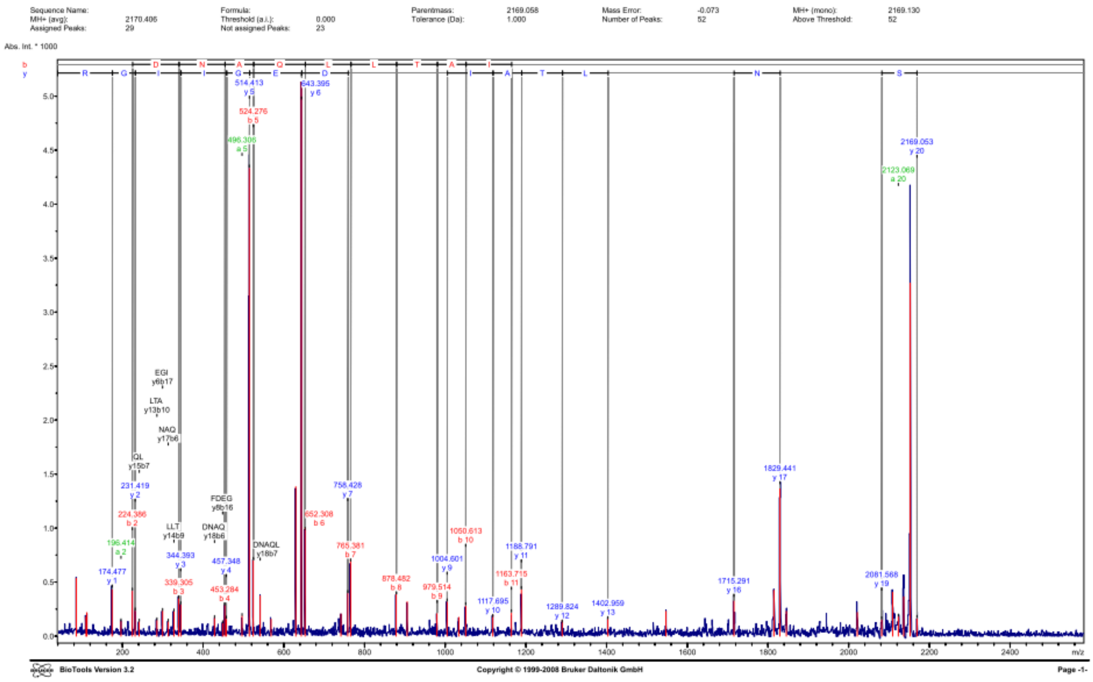

(A)

Display Parameter:

Parentmass: 2169.058 Mass Error: -0.073 MH+ (mono): 2169.130 MH+ (avg): 2170.406 Threshold (a.i.): 0.000
Tolerance (Da): 1.000 Number of Peaks: 52 Above Threshold: 52 Assigned Peaks: 29 Not assigned Peaks: 23

Peaklist:

Peak	Mass	Intensity	Peak	Mass	Intensity	Peak	Mass	Intensity	Peak	Mass	Intensity	Peak	Mass	Intensity	Peak	Mass	Intensity
1	85.629	536.295	2	109.590	177.283	3	111.550	218.652	4	174.477	435.842	5	196.414	136.991	6	224.386	422.501
7	231.419	237.341	8	241.407	134.628	9	285.392	145.981	10	299.364	232.804	11	313.326	134.613	12	327.341	228.485
13	339.305	374.150	14	344.393	294.395	15	428.289	167.394	16	448.268	118.330	17	453.284	289.469	18	457.348	162.064
19	496.306	187.136	20	514.413	4339.185	21	524.276	705.359	22	541.305	373.418	23	568.372	157.126	24	629.454	1384.764
25	643.395	5207.791	26	652.308	1008.012	27	741.413	205.769	28	758.428	398.776	29	765.381	715.588	30	878.482	391.589
31	905.416	319.169	32	979.514	214.669	33	1004.601	321.049	34	1033.575	174.515	35	1050.613	286.708	36	1117.695	200.821
37	1163.715	220.815	38	1188.791	435.057	39	1289.824	150.968	40	1402.959	162.236	41	1547.121	230.023	42	1715.291	331.073
43	1814.706	425.548	44	1829.441	1373.295	45	1845.318	230.438	46	2021.227	219.077	47	2081.568	155.020	48	2109.125	406.572
49	2123.069	181.064	50	2136.051	364.406	51	2152.408	3269.660	52	2169.053	167.930						

Calculated Masses:
SHDNAQLLTAIVFDEGIIGR

N-Term.	Ion	a	b	y	C-Term.	Ion
1	S	60.044	88.039	**175.119**	20	R
2	H	**197.103**	**225.098**	**232.140**	19	G
3	D	312.130	**340.125**	**345.224**	18	I
4	N	426.173	**454.168**	**458.309**	17	I
5	A	**497.210**	**525.205**	**515.330**	16	G
6	Q	625.269	**653.264**	**644.373**	15	E
7	L	738.353	**766.348**	**759.400**	14	D
8	L	851.437	**879.432**	906.468	13	F
9	T	952.485	**980.480**	**1005.536**	12	V
10	A	1023.522	**1051.517**	**1118.620**	11	I
11	I	1136.606	**1164.601**	**1189.658**	10	A
12	V	1235.674	1263.669	**1290.705**	9	T
13	F	1382.743	1410.738	**1403.789**	8	L
14	D	1497.770	1525.765	1516.873	7	L
15	E	1626.812	1654.807	1644.932	6	Q
16	G	1683.834	1711.829	**1715.969**	5	A
17	I	1796.918	1824.913	**1830.012**	4	N
18	I	1910.002	1937.997	1945.039	3	D
19	G	1967.023	1995.018	**2082.098**	2	H
20	R	**2123.124**	2151.119	**2169.130**	1	S

BioTools Version 3.2 Copyright © 1999-2008 Bruker Daltonik GmbH Page -2-

(B)

Figure S30. Annotated MS/MS spectrum of ion 2169.058 *m*/*z* detected in area #8. (**A**) MS/MS spectrum; (**B**) Peak list and amino acid sequence.

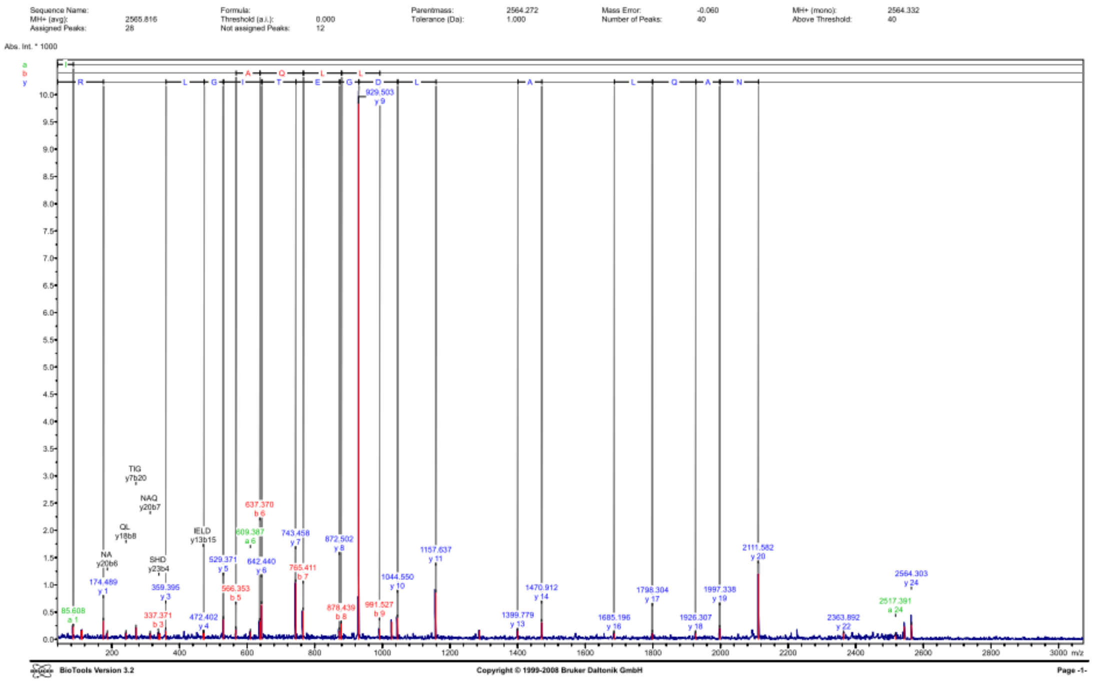

(A)

Display Parameter:

Parentmass: 2564.272 Mass Error: -0.060 MH+ (mono): 2564.332 MH+ (avg): 2565.816 Threshold (a.i.): 0.000
Tolerance (Da): 1.000 Number of Peaks: 40 Above Threshold: 40 Assigned Peaks: 28 Not assigned Peaks: 12

Peaklist:

Peak	Mass	Intensity	Peak	Mass	Intensity	Peak	Mass	Intensity	Peak	Mass	Intensity	Peak	Mass	Intensity	Peak	Mass	Intensity
1	85.608	241.180	2	109.537	153.085	3	111.581	167.172	4	174.489	342.472	5	186.499	113.171	6	241.420	116.684
7	271.392	214.041	8	313.354	105.631	9	337.371	117.747	10	339.323	96.626	11	342.376	99.284	12	359.395	166.030
13	470.327	123.599	14	472.402	124.990	15	529.371	369.874	16	566.353	183.758	17	609.387	140.847	18	637.370	337.978
19	642.440	646.804	20	743.458	1048.105	21	765.411	529.119	22	872.502	166.637	23	878.439	335.844	24	929.503	9842.004
25	991.527	200.821	26	1027.520	318.067	27	1044.550	398.861	28	1157.637	861.840	29	1286.627	158.467	30	1399.779	164.849
31	1470.912	319.162	32	1685.196	119.174	33	1798.304	118.834	34	1926.307	119.583	35	1997.338	208.574	36	2111.582	1212.912
37	2363.892	121.044	38	2517.391	76.370	39	2543.112	233.235	40	2564.303	266.488						

Calculated Masses:
ISHDNAQLLTAIELDGETIGLANR

N-Term.	Ion	a	b	y	C-Term.	Ion
1	I	**86.096**	114.091	**175.119**	24	R
2	S	173.128	201.123	289.162	23	N
3	H	310.187	**338.182**	**360.199**	22	A
4	D	425.214	453.209	**473.283**	21	L
5	N	539.257	**567.252**	**530.305**	20	G
6	A	**610.294**	**638.289**	**643.389**	19	I
7	Q	738.353	**766.348**	**744.436**	18	T
8	L	851.437	**879.432**	**873.479**	17	E
9	L	964.521	**992.516**	**930.500**	16	G
10	T	1065.569	1093.564	**1045.527**	15	D
11	A	1136.606	1164.601	**1158.611**	14	L
12	I	1249.690	1277.685	1287.654	13	E
13	E	1378.733	1406.727	**1400.738**	12	I
14	L	1491.817	1519.811	**1471.775**	11	A
15	D	1606.844	1634.838	1572.823	10	T
16	G	1663.865	1691.860	**1685.907**	9	L
17	E	1792.908	1820.902	**1798.991**	8	L
18	T	1893.955	1921.950	**1927.049**	7	Q
19	I	2007.039	2035.034	**1998.087**	6	A
20	G	2064.061	2092.056	**2112.130**	5	N
21	L	2177.145	2205.140	2227.156	4	D
22	A	2248.182	2276.177	**2364.215**	3	H
23	N	2362.225	2390.220	2451.247	2	S
24	R	**2518.326**	2546.321	**2564.331**	1	I

BioTools Version 3.2 Copyright © 1999-2008 Bruker Daltonik GmbH Page -2-

(B)

Figure S31. Annotated MS/MS spectrum of ion 2564.272 *m*/*z* detected in area #8. (**A**) MS/MS spectrum; (**B**) Peak list and amino acid sequence.

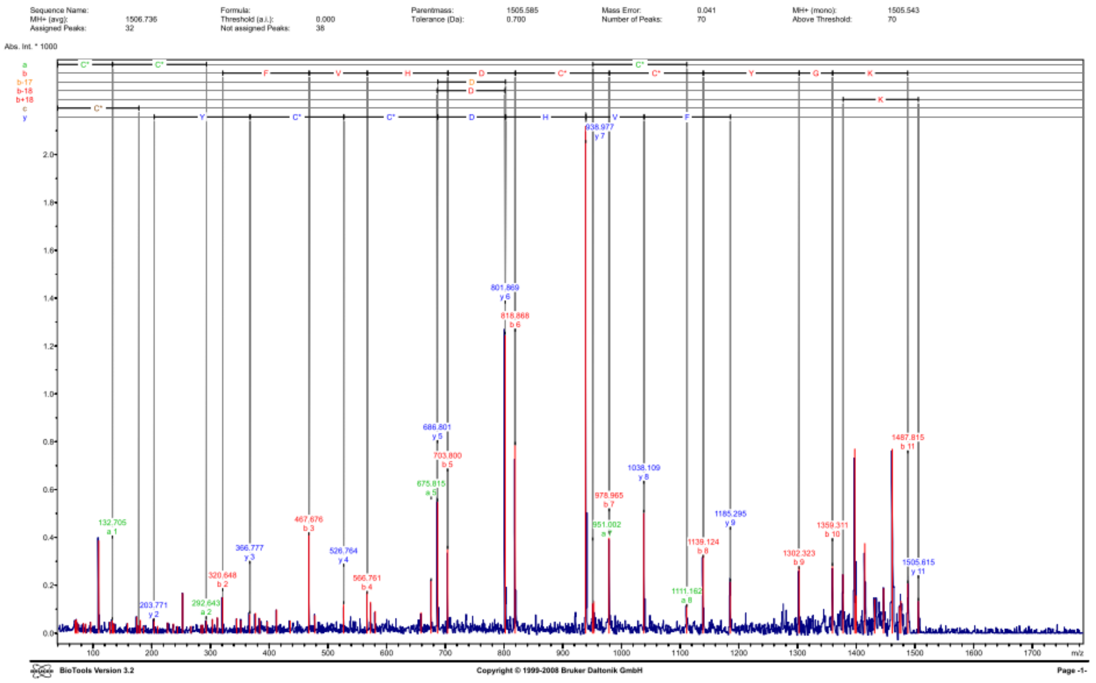

(**A**)

Display Parameter:

Parentmass:	1505.585	Mass Error:	0.041	MH+ (mono):	1505.543	MH+ (avg):	1506.736	Threshold (a.i.):	0.000
Tolerance (Da):	0.700	Number of Peaks:	70	Above Threshold:	70	Assigned Peaks:	32	Not assigned Peaks:	38

Peaklist:

Peak	Mass	Intensity	Peak	Mass	Intensity	Peak	Mass	Intensity	Peak	Mass	Intensity	Peak	Mass	Intensity	Peak	Mass	Intensity
1	69.832	52.705	2	71.791	56.001	3	74.229	40.412	4	83.507	41.066	5	87.668	38.484	6	96.102	45.825
7	109.748	386.250	8	128.766	45.444	9	132.705	54.049	10	134.316	32.460	11	135.726	39.869	12	158.721	40.225
13	174.755	67.341	14	177.690	33.253	15	180.724	53.513	16	186.354	28.187	17	203.771	60.965	18	210.116	28.028
19	227.042	42.787	20	236.742	38.649	21	243.733	28.171	22	252.668	166.518	23	266.527	28.889	24	284.234	34.171
25	286.834	33.028	26	292.643	51.959	27	303.637	57.806	28	311.755	65.258	29	320.648	150.973	30	344.660	59.745
31	351.763	52.915	32	366.777	80.492	33	376.684	82.983	34	383.761	59.243	35	396.856	48.004	36	412.663	97.127
37	435.722	51.067	38	467.676	419.794	39	477.947	74.280	40	526.764	121.364	41	566.761	175.043	42	572.718	129.682
43	580.676	90.806	44	658.792	85.840	45	675.815	217.179	46	686.801	550.028	47	703.800	352.397	48	801.869	1247.667
49	818.868	786.786	50	938.977	2173.722	51	951.002	125.650	52	952.964	132.843	53	978.965	414.688	54	1038.109	503.238
55	1111.162	120.123	56	1139.124	325.594	57	1185.295	214.668	58	1302.323	278.403	59	1359.311	281.111	60	1377.387	246.336
61	1397.414	769.311	62	1399.776	155.929	63	1414.589	374.492	64	1431.318	140.959	65	1446.612	186.446	66	1461.343	768.875
67	1473.947	109.969	68	1475.991	134.047	69	1487.815	207.889	70	1505.615	134.707						

Calculated Masses:

CCFVHDCCYGK 1: Carbamidomethyl (C) 2: Carbamidomethyl (C) 7: Carbamidomethyl (C) 8: Carbamidomethyl (C)

N-Term.	Ion	a	a-17	a-18	b	b-17	b-18	b+18	c	i	x	y	z	C-Term.	Ion
1	C*	**133.043**	116.016	115.032	161.038	144.011	143.027	179.048	**178.064**	**133.043**	173.092	147.113	130.086	11	K
2	C*	**293.074**	276.047	275.063	**321.069**	**304.042**	**303.058**	339.079	338.095	**133.043**	230.114	**204.134**	187.108	10	G
3	F	440.142	423.116	422.132	**468.137**	451.110	450.126	486.148	485.164	120.081	393.177	**367.198**	350.171	9	Y
4	V	539.210	522.184	521.200	**567.205**	550.179	549.195	585.216	584.232	**72.081**	553.208	**527.228**	510.202	8	C*
5	H	**676.269**	**659.243**	**658.259**	**704.264**	**687.238**	**686.254**	722.275	721.291	**110.071**	713.238	**687.259**	670.232	7	C*
6	D	791.296	774.270	773.286	**819.291**	**802.265**	**801.281**	837.302	836.318	**88.039**	828.265	**802.286**	785.259	6	D
7	C*	**951.327**	934.300	933.316	**979.322**	962.295	961.311	997.332	996.348	**133.043**	965.324	**939.345**	922.318	5	H
8	C*	**1111.358**	1094.331	1093.347	**1139.353**	1122.326	1121.342	1157.363	1156.379	**133.043**	1064.392	**1038.413**	1021.387	4	V
9	Y	1274.421	1257.394	1256.410	**1302.416**	1285.389	1284.405	1320.426	1319.442	**136.076**	1211.461	**1185.482**	1168.455	3	F
10	G	1331.442	1314.416	1313.432	**1359.437**	1342.411	1341.427	**1377.448**	1376.464	30.034	1371.491	1345.512	1328.486	2	C*
11	K	1459.537	1442.511	1441.527	**1487.532**	1470.506	1469.522	**1505.543**	1504.559	101.107	1531.522	**1505.543**	1488.516	1	C*

BioTools Version 3.2 Copyright © 1999-2008 Bruker Daltonik GmbH Page -2-

(**B**)

Figure S32. Annotated MS/MS spectrum of ion 1505.585 *m*/*z* detected in area #9. (**A**) MS/MS spectrum; (**B**) Peak list and amino acid sequence.

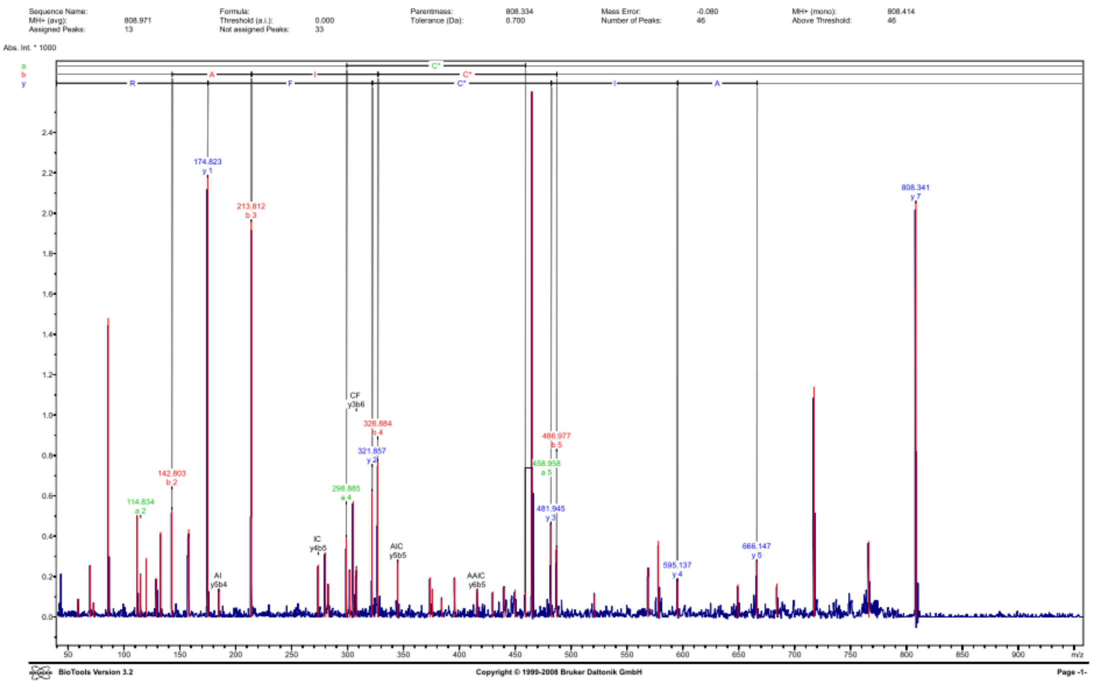

(**A**)

Display Parameter:

Parentmass: 808.334 Mass Error: -0.080 MH+ (mono): 808.414 MH+ (avg): 808.971 Threshold (a.i.): 0.000
Tolerance (Da): 0.700 Number of Peaks: 46 Above Threshold: 46 Assigned Peaks: 13 Not assigned Peaks: 33

Peaklist:

Peak	Mass	Intensity	Peak	Mass	Intensity	Peak	Mass	Intensity	Peak	Mass	Intensity	Peak	Mass	Intensity	Peak	Mass	Intensity
1	58.832	88.718	2	69.834	254.259	3	72.825	69.787	4	85.857	1480.027	5	111.822	515.279	6	114.834	201.201
7	119.821	290.681	8	128.834	191.112	9	132.772	419.814	10	142.803	530.372	11	157.810	432.298	12	174.823	2189.605
13	184.819	139.046	14	213.812	1968.248	15	273.823	257.585	16	279.785	319.689	17	282.858	161.255	18	298.885	393.147
19	302.008	228.045	20	304.850	571.364	21	307.784	238.280	22	321.857	621.000	23	326.884	767.338	24	344.845	284.300
25	373.827	194.542	26	375.858	136.082	27	384.209	90.716	28	395.951	190.348	29	415.917	138.302	30	429.940	123.256
31	439.913	150.606	32	449.871	133.921	33	458.958	119.911	34	464.921	2691.596	35	481.945	471.043	36	486.977	340.255
37	520.986	113.991	38	568.903	244.157	39	578.040	374.637	40	595.137	190.471	41	649.113	159.944	42	666.147	274.532
43	684.112	164.258	44	717.268	1139.085	45	766.314	373.820	46	808.341	2061.755						

Calculated Masses:

AAAICFR 5: Carbamidomethyl (C)

N-Term.	Ion	a	b	y	C-Term.	Ion
1	A	44.049	72.044	**175.119**	7	R
2	A	**115.087**	**143.082**	**322.187**	6	F
3	A	186.124	**214.119**	**482.218**	5	C*
4	I	**299.208**	**327.203**	**595.302**	4	I
5	C*	**459.238**	**487.233**	**666.339**	3	A
6	F	606.307	634.302	737.376	2	A
7	R	762.408	790.403	**808.413**	1	A

BioTools Version 3.2 Copyright © 1999-2008 Bruker Daltonik GmbH Page -2-

(**B**)

Figure S33. Annotated MS/MS spectrum of ion 808.334 *m/z* detected in area #9. (**A**) MS/MS spectrum; (**B**) Peak list and amino acid sequence.

Sequence Name:		Formula:		Parentmass:	980.433	Mass Error:	0.040	MH+ (mono):	980.393
MH+ (avg):	981.090	Threshold (a.i.):	0.000	Tolerance (Da):	0.700	Number of Peaks:	51	Above Threshold:	51
Assigned Peaks:	14	Not assigned Peaks:	37						

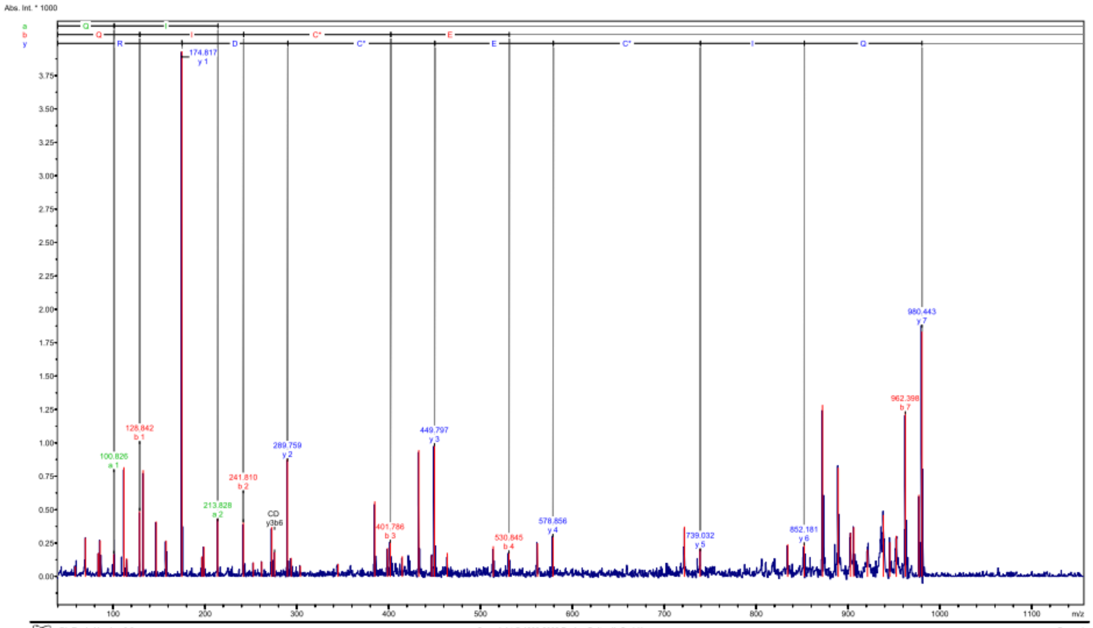

BioTools Version 3.2 Copyright © 1999-2008 Bruker Daltonik GmbH Page -1-

(**A**)

Display Parameter:

Parentmass:	980.433	Mass Error:	0.040	MH+ (mono):	980.393	MH+ (avg):	981.090	Threshold (a.i.):	0.000
Tolerance (Da):	0.700	Number of Peaks:	51	Above Threshold:	51	Assigned Peaks:	14	Not assigned Peaks:	37

Peaklist:

Peak	Mass	Intensity	Peak	Mass	Intensity	Peak	Mass	Intensity	Peak	Mass	Intensity	Peak	Mass	Intensity	Peak	Mass	Intensity
1	58.865	72.130	2	69.869	286.139	3	83.840	172.123	4	85.860	267.806	5	100.826	169.770	6	111.840	815.402
7	114.836	132.898	8	128.842	491.344	9	132.773	792.935	10	146.783	409.612	11	157.802	265.642	12	174.817	4012.122
13	196.808	147.356	14	198.754	219.608	15	213.828	433.000	16	241.810	406.601	17	252.734	102.759	18	261.756	110.233
19	272.780	374.965	20	275.730	180.475	21	289.759	881.941	22	293.814	136.527	23	303.777	76.634	24	344.821	91.532
25	384.796	559.858	26	398.845	210.272	27	401.786	272.303	28	414.787	148.498	29	432.794	943.904	30	446.817	156.359
31	449.797	995.196	32	463.828	173.345	33	513.839	224.074	34	530.845	192.611	35	561.864	246.186	36	578.856	299.652
37	721.954	370.431	38	739.032	208.015	39	834.212	238.017	40	852.181	252.748	41	872.179	1282.094	42	889.256	811.664
43	902.646	324.499	44	906.146	366.964	45	921.155	197.589	46	938.428	457.082	47	945.460	253.819	48	952.434	301.189
49	962.398	1234.465	50	977.171	608.977	51	980.443	1836.184									

Calculated Masses:

QICECDR 3: Carbamidomethyl (C) 5: Carbamidomethyl (C)

N-Term.	Ion	a	b	y	C-Term.	Ion
1	Q	**101.071**	**129.066**	**175.119**	7	R
2	I	**214.155**	**242.150**	**290.146**	6	D
3	C*	374.186	**402.181**	**450.177**	5	C*
4	E	503.228	**531.223**	**579.219**	4	E
5	C*	663.259	691.254	**739.250**	3	C*
6	D	778.286	806.281	**852.334**	2	I
7	R	934.387	**962.382**	**980.392**	1	Q

BioTools Version 3.2 Copyright © 1999-2008 Bruker Daltonik GmbH Page -2-

(**B**)

Figure S34. Annotated MS/MS spectrum of ion 980.433 *m*/*z* detected in area #9. (**A**) MS/MS spectrum. (**B**) Peak list and amino acid sequence.

Sequence Name: Acidic phospholipase A2 S1E6-b (Fragment) OS=Calloselasma rhodostoma PE=1 SV=1 PA2AB_CALRH
Mass Error: MH+ (mono): 1.008 MH+ (avg): 1.008 Formula: Threshold (a.i.): 0.000 Parentmass: Tolerance (Da): 0.500
Number of Peaks: 22 Above Threshold: Assigned Peaks: Not assigned Peaks:

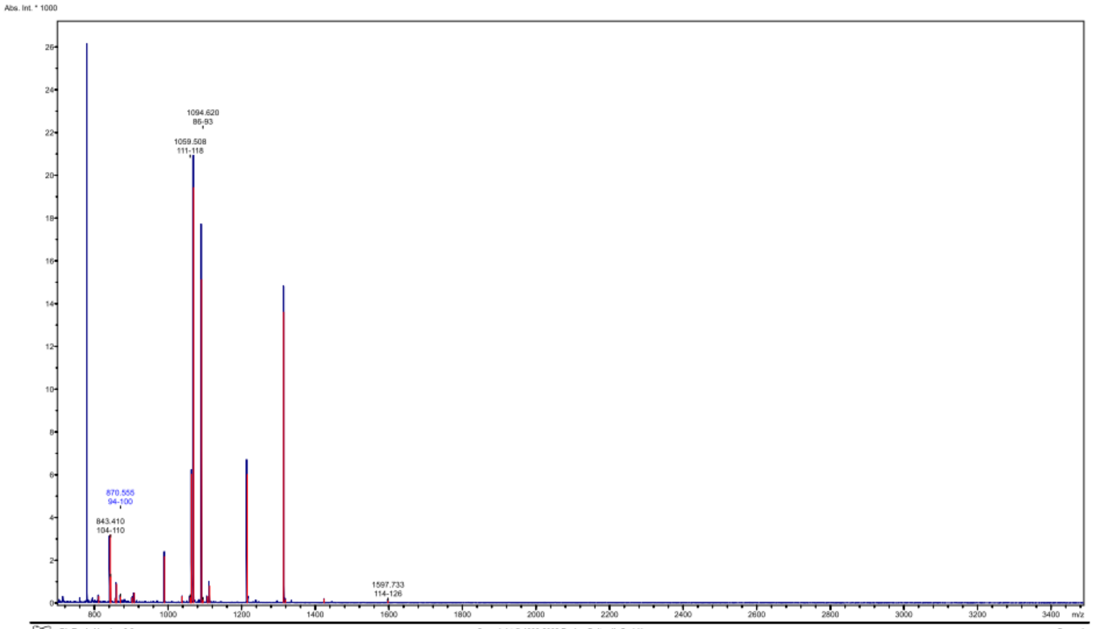

BioTools Version 3.2 Copyright © 1999-2008 Bruker Daltonik GmbH Page -1-

(**A**)

Sequence data:

Acidic phospholipase A2 S1E6-b (Fragment) OS=Calloselasma rhodostoma PE=1 SV=1 PA2AB_CALRH

Intensity Coverage: 5.7 % (4049 cnts) Sequence Coverage MS: 30.2%
Sequence Coverage MS/MS: 0.0% pI (isoelectric point): 4.8

```
        10         20         30         40         50         60         70         80         90        100        110
VEGSLVQFET LIMKLAKRSG FFWYSFYGCY CGWGGHGLPQ DPTDRCCFVH DCCYGKVTNC NPKTATYSYT EENDGIVCGG DDPCKKQVCE CDRVAAMCFR DNKDTYDSDK
       120        130
YWKLPPQKCQ EDPEPC
```

Display Parameter:

Sequence Name: Acidic phospholipase A2 S1E6-b (Fragment) OS=Calloselasma rhodostoma PE=1 SV=1 PA2AB_CALRH MH+ (mono): 1.008 MH+ (avg): 1.008
Threshold (a.i.): 0.000 Tolerance (Da): 0.500 Number of Peaks: 22

Peaklist:

Peak	Mass	Intensity	Peak	Mass	Intensity	Peak	Mass	Intensity	Peak	Mass	Intensity	Peak	Mass	Intensity	Peak	Mass	Intensity
1	811.426	304.939	2	842.500	3200.171	3	843.410	1224.188	4	856.514	197.957	5	859.472	873.147	6	870.555	301.610
7	902.386	235.762	8	907.483	461.651	9	989.493	2171.054	10	1037.544	300.281	11	1059.508	268.237	12	1063.570	6021.309
13	1068.566	19423.415	14	1090.530	15117.442	15	1094.620	141.792	16	1106.525	296.216	17	1112.518	791.959	18	1214.676	6012.595
19	1313.743	13596.590	20	1318.682	144.192	21	1423.831	180.596	22	1597.733	137.517						

BioTools Version 3.2 Copyright © 1999-2008 Bruker Daltonik GmbH Page -2-

(**B**)

Figure S35. Annotated MS spectrum detected in area #9. (**A**) MS spectrum; (**B**) Peak list and sequence data.

Sequence Name: Acidic phospholipase A2 A OS=Gloydius halys PE=1 SV=2 PA2A7_GLOHA
Mass Error: MH+ (mono): 1.008 MH+ (avg): 1.008 Formula: Threshold (a.i.): 0.000 Parentmass: Tolerance (Da): 0.500
Number of Peaks: 10 Above Threshold: Assigned Peaks: Not assigned Peaks:

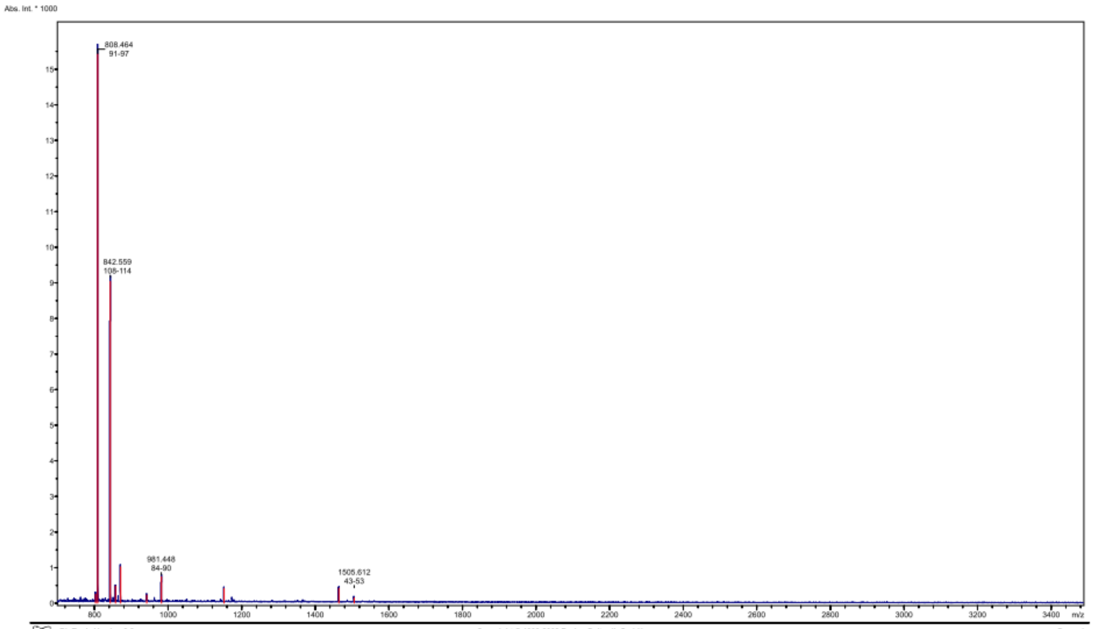

BioTools Version 3.2 Copyright © 1999-2008 Bruker Daltonik GmbH Page -1-

(**A**)

Sequence data:

Acidic phospholipase A2 A OS=Gloydius halys PE=1 SV=2 PA2A7_GLOHA

Intensity Coverage: 90.0 % (25361 cnts) Sequence Coverage MS: 25.8%
Sequence Coverage MS/MS: 0.0% pI (isoelectric point): 4.7

10	20	30	40	50	60	70	80	90
SLLQFETLIM	KVAKKSGMVW	YSNYGCYCGW	GGQGRPQDAT	DRCCFVHDCC	YGKVTGCDPK	MDVYSFSEEN	GDIVCGGDDP	CKKEICECDR
100	110	120	130					
AAAICFRDNL	NTYNDKKYWA	FGAKNCPQEE	SEPC					

Display Parameter:

Sequence Name: Acidic phospholipase A2 A OS=Gloydius halys PE=1 SV=2 PA2A7_GLOHA MH+ (mono): 1.008 MH+ (avg): 1.008
Threshold (a.i.): 0.000 Tolerance (Da): 0.500 Number of Peaks: 10

Peaklist:

Peak	Mass	Intensity	Peak	Mass	Intensity	Peak	Mass	Intensity	Peak	Mass	Intensity	Peak	Mass	Intensity	Peak	Mass	Intensity
1	802.432	251.082	2	808.464	15431.080	3	842.559	9038.824	4	856.578	483.287	5	870.597	1020.805	6	941.530	228.644
7	981.448	744.093	8	1151.667	411.634	9	1463.649	420.023	10	1505.612	146.838						

BioTools Version 3.2 Copyright © 1999-2008 Bruker Daltonik GmbH Page -2-

(**B**)

Figure S36. Annotated MS spectrum detected in area #9. (**A**) MS spectrum; (**B**) Peak list and sequence data.

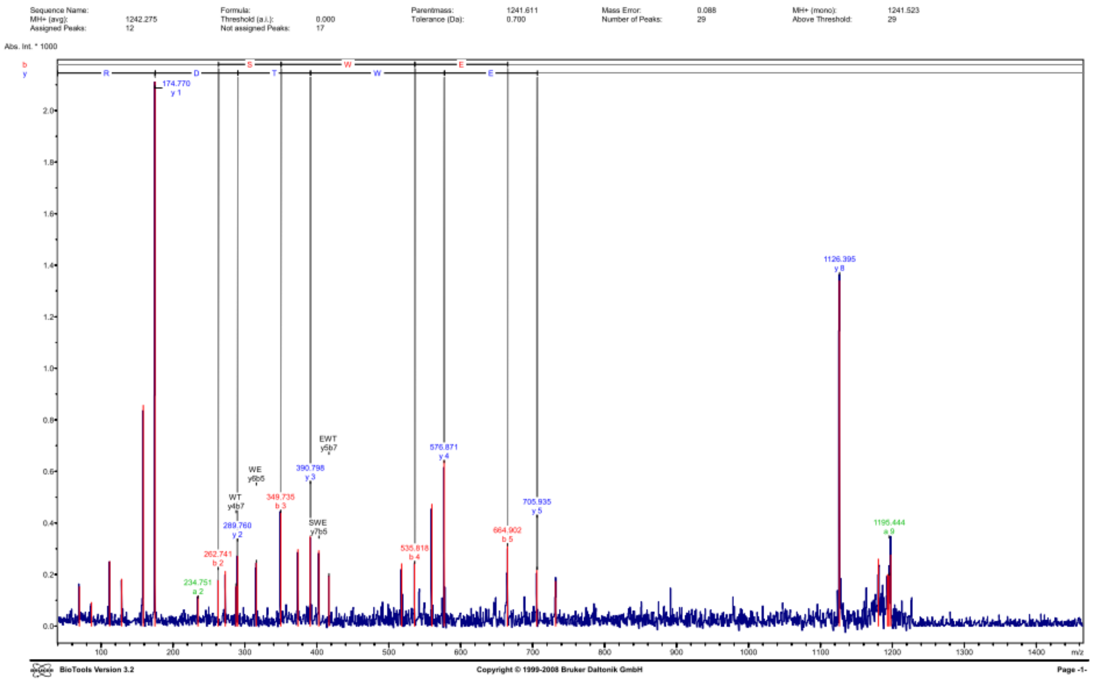

(**A**)

Display Parameter:

Parentmass:	1241.611	Mass Error:	0.088	MH+ (mono):	1241.523
Tolerance (Da):	0.700	Number of Peaks:	29	Above Threshold:	29
MH+ (avg):	1242.275	Threshold (a.i.):	0.000		
Assigned Peaks:	12	Not assigned Peaks:	17		

Peaklist:

Peak	Mass	Intensity	Peak	Mass	Intensity	Peak	Mass	Intensity	Peak	Mass	Intensity	Peak	Mass	Intensity	Peak	Mass	Intensity
1	69.824	153.143	2	86.804	92.239	3	111.799	250.811	4	128.777	182.475	5	158.759	856.401	6	174.770	2143.175
7	234.751	117.462	8	262.741	179.331	9	272.729	212.644	10	287.746	154.516	11	289.760	273.391	12	315.731	245.685
13	349.735	449.980	14	373.768	297.373	15	390.798	349.961	16	402.771	293.165	17	416.770	193.779	18	517.805	241.827
19	535.818	251.347	20	559.830	472.878	21	576.871	643.071	22	664.902	320.332	23	705.935	218.936	24	731.978	173.624
25	1126.395	1339.750	26	1180.631	260.315	27	1193.223	200.184	28	1195.444	195.656	29	1197.552	276.102			

Calculated Masses:
DFSWEWTDR

N-Term.	Ion	a	b	y	C-Term.	Ion
1	D	88.039	116.034	**175.119**	9	R
2	F	**235.108**	**263.103**	**290.146**	8	D
3	S	322.140	**350.135**	**391.194**	7	T
4	W	508.219	**536.214**	**577.273**	6	W
5	E	637.262	**665.257**	**706.315**	5	E
6	W	823.341	851.336	892.395	4	W
7	T	924.389	952.384	979.427	3	S
8	D	1039.416	1067.411	**1126.495**	2	F
9	R	**1195.517**	1223.512	1241.522	1	D

BioTools Version 3.2 Copyright © 1999-2008 Bruker Daltonik GmbH Page -2-

(**B**)

Figure S37. Annotated MS/MS spectrum of ion 1241.611 *m*/*z* detected in area #10. (**A**) MS/MS spectrum; (**B**) Peak list and amino acid sequence.

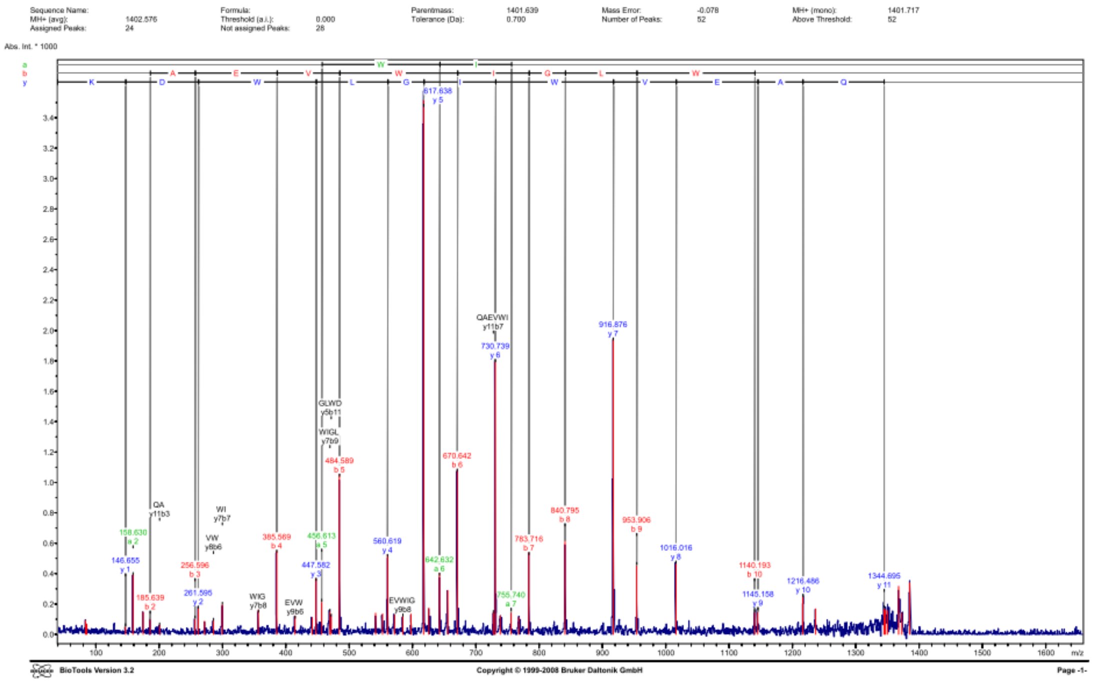

(**A**)

Display Parameter:

Parentmass: 1401.639 Mass Error: -0.078 MH+ (mono): 1401.717 MH+ (avg): 1402.576 Threshold (a.i.): 0.000
Tolerance (Da): 0.700 Number of Peaks: 52 Above Threshold: 52 Assigned Peaks: 24 Not assigned Peaks: 28

Peaklist:

Peak	Mass	Intensity	Peak	Mass	Intensity	Peak	Mass	Intensity	Peak	Mass	Intensity	Peak	Mass	Intensity	Peak	Mass	Intensity
1	83.682	91.326	2	85.701	66.663	3	146.655	53.339	4	158.630	389.803	5	174.627	146.306	6	185.639	98.998
7	200.620	58.643	8	256.596	104.305	9	261.595	186.312	10	271.597	86.180	11	285.604	89.040	12	299.601	194.804
13	356.604	161.897	14	385.569	554.032	15	414.582	120.989	16	441.591	109.058	17	447.582	370.058	18	456.613	215.188
19	469.644	149.267	20	471.600	116.816	21	484.589	1055.250	22	541.653	139.655	23	552.601	134.550	24	560.619	526.404
25	571.537	128.870	26	584.649	121.611	27	597.637	140.760	28	617.638	3517.521	29	625.633	173.209	30	642.632	404.123
31	655.633	288.836	32	670.642	1088.592	33	727.664	141.038	34	730.739	1796.613	35	738.614	122.413	36	755.740	145.288
37	767.805	111.014	38	783.716	540.081	39	840.795	618.505	40	916.876	1952.034	41	953.906	456.973	42	1016.016	469.291
43	1140.193	146.352	44	1145.158	150.377	45	1216.486	250.524	46	1236.671	168.292	47	1344.695	172.978	48	1346.716	161.838
49	1349.847	156.446	50	1367.558	316.178	51	1372.753	140.252	52	1385.052	339.872						

Calculated Masses:
GQAEVWIGLWDK

N-Term.	Ion	a	b	y	C-Term.	Ion
1	G	30.034	58.029	**147.113**	12	K
2	Q	**158.092**	**186.087**	**262.140**	11	D
3	A	229.130	**257.124**	**448.219**	10	W
4	E	358.172	**386.167**	**561.303**	9	L
5	V	**457.241**	**485.235**	**618.325**	8	G
6	W	**643.320**	**671.315**	**731.409**	7	I
7	I	**756.404**	**784.399**	**917.488**	6	W
8	G	813.425	**841.420**	**1016.556**	5	V
9	L	926.509	**954.504**	**1145.599**	4	E
10	W	1112.589	**1140.584**	**1216.636**	3	A
11	D	1227.616	1255.611	**1344.695**	2	Q
12	K	1355.711	1383.706	1401.716	1	G

BioTools Version 3.2 Copyright © 1999-2008 Bruker Daltonik GmbH Page -2-

(**B**)

Figure S38. Annotated MS/MS spectrum of ion 1401.639 *m/z* detected in area #10. (**A**) MS/MS spectrum; (**B**) Peak list and amino acid sequence.

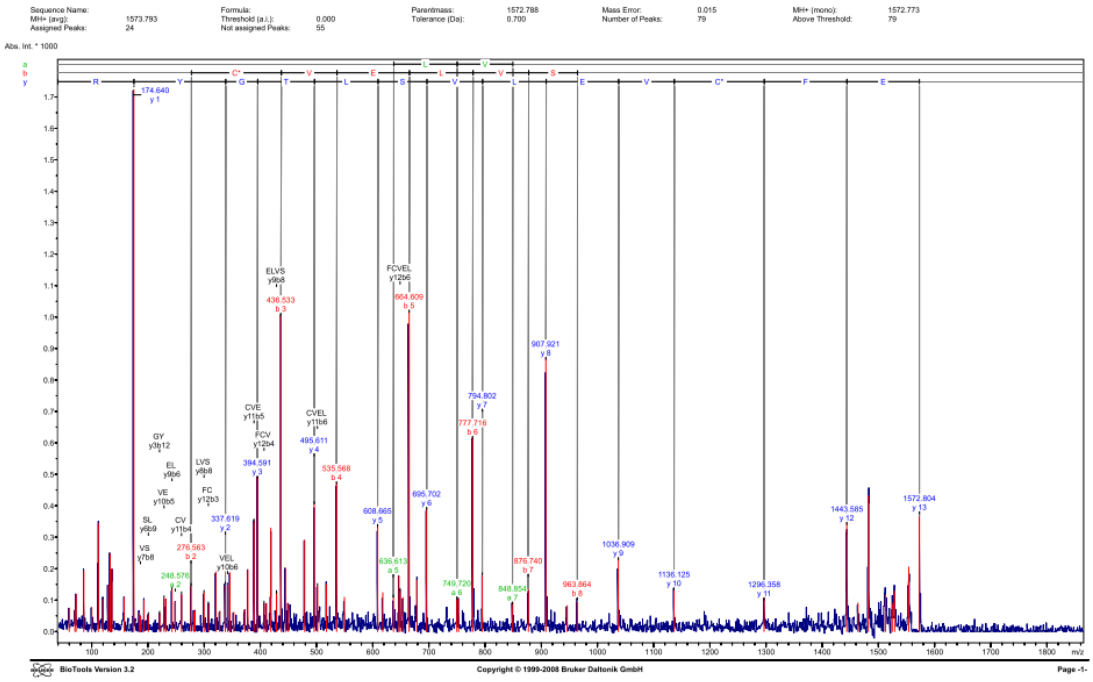

(A)

Display Parameter:

Parentmass: 1572.788 Mass Error: 0.015 MH+ (mono): 1572.773 MH+ (avg): 1573.793 Threshold (a.i.): 0.000
Tolerance (Da): 0.700 Number of Peaks: 79 Above Threshold: 79 Assigned Peaks: 24 Not assigned Peaks: 55

Peaklist:

Peak	Mass	Intensity	Peak	Mass	Intensity	Peak	Mass	Intensity	Peak	Mass	Intensity	Peak	Mass	Intensity	Peak	Mass	Intensity
1	59.734	74.275	2	69.750	69.619	3	71.757	118.230	4	85.732	194.403	5	99.723	76.544	6	111.683	344.015
7	119.664	103.061	8	128.714	147.112	9	132.598	246.230	10	135.640	198.694	11	157.647	108.516	12	174.640	1731.976
13	184.606	60.897	14	186.596	48.927	15	192.624	99.504	16	200.602	52.489	17	220.554	56.499	18	228.582	105.910
19	231.616	103.539	20	242.586	132.336	21	248.576	95.586	22	259.563	118.327	23	276.563	145.163	24	281.685	61.808
25	283.615	66.641	26	299.620	123.020	27	307.534	86.333	28	320.574	190.741	29	327.533	64.514	30	337.619	149.560
31	341.617	151.364	32	345.560	186.287	33	352.562	61.511	34	372.493	70.221	35	377.575	196.162	36	388.540	350.002
37	394.591	494.637	38	406.588	88.398	39	410.604	89.738	40	418.529	328.957	41	428.666	120.691	42	436.533	1005.248
43	444.586	201.328	44	449.557	87.174	45	478.587	290.227	46	495.611	404.697	47	501.581	144.540	48	517.591	150.350
49	535.568	476.709	50	549.570	108.660	51	608.665	340.968	52	617.640	123.430	53	636.613	108.105	54	646.600	176.528
55	648.655	129.360	56	653.647	107.555	57	664.609	1022.501	58	678.650	162.557	59	695.702	395.028	60	749.720	103.845
61	752.769	104.932	62	777.716	621.257	63	794.802	178.822	64	848.854	94.318	65	876.740	135.762	66	907.921	872.179
67	945.834	81.857	68	963.864	94.808	69	1036.909	235.413	70	1136.125	139.639	71	1296.358	107.709	72	1443.585	347.053
73	1463.437	90.950	74	1481.656	431.921	75	1512.261	117.407	76	1524.334	89.572	77	1528.841	130.674	78	1553.787	205.431
79	1572.804	367.736															

Calculated Masses:

EFCVELVSLTGYR 3: Carbamidomethyl (C)

N-Term.	Ion	a	b	y	C-Term.	Ion
1	E	102.055	130.050	**175.119**	13	R
2	F	**249.123**	**277.118**	**338.182**	12	Y
3	C*	409.154	**437.149**	**395.204**	11	G
4	V	508.222	**536.217**	**496.251**	10	T
5	E	**637.265**	**665.260**	**609.335**	9	L
6	L	**750.349**	**778.344**	**696.368**	8	S
7	V	**849.418**	**877.412**	**795.436**	7	V
8	S	936.450	**964.444**	**908.520**	6	L
9	L	1049.534	1077.529	**1037.563**	5	E
10	T	1150.581	1178.576	**1136.631**	4	V
11	G	1207.603	1235.598	**1296.662**	3	C*
12	Y	1370.666	1398.661	**1443.730**	2	F
13	R	1526.767	1554.762	**1572.773**	1	E

BioTools Version 3.2 Copyright © 1999-2008 Bruker Daltonik GmbH Page -2-

(B)

Figure S39. Annotated MS/MS spectrum of ion 1572.788 *m/z* detected in area #10. (**A**) MS/MS spectrum; (**B**) Peak list and amino acid sequence.

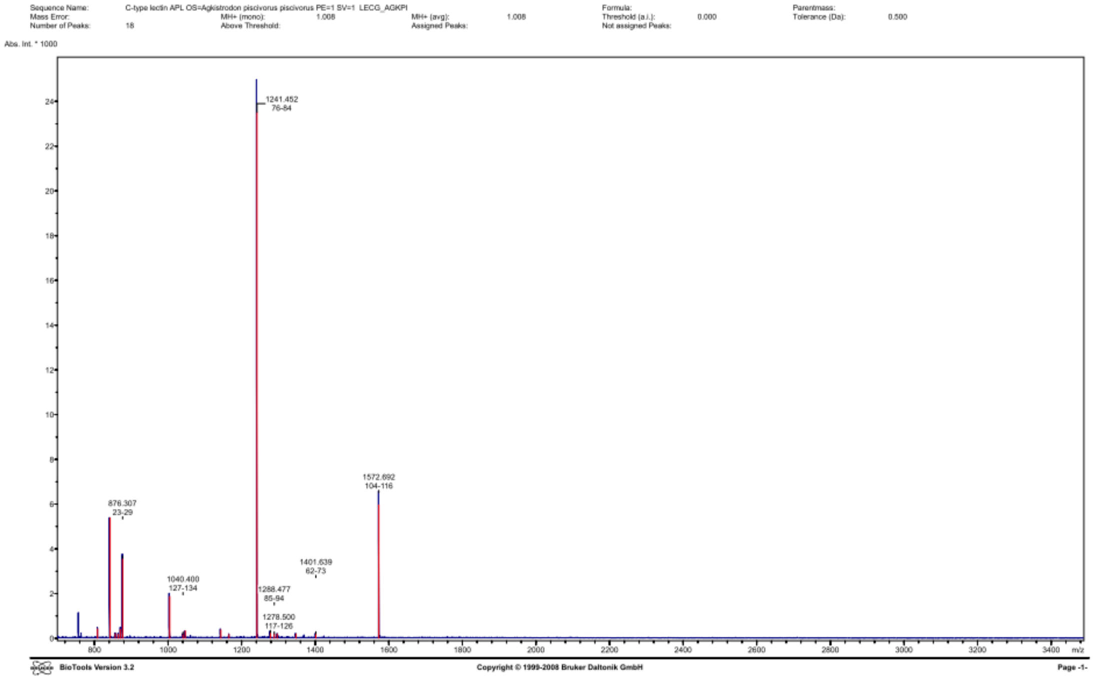

(**A**)

Sequence data:

C-type lectin APL OS=Agkistrodon piscivorus piscivorus PE=1 SV=1 LECG_AGKPI

Intensity Coverage: 77.6 % (33856 cnts)
Sequence Coverage MS/MS: 0.0%
Sequence Coverage MS: 51.1%
pI (isoelectric point): 5.0

10	20	30	40	50	60	70	80	90	100	110
NNCPHDWLPM	NGLCYKIFDE	LKAWEDAERF	CRKYKPGCHL	ASFHTYGESL	EIAEYISDYH	KGQAEVWIGL	WDKKKDFSWE	WTDRSCTDYL	TWDKNQPDVY	QNKEFCVELV
**120**	**130**	**140**								
SLTGYRLWND	QVCESKNAFL	CQCKF								

Display Parameter:

Sequence Name: C-type lectin APL OS=Agkistrodon piscivorus piscivorus PE=1 SV=1 LECG_AGKPI
MH+ (mono): 1.008
MH+ (avg): 1.008
Threshold (a.i.): 0.000
Tolerance (Da): 0.500
Number of Peaks: 18

Peaklist:

Peak	Mass	Intensity	Peak	Mass	Intensity	Peak	Mass	Intensity	Peak	Mass	Intensity	Peak	Mass	Intensity	Peak	Mass	Intensity
1	808.332	426.180	2	842.420	5383.154	3	856.446	210.551	4	864.401	174.554	5	870.464	404.758	6	876.307	3558.424
7	1004.409	1877.919	8	1040.400	175.227	9	1046.482	337.652	10	1143.452	380.465	11	1165.426	175.269	12	1241.452	23509.080
13	1278.500	270.331	14	1288.477	189.952	15	1296.619	197.672	16	1347.665	188.794	17	1401.639	190.351	18	1572.692	5963.056

BioTools Version 3.2 Copyright © 1999-2008 Bruker Daltonik GmbH Page -2-

(**B**)

Figure S40. Annotated MS spectrum detected in area #10. (**A**) MS spectrum; (**B**) Peak list and sequence data.

Sequence Name: C-type lectin PAL OS=Bitis arietans PE=1 SV=1 LECG_BITAR
MH+ (mono): 1.008 MH+ (avg): 1.008
Above Threshold: Assigned Peaks:
Formula:
Threshold (a.i.): 0.000
Not assigned Peaks:
Parentmass:
Tolerance (Da): 0.500
Mass Error:
Number of Peaks: 18

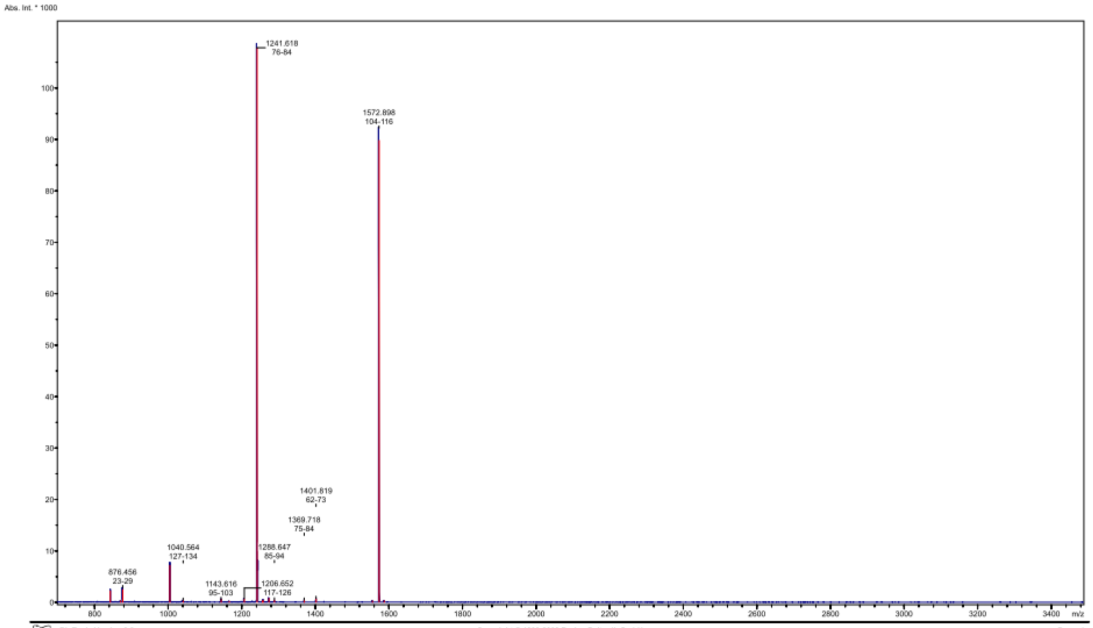

BioTools Version 3.2 Copyright © 1999-2008 Bruker Daltonik GmbH Page -1-

(**A**)

Sequence data:

C-type lectin PAL OS=Bitis arietans PE=1 SV=1 LECG_BITAR

Intensity Coverage: 94.5 % (203063 cnts)
Sequence Coverage MS/MS: 0.0%
Sequence Coverage MS: 58.5%
pI (isoelectric point): 5.2

10 20 30 40 50 60 70 80 90 100 110
NNCPPDWLPM NGLCYKIFDE LKAWEDAERF CRKYKPGCHL ASFHQYGESL EIAEYISDYH KGQAEVWIGL WDKKKDFSWE WTDRSCTDYL TWDKNQPDHY QNKEFCVELV
120 130 140
SLTGYRLWND QVCGSKNAFL CQCKF

Display Parameter:

Sequence Name: C-type lectin PAL OS=Bitis arietans PE=1 SV=1 LECG_BITAR
MH+ (mono): 1.008 MH+ (avg): 1.008 Threshold (a.i.): 0.000
Tolerance (Da): 0.500 Number of Peaks: 18

Peaklist:

Peak	Mass	Intensity	Peak	Mass	Intensity	Peak	Mass	Intensity	Peak	Mass	Intensity	Peak	Mass	Intensity	Peak	Mass	Intensity
1	842.580	2230.583	2	870.622	307.121	3	876.456	2472.224	4	1004.563	7183.649	5	1040.564	339.429	6	1143.616	590.006
7	1165.607	197.501	8	1206.652	655.763	9	1241.618	107757.303	10	1255.619	254.186	11	1257.620	406.020	12	1273.611	756.044
13	1288.647	394.507	14	1369.718	377.294	15	1401.819	688.790	16	1554.869	279.464	17	1572.898	89787.349	18	1586.912	288.967

BioTools Version 3.2 Copyright © 1999-2008 Bruker Daltonik GmbH Page -2-

(**B**)

Figure S41. Annotated MS spectrum detected in area #10. (**A**) MS spectrum; (**B**) Peak list and sequence data.
